# Nickel-catalyzed stereo-controlled 2,3-hydrosilylation of 1,1-disubstituted allenes†

**DOI:** 10.1039/d5sc01148e

**Published:** 2025-03-24

**Authors:** Jin A Kim, Seoyeon Kim, Shrikant D. Tambe, Jihoon Jang, Eun Jin Cho

**Affiliations:** a Department of Chemistry, Chung-Ang University 84 Heukseok-ro, Dongjak-gu Seoul 06974 Republic of Korea ejcho@cau.ac.kr

## Abstract

Directing regioselectivity and stereoselectivity in allene reactions has long been a significant challenge due to the multiple reactive pathways available. In this study, we report the development of a Ni-catalyzed regio- and stereoselective 2,3-hydrosilylation of 1,1-disubstituted allenes. Stereoselectivity was precisely controlled through the strategic modulation of ligand-induced steric effects and non-covalent interactions. Phenyl dibenzophosphole as the ligand enabled the selective formation of (*Z*)-allylsilanes, while tricyclohexylphosphine favored the production of (*E*)-allylsilanes. This work highlights the critical role of ligand-induced steric and non-covalent interactions in dictating regio- and stereoselectivity, offering new insights into Ni(ii) catalysis for stereoselective hydrosilylation.

## Abstract 2

Ligand-controlled nickel-catalyzed 2,3-hydrosilylations of 1,1-disubstituted allenes enabled the selective synthesis of linear (*Z*)- and (*E*)-allylsilanes.

## Graphical abstract

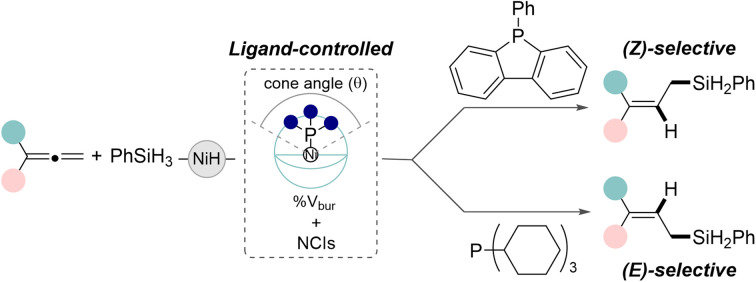

## Introduction

**Scheme 1:**
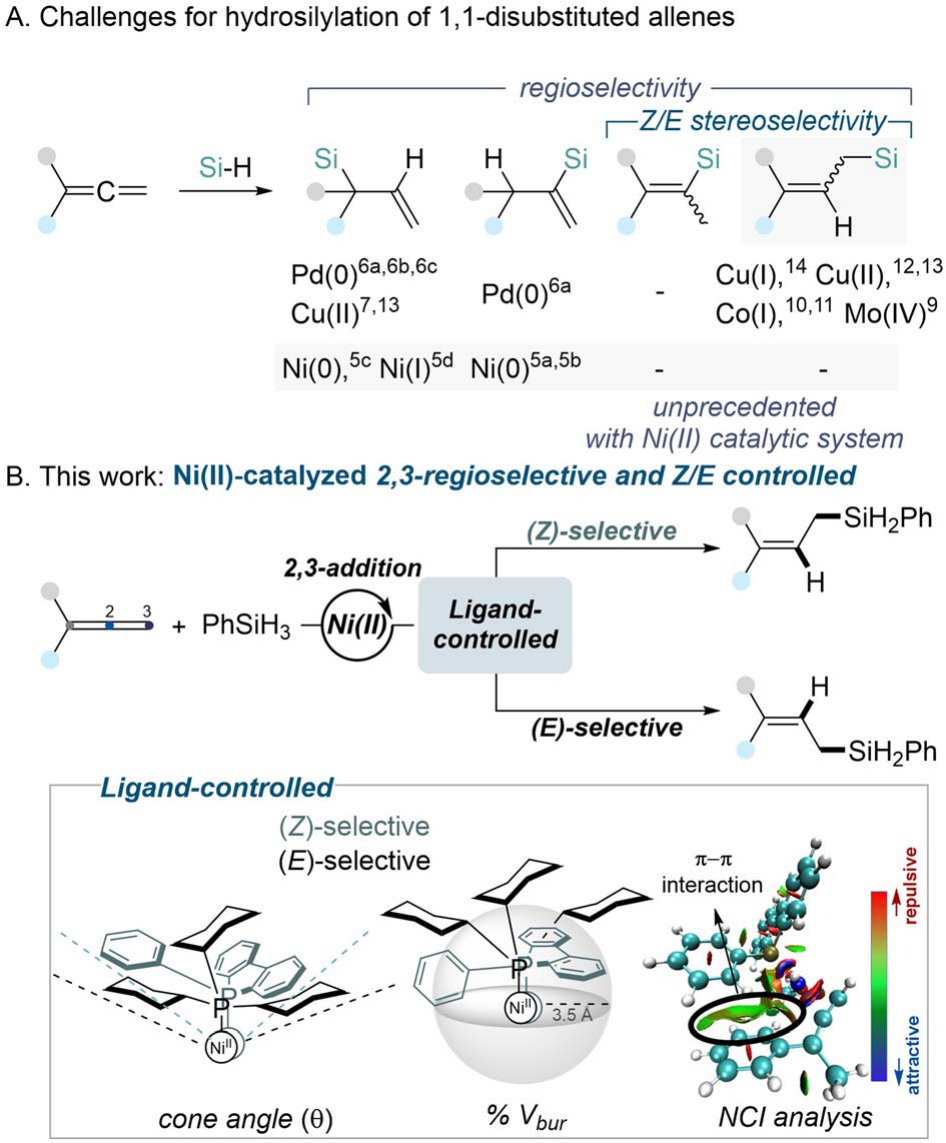
Transition metal-catalyzed hydrosilylation of 1,1-disubstituted allenes.

Although allenes have historically seen less use than other π-systems like alkenes and alkynes, they have recently attracted considerable attention in transition metal-catalyzed reactions. The unique structure of allenes, featuring two orthogonal π-systems, offers a versatile platform for generating diverse regio- and stereoisomers, making them highly appealing in selectivity-focused studies.^1^ Among various allene transformations, hydrofunctionalization reactions stand out for producing regio- and stereoselectively functionalized products.^2–4^ The allene hydrosilylation using silane as a source of both hydrogen and silyl groups is particularly promising, providing access to valuable organosilicon compounds with high atom economy.^5–14^ Allylsilanes or vinylsilanes with specific configurations can be synthesized through various transition metal-catalyzed hydrosilylations of allenes (Scheme 1A).^3,5–13^ In recent decades, nickel—a representative base metal—has gained prominence in organic synthesis. Although Ni(0) and Ni(i) catalytic systems have been employed for 1,2-hydrosilylation, nickel has surprisingly never been used for 2,3-hydrosilylation. As a result, *Z*/*E* stereoselective challenges with Ni catalysis remain unaddressed, and the cost-effective and practical Ni(ii) species have not yet been utilized in any hydrosilylation protocols.

In this study, we present a Ni(ii)-catalyzed hydrosilylation of 1,1-disubstituted allenes, yielding 2,3-hydrosilylated linear allylsilanes with exceptional regio- and stereoselectivity (Scheme 1B). This study unveils the ability of Ni(ii) catalysts to achieve regio- and stereoselectivity through ligand-controlled pathways, providing a cost-effective and mechanistically distinct approach. Subtle modifications in the ligand structure were found to have a significant impact on selectivity. Through extensive ligand analysis, we identified a stereoselectivity threshold influenced by ligand steric effects, with cone angle and percent buried volume (% *V*_bur_) serving as reliable predictive parameters for regio- and stereoselectivity. Additionally, non-covalent π–π interactions were found to play a critical role in determining selectivity.

## Results and discussion

**Scheme 2:**
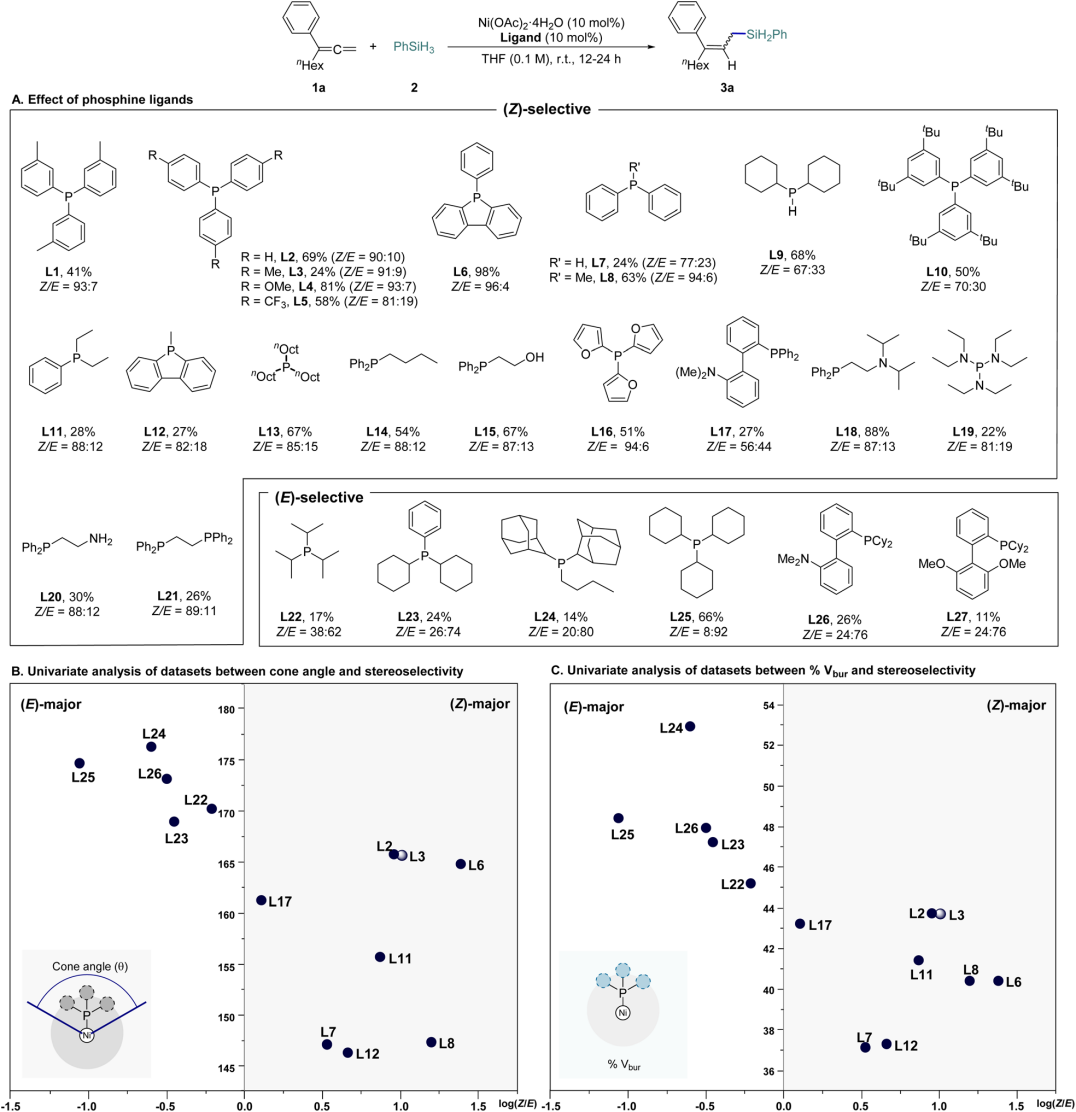
Ligand evaluation for Ni-catalyzed stereo-controlled 2,3-hydrosilylation of 1,1-disubstituted allenes^*a*,*b*,*c*^. ^*a*^Reaction scale: 1a (0.1 mmol), 2 (0.2 mmol). ^*b*^Yields were determined by gas chromatography (GC) spectrometry using *n-*dodecane as an internal standard. ^*c*^The (*Z*) and (*E*) configurations were determined by 1D NOESY.

Our investigation commenced with a comprehensive ligand screening using Ni(OAc)_2_·4H_2_O. We employed nona-1,2-dien-3-ylbenzene as the model substrate (1a) and phenylsilane (2) as the hydride and silyl source, conducting reactions in tetrahydrofuran (THF) at room temperature to synthesize the 2,3-hydrosilylated allylsilane product 3a (Scheme 2A and Tables S1–S3 in ESI† for further optimization details). We evaluated a variety of phosphine-based ligands, including P,N- and P,O-types. Notably, monophosphine ligand, which had previously shown poor reactivity in transition metal-catalyzed hydrosilylation, demonstrated superior reaction efficiency and selectivity (L1–L14, L22–L25). Among the tested ligands, phenyl dibenzophosphole (L6) achieved the best reactivity, yielding (*Z*)-3a in a 96 : 4 *Z*/*E* ratio with 98% yield. In contrast, tricyclohexylphosphine (PCy_3_, L25), which presents greater steric hindrance, shifted selectivity towards (*E*)-3a (*Z*/*E* = 8 : 92), though at a moderate yield of 66%. To elucidate the relationship between ligand structure and stereoselectivity, we initially analyzed the data using cone angle values.^15^ Ligands with relatively large cone angle values predominantly generated the (*E*)-selective product, while ligands with smaller cone angle values preferentially produced the (*Z*)-selective product. However, ligand L6, which exhibited the highest reactivity, did not show a significant difference in cone angle compared to the (*E*)-selective ligands (L22, L23), indicating that cone angle alone could not fully explain the observed trend (Scheme 2B). Consequently, we introduced the percent buried volume (% *V*_bur_) from computational calculations, a parameter that reflects the ligand's conformation, for a more comprehensive analysis. This approach showed that L6 conformed more closely to the expected trend, leading to improved results. Nonetheless, both L25, which demonstrated the best reactivity in the (*E*)-selective products, and L6, which exhibited the highest reactivity in the (*Z*)-selective products, were positioned in the middle of their respective groups, suggesting that factors beyond steric effects may also be contributing to the results (Scheme 2C). Structural analysis of the ligands revealed that those containing aryl moieties generally exhibited superior selectivity.

**Fig. 1:**
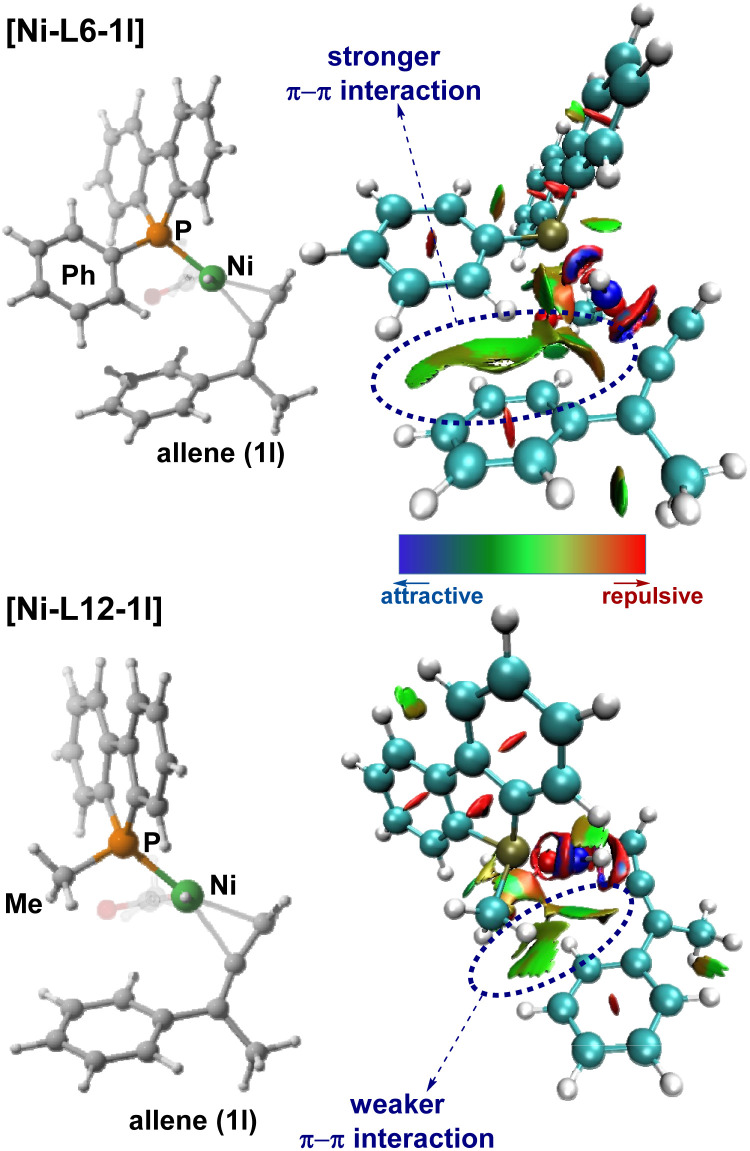
Investigation of the ligand effect: NCI analysis.

To further explore the aryl effect, we conducted a non-covalent interaction (NCI) analysis based on DFT-optimized structures of the Ni–ligand–allene (1l) complex. A comparison between L6 and L12—two structurally similar ligands exhibiting different selectivities and yields—provided key insights. The DFT-calculated 3D structures revealed that L6, which delivered superior selectivity and yield, demonstrated significantly stronger π–π interactions than L12 (Fig. 1). These findings highlight the pivotal role of non-covalent interactions, even among structurally comparable ligands, in influencing reactivity and stereoselectivity.

**Scheme 3:**
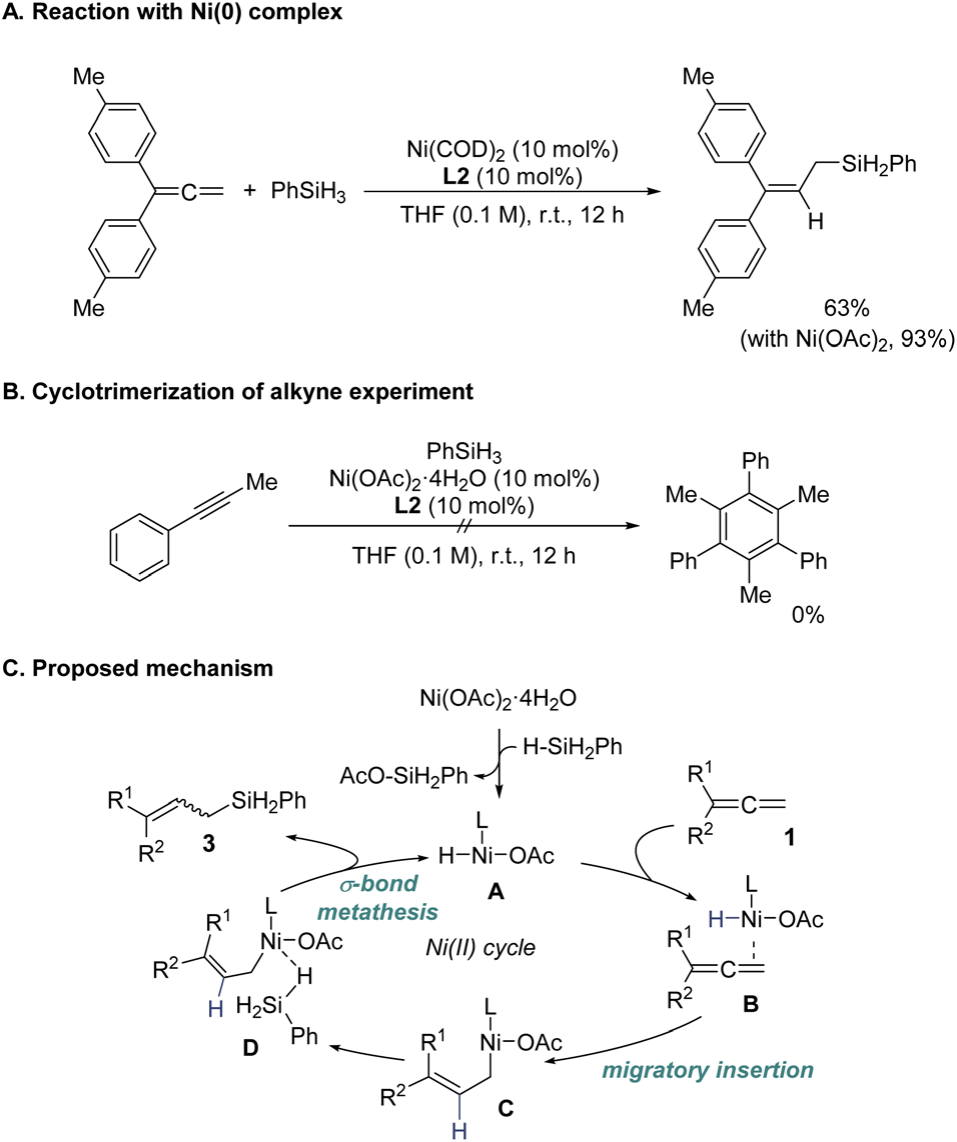
Mechanistic studies and proposed mechanism.

Regarding the mechanism, we initially considered a Ni(0)–Ni(ii) catalytic cycle for the hydrosilylation of allenes based on precedent studies.^5*a*,16^ However, the involvement of such a cycle in the current transformation appears unlikely. When a Ni(0) complex, Ni(COD)_2_, was employed, the reaction exhibited lower efficiency compared to the Ni(ii)-based catalytic system (Scheme 3A). Furthermore, under the optimized catalytic conditions, we confirmed that Ni(0) species were not generated *in situ*. Notably, a characteristic Ni(0)-mediated reaction, such as the cyclotrimerization of alkynes, was not observed when an alkyne substrate was introduced into our catalytic system (Scheme 3B). These observations indicate that Ni(0) complexes are not readily formed under the reaction conditions. Consequently, we propose a redox-neutral Ni(ii) catalytic cycle as the operative pathway for the regio- and stereoselective 2,3-hydrosilylation of 1,1-disubstituted allenes (Scheme 3C). The mechanism begins with the formation of a nickel hydride (Ni–H) intermediate (A) *via* transmetalation between Ni(OAc)_2_·4H_2_O and PhSiH_3_. Migratory insertion of the allene 1 into the Ni–H bond of intermediate A in the coordinated complex B leads to the formation of complex C. Subsequently, PhSiH_3_ coordinates to complex C and undergoes a σ-bond metathesis, yielding the allylsilane product 3 and regenerating the active Ni–H species (A), thus completing the catalytic cycle.

**Scheme 4:**
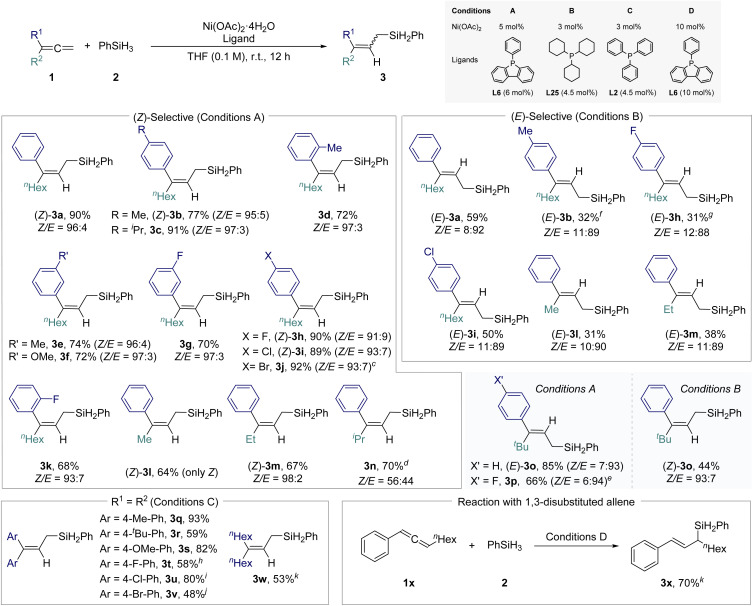
Substrate scope using symmetrical/unsymmetrical 1,1-disubstituted allenes^*a*,*b*^. ^*a*^Reaction scale: 1 (0.4 mmol), 2 (0.8 mmol) were used for reaction. ^*b*^Isolated yields were obtained and the (*Z*) and (*E*) configurations were determined by 1D NOESY. ^*c*^50 °C. ^*d*^41 h. ^*e*^16 h. ^*f*^39 h. ^*g*^48 h. ^*h*^50 °C. ^*i*^Ni cat. (5 mol%), ligand (6 mol%). ^*j*^Ni cat. (10 mol%), ligand (10 mol%), 50 °C, 8 h. ^*k*^1 (0.1 mmol), 2 (0.2 mmol) were used for reaction.

Next, under the optimized reaction conditions, the substrate scope was extensively explored using a range of 1,1-disubstituted allenes, both unsymmetrical and symmetrical, featuring distinct steric and electronic properties (Scheme 4). For the synthesis of (*Z*)-allylsilanes, the optimized catalytic system consisting of 5 mol% Ni(OAc)_2_ and 6 mol% L6 was employed (Conditions A). A variety of 1,1-disubstituted unsymmetrical allenes were examined, incorporating substituents with variable electron densities and positional effects (*ortho-, meta-, para*-), resulting in the formation of products, (*Z*)-3a-(*Z*)-3o. Notably, allene derivatives bearing halogen substituents at the *para*-position afforded relatively high yields ((*Z*)-3h, 3i, 3j). For the synthesis of (*E*)-allylsilanes, 3 mol% Ni(OAc)_2_ and 4.5 mol% L25 were utilized (Conditions B). Although the reaction yields were modest, the products exhibited excellent stereoselectivity.

When varying the aliphatic substituents, the steric effects were pronounced; the presence of a bulky ^*t*^Bu group led to an inversion of (*Z*)/(*E*)-selectivity (3o and 3p).^17^

For symmetrical 1,1-disubstituted allenes, both aromatic and aliphatic, the more cost-effective PPh_3_ (L2) ligand (Conditions C) proved to be an effective alternative, yielding products (3q–3w) with good reactivity.

Additionally, we explored different types of allenes beyond 1,1-disubstituted allenes under the same conditions. The reaction of 1,3-disubstituted allene (1x) showed the same regioselectivity, producing the 2,3-hydrosilylated product (3x). In contrast, reactions with monosubstituted allenes resulted in the formation of 1,2-hydrosilylated branched allylsilanes, emphasizing the significant influence of substrate pattern on regioselectivity (see Scheme S2† for details).

**Scheme 5:**
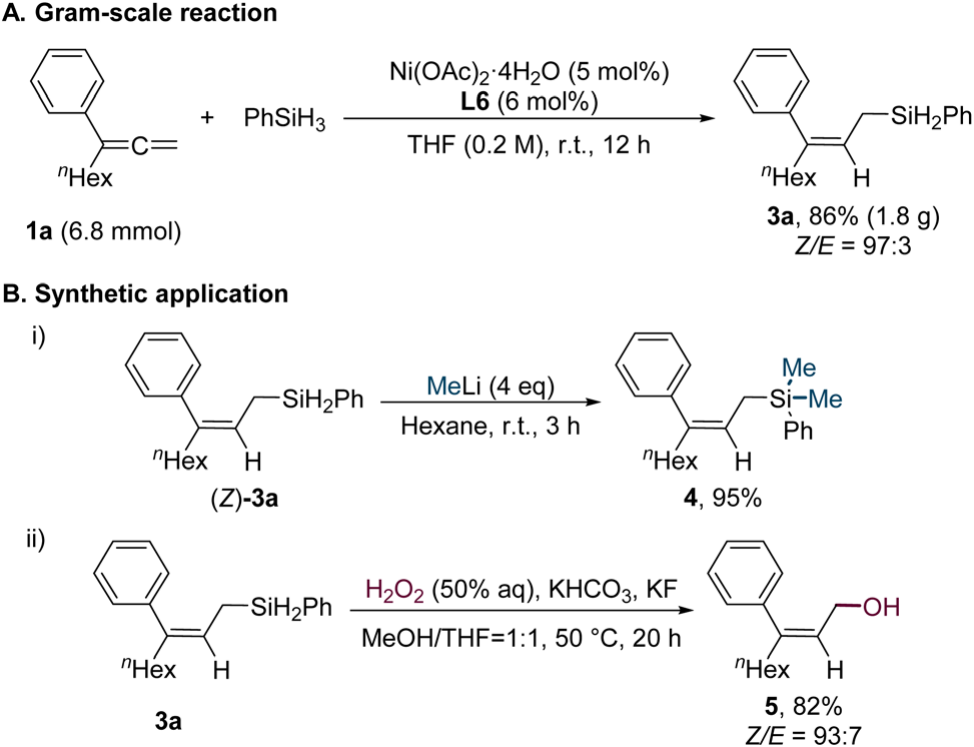
Gram-scale reaction and further synthetic application.

The scalability of the reaction was demonstrated by performing the transformation of substrate 1a with phenylsilane (2) on a 6.8 mmol scale, yielding the desired linear allylsilane 3a in 86% yield with a *Z*/*E* ratio of 97 : 3 (Scheme 5A). The versatility of 3a was further highlighted through its application in subsequent transformations (Scheme 5B). Specifically, treatment of 3a with MeLi resulted in the formation of allylsilane 4 in 95% yield (Scheme 5B-i). Additionally, oxidation of 3a with H_2_O_2_ afforded the corresponding allylic alcohol 5 in 82% yield (Scheme 5B-ii).

## Conclusions

In conclusion, we have developed a mild, ligand-controlled, stereoselective nickel-catalyzed 2,3-hydrosilylation of 1,1-disubstituted allenes, enabling the selective synthesis of linear (*Z*)- and (*E*)-allylsilanes. This study establishes Ni(ii) catalysis as an effective platform for regio- and stereoselective hydrosilylation and reveals the intricate role of ligand-induced steric and non-covalent interactions in dictating selectivity. The correlation between stereoselectivity and two key steric parameters—cone angle and percent buried volume—provides a basis for rational ligand selection in stereoselective catalysis. Moreover, non-covalent interaction analysis provides critical insights into cases where steric effects alone are insufficient to explain the observed selectivity. By demonstrating precise alkene geometry control through rational ligand selection, this work advances stereoselective catalysis and contributes to a broader mechanistic understanding of ligand effects in transition metal-catalyzed transformations.

## Data availability

The data underlying this study are available in the published article and its ESI.†

## Author contributions

J. A K., S. K., S. D. T., and J. J. performed synthetic and mechanistic studies. E. J. C. coordinated the experiments and analyses. All authors analyzed the experimental data and wrote the manuscript.

## Conflicts of interest

The authors declare no competing financial interest.

## Supplementary Material

SC-OLF-D5SC01148E-s001
