# Supplementary material for: Nickel-catalyzed stereo-controlled 2,3-hydrosilylation of 1,1-disubstituted allenes
Source: Chem Sci. 2025 Mar 24;16(17):7489–94. doi: 10.1039/d5sc01148e (PMC11950983; doi:10.1039/d5sc01148e)
Supplement: SC-016-D5SC01148E-s001 [file SC-016-D5SC01148E-s001.pdf]

Supplementary Information (SI) for Chemical Science.
This journal is © The Royal Society of Chemistry 2025

Nickel-Catalyzed Stereo-Controlled 2,3-Hydrosilylation of 1,1-Disubstituted Allenes

Jin A Kim, Seoyeon Kim, Shrikant D. Tambe, Jihoon Jang and Eun Jin Cho*

Department of Chemistry, Chung-Ang University, 84 Heukseok-ro, Dongjak-gu, Seoul 06974, Republic of Korea

E-mail: ejcho@cau.ac.kr (E. J. Cho)

Supplementary Information

General Considerations S-1

Experimental Details S-2

Optimization of Reaction Conditions S-5

Computational Studies S-7

Additional Experiments S-15

Analytic Data for Synthesized Compounds S-15

References S-24

NMR Spectra (^{1}H NMR, ^{13}C NMR and 1D NOESY) S-26

General Considerations

General Reagent Information

All commercially available reagents were purchased from Sigma-Aldrich, Alfa Aesar, Combi blocks or TCI companies and used without further purification. Flash column chromatography was performed using ZEOCHEM ZEOprep silica gel 60 (60-200 mesh).

General Analytical Information

The synthesized 2,3-hydrosilylated allylsilane **3** were characterized by ^{1}H, and $^{13}C\{^{1}H\}$ NMR, and FT-IR spectroscopy. NMR spectra were recorded on a Varian 600 MHz instrument (600 MHz for ^{1}H NMR and 151 MHz for $^{13}C\{^{1}H\}$ NMR or Bruker 400 MHz (400 MHz for ^{1}H NMR, 101 MHz for $^{13}C\{^{1}H\}$ NMR). Copies of ^{1}H NMR and $^{13}C\{^{1}H\}$ NMR spectra can be found in this Supporting Information. ^{1}H NMR experiments are reported in units, parts per million (ppm), and were measured relative to residual $CDCl_3$ (7.26 ppm) in the deuterated solvent. ^{13}C NMR spectra are reported in ppm relative to $CDCl_3$ (77.23 ppm) and all were obtained with ^{1}H decoupling. Coupling constants were reported in Hz. Reactions were monitored by GC-MS using the Agilent GC 7890B/5977A inert MSD with Triple-Axis Detector. Mass spectral data of all unknown compounds were obtained from the Korea Basic Science Institute (Daegu) on a Jeol JMS 700 high resolution mass spectrometer.

Experimental Details

General procedure for synthesis of 1,1-diarylsubstituted allenes (1q – 1v)[1,2]

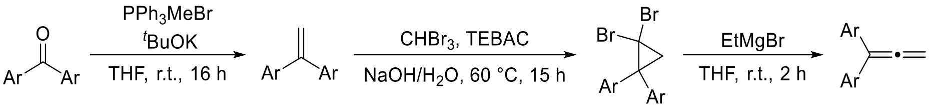

Step 1: In an oven dried flask, was added methyl triphenylphosphonium bromide (1.2 eq) followed by THF (25 mL). Then ^{t}BuOK (1.2 eq) was added, and the resulting yellow suspension was stirred at room temperature. After 1 h, diaryl ketone (5 mmol) was added in one portion and the resulting mixture was further stirred at room temperature and the reaction progress was monitored by thin-layer chromatography. Upon completion after 16 h, the reaction was quenched with water, and the aqueous phase was extracted with diethyl ether (2 × 30 mL). The resulting organic phase was dried over $MgSO_4$, filtered, and concentrated in *vacuo*. The residue was purified by silica gel flash column chromatography using (hexanes) to give the corresponding alkene derivative **S1**.

Step 2: To a solution of alkene **S1** (4 mmol), bromoform (1.5 eq) and $BnNEt_3Cl$ (2 mol%) was added dropwise a solution of NaOH (1 g NaOH dissolved in 2 mL water), and the mixture was stirred at room temperature for 1 h, then heated to 60 °C, and the reaction progress was monitored by thin-layer chromatography. Upon completion (~15 h), the reaction was quenched with water and the aqueous phase was extracted with DCM (2 × 25 mL). The combined organic phase was washed with brine (30 mL). The resulting organic phase was dried over $MgSO_4$, filtered, and concentrated in *vacuo*. The residue was purified by silica gel flash column chromatography using (hexanes/EtOAc, 99/1) to give the corresponding dibromo derivative **S2**.

Step 3: EtMgBr (1.0 M in THF, 1.5 eq) was added dropwise to a pre-cooled ice-bath solution of **S2** (2 mmol) in dry THF (4 mL) under argon atmosphere. After EtMgBr was added, the mixture was then slowly warmed to room temperature, and stirred at room temperature for an additional 2 h. Then the reaction was quenched by NH_4Cl solution, water was added, and the mixture extracted with diethyl ether (2 × 25 mL). The combined organic phase was washed with brine, dried over $MgSO_4$, and filtered. After removing the solvent under reduced pressure, the residue was purified by silica gel flash column chromatography using (hexanes) to afford the corresponding allene (**1q – 1v**).

General procedure for synthesis of 1,1-dialkylsubstituted allenes (1a – 1p)[3]

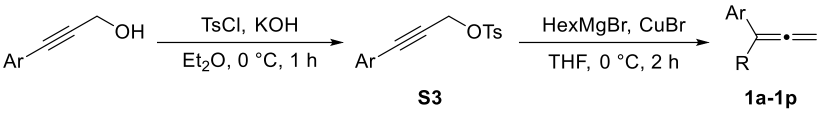

Step 1: TsCl (1.2 eq) was added to a solution of the corresponding propargylic alcohol **S3** (5 mmol) in diethyl ether (12 mL). The reaction mixture was cooled to 0 °C, then KOH (5.5 eq, freshly pestled) was added in small portions. The solution was allowed to warm to room temperature and stirred for 1 h. The reaction progress was monitored by thin-layer chromatography. Upon completion, the reaction was quenched with cold water, and the aqueous phase was extracted with diethyl ether (3 × 15 mL). The combined organic phases were washed with brine and dried over $MgSO_4$. The solvents were removed under reduced pressure and the crude product was purified by silica gel flash column chromatography using (hexanes/ethyl acetate, 20/1) to afford tosylate derivative **S3**.

Step 2: A mixture of CuBr (10 mol%), THF (10 mL) and the tosylate **S3** (4 mmol) was cooled to 0 °C. HexMgBr (1.5 eq, 2 M in THF) was added dropwise. The reaction mixture was allowed to warm to room temperature and stirred for 2 h. Upon completion, the reaction was quenched saturated aqueous solution of NH_4Cl followed by extraction with diethyl ether (3 × 20 mL). The combined organic phases were washed with brine and dried over $MgSO_4$. The solvents were removed under reduced pressure and the crude product was purified by silica gel flash column chromatography using (hexanes) to afford allene derivative (**1a – 1p**).

General procedure for synthesis of 2,3-hydrosilylated allylsilane (3)

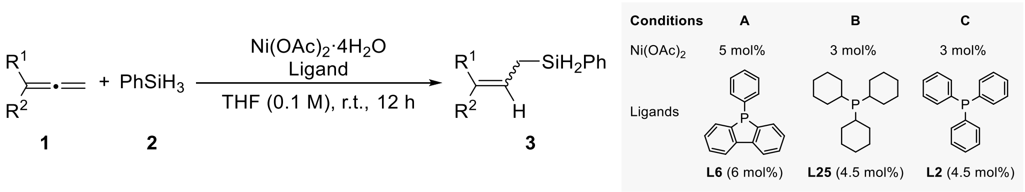

An oven-dried resealable reaction tube equipped with a magnetic stir bar was brought into a glove box. A 1,1-disubstituted allene **1** (0.1 mmol, 1.0 eq), appropriate amounts of $Ni(OAc)_2\cdot 4H_2O$ (3 mol% or 5 mol%) and Ligand (4.5 mol% or 6 mol%), THF (1 mL), and phenylsilane **2** were added to the reaction mixture and the reaction tube was sealed with a silicon septum screw cap, and placed on a pre-heated aluminum heating block at 25 °C. The reaction progress was monitored by thin layer chromatography (TLC) or GC-MS. Upon completion of the reaction, the mixture was concentrated in vacuo to give a crude residue that was purified by flash column

chromatography using hexanes/EtOAc as the eluent to afford the corresponding 2,3-hydrosilylated allylsilane **3**.

Optimization of Reaction Conditions

Table S1. Optimization of 2,3-hydrosilylation of 1,1-disubstisuted allenes 1a for (*Z*)-3a[a,b]

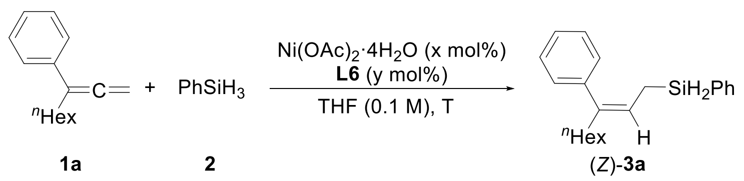

entry	T	variation	time	*Z/E*	yield (%)
1[c]	20 °C	**L2** instead of **L6**	24 h	92:8	62
2[c]	40 °C	**L2** instead of **L6**	2 h	84:16	69
3[c]	r.t.	**L2** instead of **L6**	12 h	90:10	69
4	r.t.	x = 10, y = 10	9 h	96:4	98
5	r.t.	x = 3, y = 4.5	8 h	96:4	86
6	r.t.	x = 5, y = 5	9 h	95:5	56
7	r.t.	x = 5, y = 6	9 h	95:5	90
8	r.t.	x = 5, y = 7.5	9 h	97:3	82

[a]Reaction scale: **1a** (0.1 mmol); **2** (0.2 mmol). [b]The yield was determined by ^{1}H NMR spectroscopy with bromoform as an internal standard. [c]$Ni(OAc)_2·4H_2O$ (10 mol%), **L2** (10 mol%).

Table S2. Optimization of 2,3-hydrosilylation of 1,1-disubstisuted allenes 1a for (*E*)-3a[a,b]

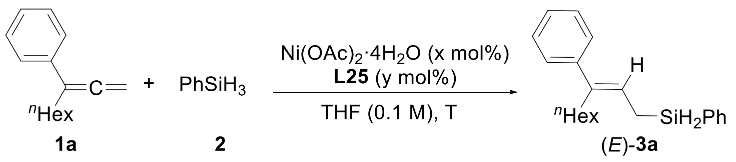

entry	T	variation	time	*E/Z*	yield (%)
1[c]	20 °C	-	6 h	91:9	41
2[c]	40 °C	-	2 h	90:10	46
3	r.t.	x = 10, y = 10	6 h	92:8	66
4	r.t.	x = 3, y = 3.6	8 h	88:12	40
5	r.t.	x = 3, y = 4.5	8 h	89:11	56
6	r.t.	x = 5, y = 5	8 h	93:7	43
7	r.t.	x = 5, y = 6	8 h	93:7	39

[a]Reaction scale: **1a** (0.1 mmol); **2** (0.2 mmol). [b]The yield was determined by ^{1}H NMR spectroscopy with bromoform as an internal standard. [c]$Ni(OAc)_2·4H_2O$ (10 mol%), **L25** (10 mol%).

Table S3. Optimization of 2,3-hydrosilylation of 1,1-disubstisuted allenes 1t[a,b]

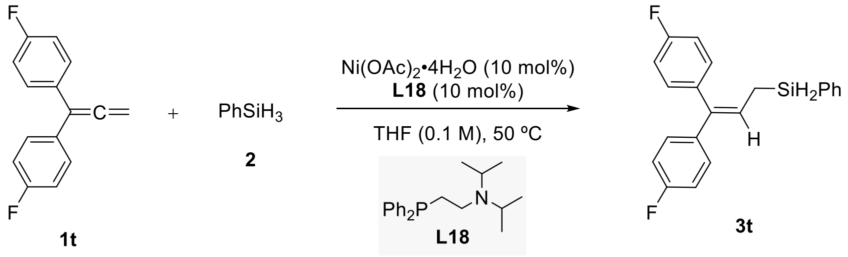

entry	variations	yield (%)
1	-	97
2	$Ni(acac)_2$ instead of $Ni(OAc)_2$•$4H_2O$	56
3	$Ni(COD)_2$ instead of $Ni(OAc)_2$•$4H_2O$	52
4	$NiBr_2$ instead of $Ni(OAc)_2$•$4H_2O$	93
5	$Ni(TFA)_2 \cdot 4H_2O$ instead of $Ni(OAc)_2$•$4H_2O$	90
6	Dioxane instead of THF	93
7	Toluene instead of THF	50
8	MeCN instead of THF	65
9	40 °C instead of 50 ºC	81
10	60 °C instead of 50 ºC	72

[a]Reaction scale: **1t** (0.1 mmol); **2** (0.2 mmol). [b]The yield was determined by ^1H NMR spectroscopy with bromoform as an internal standard.

Computational Studies

General Details

All computations were performed using density functional theory (DFT)[4] implemented in Gaussian 16 suite of program.[5] Geometry optimizations were performed in the gas phase with B3LYP[6] levels of theory with Grimme D3 correction[7-9] and a mixed basis set of LANL2DZ[10] for Ni and 6-31G(d) for other atoms. Three-dimensional molecular structures were visualized using CYLview.[11] Non-covalent interactions were visualized by Multiwfn[12] with .wfn file which was generated from Gaussian 16 quantum chemical package[5] at the B3LYP-D3/6-31G(d)/LANL2DZ(Ni) level of theory. A clear interaction between the metal-ligand system and the substrate can be found in NCI plot. Further visualization of the color-filled RDG isosurface was realized by VMD, where RDG isosurface and color range were set as 0.5, and -0.035 to 0.2, respectively.[13] Percent Buried Volume (% V_{bur}) Calculations The [% V_{bur}] as a steric descriptor provides a measure of the volume occupied by a particular ligand inside a sphere with a radius of 3.5 Å radius centered on the metal center.[14] Values for [% V_{bur}] were calculated using the SambVca 2.1 web application[15] for the [Ni-**L-1l**]. Website for the SambVca 2.1 web application: https://www.molnac.unisa.it/OMtools/sambvca2.1/index.html

NCI analysis of [Ni-L2-1l] and [Ni-L3-1l]

	3D structure of Ni complex	NCI analysis
**[Ni-L2-1l]**	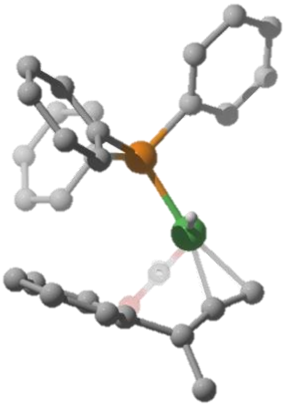	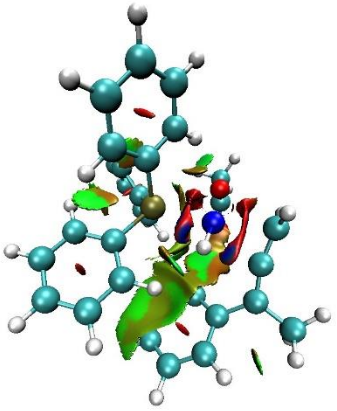

**[Ni-L3-1l]**	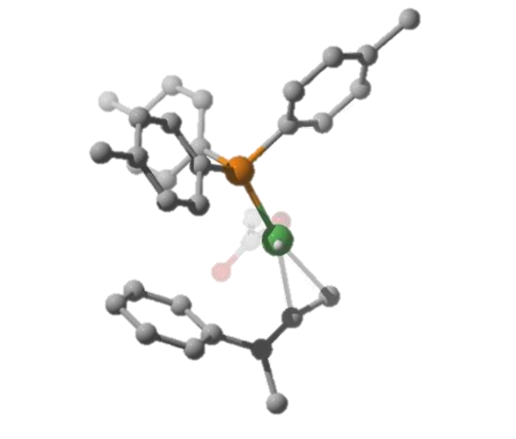	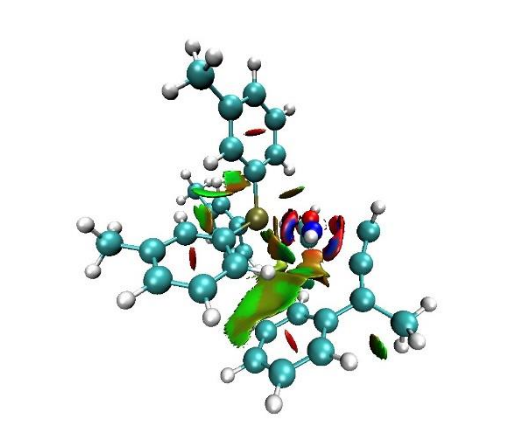

Cartesian Coordinates, Energies of All Optimized Geometries

[Ni-L3-1l]

Ni	0.41011600	-1.29075500	-1.18590200
C	2.21857300	-2.15423700	-2.00337200
C	1.13736300	-2.73995500	-2.54528200
H	0.71576000	-2.38256600	-3.48605400
H	0.74285700	-3.67129500	-2.14545500
C	3.44123900	-1.80589600	-1.65126000
C	3.70578800	-0.78937400	-0.59745800
C	3.02292700	-0.85579000	0.62958400
C	4.63719000	0.23961500	-0.80491100
C	3.25848800	0.10376800	1.61511500
H	2.32600600	-1.67330800	0.80233300
C	4.86690000	1.19697000	0.18493900
H	5.17278200	0.30448100	-1.74818800
C	4.17650800	1.13304300	1.39752100
H	2.72036400	0.04637700	2.55765600
H	5.58449000	1.99404600	0.00698400
H	1.08067700	-0.16509200	-1.86061400
H	4.35132800	1.88211000	2.16500100
C	4.62744300	-2.47963800	-2.31820600
H	4.30397000	-3.22811000	-3.04621400
H	5.25245900	-1.74374300	-2.83932800
P	-0.67627100	0.34404500	-0.19292200
H	5.25709600	-2.96777600	-1.56452300
C	-0.33892400	-3.27910700	0.61459800
C	-1.40315200	-4.14404800	1.27861200
H	-2.05940900	-4.60446200	0.53442700
H	-0.93824700	-4.90818700	1.90564200
H	-2.01848000	-3.49412600	1.91337300
O	0.80378800	-3.21033400	1.08661900
O	-0.76497300	-2.61321600	-0.41523600
C	0.13036500	1.95921000	0.10473900
C	-0.50475700	2.93839000	0.89002600
C	1.39724000	2.23419500	-0.42120500
C	0.10752400	4.16711000	1.11542700
H	-1.47798200	2.73478200	1.32807200
C	2.00550600	3.46749300	-0.18476500
H	1.91936700	1.47487800	-0.99315700
C	1.37476600	4.45225100	0.58206300
H	-0.40005300	4.91616000	1.71935500
H	2.99619000	3.65321400	-0.59126800
C	-1.37923000	-0.09592900	1.44325100
C	-2.71354300	0.15311600	1.78825300
C	-0.53307100	-0.69196200	2.39077900
C	-3.18809800	-0.18836200	3.05613700
H	-3.38878200	0.60465900	1.06774200
C	-1.01665000	-1.03143500	3.65068200
H	0.48923400	-0.93427200	2.12663400
C	-2.35194000	-0.78767600	4.00539100
H	-4.22773000	0.00956900	3.30755700
H	-0.34981900	-1.50877900	4.36491000
C	-2.14131700	0.66596000	-1.24579200
C	-2.91153900	-0.45113600	-1.62072500
C	-2.51074700	1.93499700	-1.70386800
C	-4.03437600	-0.28430400	-2.42524000
H	-2.61925500	-1.43969500	-1.27398100
C	-3.63651500	2.08581900	-2.51707400
H	-1.92729700	2.80798000	-1.42866500
C	-4.41505400	0.98471500	-2.89002000
H	-4.62593500	-1.15434500	-2.70218500
H	-3.91280900	3.07866600	-2.86503900
C	2.04932300	5.77301500	0.86701000
H	2.50806400	5.77184200	1.86481500
H	2.84146100	5.98482000	0.14153500
H	1.33359100	6.60257300	0.84227300
C	-5.61822800	1.14450000	-3.78894800
H	-6.45964700	0.53313600	-3.44295700
H	-5.95175400	2.18632100	-3.83559800
H	-5.38800600	0.82532300	-4.81402500
C	-2.87287800	-1.19495400	5.36326000
H	-3.82594500	-0.70734200	5.59300400
H	-3.03621300	-2.27962800	5.41140900
H	-2.16074400	-0.94152300	6.15698300

[Ni-L6-1l]

Ni	0.66760900	1.23949800	0.59950800
C	2.58382700	1.95900400	1.39936400
C	1.57344000	2.73077200	1.82627300
H	1.10148600	2.55343800	2.79299700
H	1.28918300	3.63088200	1.28358700
C	3.73947000	1.36679400	1.17104600
C	3.86446100	0.18650900	0.27296000
C	3.20763200	0.17385200	-0.96707800
C	4.63622600	-0.92797200	0.64030500
C	3.29847400	-0.93144400	-1.81179700
H	2.61462900	1.02431400	-1.27851700
C	4.73369900	-2.03042400	-0.21025900
H	5.14968500	-0.94305700	1.59762400
C	4.06625600	-2.03566300	-1.43911800
H	2.75253500	-0.91773400	-2.74990000
H	5.33084900	-2.88804600	0.08944800
H	0.86072000	0.19096000	1.63475500
H	4.13951800	-2.89876400	-2.09528000
C	4.99738100	1.91209500	1.82564600
H	4.78613400	2.81041600	2.41132300
H	5.44051400	1.16498900	2.49567400
C	-4.74433300	0.69776100	-2.28766600
C	-3.54711700	0.59554800	-3.00329700

C	-2.34414100	0.33508900	-2.33950300
C	-2.35612000	0.17806200	-0.95349200
C	-3.56525900	0.26335700	-0.22485900
C	-4.75911700	0.53127200	-0.90208300
H	-5.67300700	0.90513600	-2.81272200
H	-3.54768900	0.72242500	-4.08235600
H	-1.40732400	0.28053800	-2.88256900
H	-5.69570800	0.60353000	-0.35619800
C	-2.07751300	-0.25328800	1.59962800
C	-1.74514500	-0.49741300	2.92957100
C	-2.75338800	-0.48342100	3.89757600
C	-4.07545400	-0.20764700	3.53091000
C	-4.40695300	0.05115200	2.19968400
C	-3.40722600	0.02621900	1.22192100
H	-0.71226900	-0.69025900	3.20562100
H	-2.50865100	-0.68020300	4.93764500
H	-4.85305800	-0.18984200	4.28979100
H	-5.43604500	0.27163600	1.93002400
P	-0.94635100	-0.18231900	0.16142200
C	-0.45705800	-1.91217400	-0.19240400
C	-1.38608500	-2.83036700	-0.70903300
C	0.84912400	-2.33200400	0.08876900
C	-1.01039800	-4.15363800	-0.93113300
H	-2.39786800	-2.50949900	-0.93834500
C	1.21933000	-3.65975900	-0.13503700
H	1.57628800	-1.62154300	0.46565300
C	0.29300600	-4.57074000	-0.64269900
H	-1.73362500	-4.85938100	-1.33098200
H	2.23904200	-3.96783700	0.07460900
H	0.58446400	-5.60267300	-0.82044400
H	5.74762200	2.15324400	1.06316500
C	0.30496700	2.25936700	-1.95326300
C	-0.16034600	3.36320900	-2.89378500
H	0.09391800	4.35274100	-2.50576700
H	0.26670500	3.21737300	-3.88899300
H	-1.25297100	3.30032500	-2.97606500
O	0.58215800	1.13272900	-2.39878800
O	0.33250400	2.59115900	-0.70173700

[Ni-L9-1l]

Ni	2.64089100	-0.04383200	-1.03170400
C	4.07381900	-1.70043200	-1.04538700
C	4.28351200	-0.88981400	-2.09340600
H	4.99969500	-0.06922300	-2.03912500
H	3.87256600	-1.14266000	-3.06764700
C	4.05775900	-2.66409700	-0.14832000
C	3.00307900	-2.75070900	0.89843900
C	1.65072600	-2.60217900	0.54224500
C	3.33324200	-2.98831600	2.24199500
C	0.65612500	-2.66895600	1.51981600
H	1.39689200	-2.47056200	-0.50718100
C	2.33428500	-3.05021900	3.21549000
H	4.37381000	-3.10595600	2.53197800
C	0.99329400	-2.88581100	2.85775000
H	-0.38326900	-2.54766500	1.22991600
H	2.60437000	-3.22528200	4.25392600
H	3.49054600	0.27051400	0.13459200
H	0.21741200	-2.93183800	3.61737900
C	5.11817600	-3.74933200	-0.19903800
H	5.80780300	-3.59487800	-1.03295000
H	5.70281700	-3.76997500	0.72914300
P	1.18176400	1.21130500	0.04949200
H	4.64353100	-4.73163400	-0.31066400
C	1.14153000	-1.22623300	-3.10476900
C	0.20539900	-1.08216700	-4.30221500
H	0.45786800	-0.20088500	-4.89887800
H	0.23523600	-1.98445500	-4.91736100
H	-0.81847100	-0.95331400	-3.92863200
O	1.46578600	-2.34503100	-2.69175100
O	1.50665300	-0.09316200	-2.58126800
C	1.19970600	2.89135100	-0.68595100
C	2.42186800	3.37590400	-1.17639400
C	0.05812000	3.70331500	-0.75901800
C	2.50419700	4.65824500	-1.71857900
H	3.30071200	2.73672200	-1.13860500
C	0.14346400	4.98521800	-1.30483600
H	-0.89694400	3.32868600	-0.40288200
C	1.36527700	5.46479900	-1.78253300
H	3.45415700	5.02383000	-2.09869500
H	-0.74580900	5.60737900	-1.36068200
H	1.42796200	6.46159000	-2.21071300
C	1.40389500	1.46789100	1.85164700
C	2.06482000	0.48117700	2.59472900
C	0.86050800	2.58190000	2.51024100
C	2.17406700	0.60276900	3.98091800
H	2.48841700	-0.37882600	2.08999500
C	0.98392600	2.70629100	3.89386900
H	0.35021300	3.35504200	1.94350100
C	1.63789100	1.71501400	4.63174800
H	2.67909300	-0.17660500	4.54415700
H	0.57020100	3.57674200	4.39610200
H	1.72983500	1.81288000	5.71031500
C	-0.55215500	0.64021200	-0.09974400
C	-1.16182800	0.62253800	-1.36437300
C	-1.26767800	0.15297400	1.00489300
C	-2.45577500	0.12972400	-1.51124600
H	-0.61398100	0.97850400	-2.22859800
C	-2.56050200	-0.34122900	0.84982100
H	-0.81116000	0.14554600	1.98901600
C	-3.17712200	-0.36546800	-0.41158800
H	-2.92207500	0.13490700	-2.49292100
H	-3.10679400	-0.70582700	1.71241300
C	-4.53673300	-0.93874300	-0.60144300
C	-4.71767100	-1.89739100	-1.60789000
C	-5.64366800	-0.58310200	0.21562600
C	-5.94195600	-2.53627200	-1.80508000
H	-3.86118200	-2.17404100	-2.21695900
C	-6.86896300	-1.23675400	0.00890000
C	-7.01645000	-2.20892000	-0.98170300
H	-6.04541200	-3.29014300	-2.58006900
H	-7.72782900	-0.97214700	0.61592200
H	-7.97873800	-2.69707500	-1.11280200
N	-5.48877400	0.40558200	1.22517900
C	-5.21527600	1.76224300	0.74949300
H	-6.12114500	2.24796200	0.34480000
H	-4.83973900	2.36909200	1.58131100
H	-4.45216900	1.74971400	-0.02957600
C	-6.45427200	0.39900700	2.31201500
H	-6.07207000	1.02827100	3.12369600
H	-7.44855000	0.79088500	2.02747500
H	-6.57911600	-0.61764400	2.69579300

[Ni-L12-1l]

Ni	-0.67054000	0.10537900	-1.06833600
C	-2.64951400	-0.20133400	-1.90924900
C	-1.75073900	-0.06035500	-2.89668700
H	-1.31249000	-0.93360000	-3.38091600
H	-1.53664000	0.91656000	-3.32655900
C	-3.74333800	-0.41741100	-1.20320200
C	-3.76060500	-0.45825700	0.28309900
C	-3.01970400	0.46580400	1.03769000
C	-4.53127600	-1.41693300	0.96224100
C	-3.03527000	0.41951600	2.43167700
H	-2.43715900	1.23739800	0.54676800
C	-4.53979400	-1.46497100	2.35629700
H	-5.11449500	-2.14179300	0.40188000
C	-3.79155800	-0.54654100	3.09771900
H	-2.45675200	1.15137000	2.98806200
H	-5.13386900	-2.22058200	2.86385300
H	-0.80957000	-1.33946500	-0.77223300
H	-3.80575600	-0.58049100	4.18389700
C	-5.05288900	-0.63543300	-1.94527600
H	-4.92955900	-0.48603100	-3.02076800
H	-5.42659400	-1.65449700	-1.78616600
C	5.01055400	1.93886800	0.56296200
C	3.87630300	2.70576200	0.84656200

C	2.60294300	2.12909100	0.81000500
C	2.47835700	0.77773100	0.48738000
C	3.62358700	-0.00959000	0.22320000
C	4.88996900	0.58276900	0.25683100
H	5.99532600	2.39784100	0.58844100
H	3.98192000	3.75776900	1.09713800
H	1.71921900	2.71444200	1.03731800
H	5.77831700	-0.00881300	0.05352400
C	1.94758400	-1.73254600	0.02961900
C	1.48916400	-3.02854100	-0.19605600
C	2.41175600	-4.04297400	-0.46595900
C	3.77865600	-3.74973700	-0.53129800
C	4.23878600	-2.44868700	-0.32353000
C	3.32404700	-1.42946900	-0.03707700
H	0.42403400	-3.24234400	-0.17373900
H	2.06594700	-5.05882400	-0.63563000
H	4.49002800	-4.54095900	-0.75236000
H	5.30177100	-2.23320400	-0.38589500
P	0.95616100	-0.23761300	0.37723100
H	-5.81881400	0.05667400	-1.57612300
C	-0.42809200	2.81047100	-0.47360600
C	-0.23442500	4.26073500	-0.89647900
H	-0.75403700	4.47014100	-1.83516500
H	-0.57545300	4.93588500	-0.10840700
H	0.83693400	4.43144000	-1.06227400
O	-0.47950800	2.51221300	0.73501500
O	-0.48202300	1.96736300	-1.45013400
C	0.35844500	-0.38856200	2.11206600
H	1.17327900	-0.68894900	2.77639000
H	-0.02953100	0.59308000	2.39076300
H	-0.45878000	-1.11251200	2.15053200

[Ni-L2-1l]

Ni	0.43659400	-1.18759300	-0.94652800
C	2.40028900	-1.88349400	-1.55693800
C	1.44201000	-2.66437200	-2.08287900
H	1.04303500	-2.47131900	-3.07993700
H	1.14497200	-3.59215400	-1.59932100
C	3.53775600	-1.33233800	-1.18017300
C	3.58845600	-0.18196100	-0.23789200
C	2.83452200	-0.21313900	0.94823700
C	4.38554600	0.94033600	-0.51042400
C	2.86627400	0.87022000	1.82733900
H	2.24502200	-1.09894200	1.17531100
C	4.41234900	2.02081900	0.37341900
H	4.97431500	0.97917000	-1.42292700
C	3.65067500	1.99009500	1.54398400
H	2.27413900	0.83800500	2.73817100
H	5.02776800	2.88757700	0.14565200
H	1.00318400	-0.06360400	-1.71294400
H	3.66730000	2.83418600	2.22793700
C	4.84716600	-1.90101200	-1.69758200
H	4.67871400	-2.76050600	-2.35156300
H	5.40433000	-1.14447000	-2.26403400
P	-0.92303500	0.38096900	-0.22396700
H	5.48045100	-2.21325300	-0.85845100
C	-0.15941100	-3.07278100	1.01687100
C	-1.13215300	-4.02190500	1.70519800
H	-1.67197600	-4.63213400	0.97540100
H	-0.60459900	-4.65647700	2.42082300
H	-1.86910000	-3.41705000	2.24828300
O	0.93101500	-2.79929900	1.53599300
O	-0.60773800	-2.56641600	-0.09150000
C	-0.36410100	2.12067100	-0.08332900
C	-1.19141700	3.08367500	0.52225500
C	0.89888500	2.49966600	-0.55384400
C	-0.76917700	4.40742100	0.62691400
H	-2.16150000	2.79438400	0.91643400
C	1.32074300	3.82619200	-0.44145600
H	1.55932300	1.75333300	-0.98144700
C	0.48843100	4.78120900	0.14181400
H	-1.41692400	5.14551800	1.09223900
H	2.30946600	4.10112000	-0.79630200
H	0.81959900	5.81261100	0.23034200
C	-1.68264300	0.04068300	1.41305900
C	-3.05721500	0.18273100	1.65054300
C	-0.83854900	-0.35578700	2.46086300
C	-3.57821100	-0.06545800	2.92227900
H	-3.72249000	0.47401200	0.84339200
C	-1.36372200	-0.60611800	3.72809300
H	0.21623700	-0.51778900	2.27374800
C	-2.73355100	-0.45945700	3.96208600
H	-4.64538500	0.04309700	3.09699000
H	-0.70286000	-0.93155200	4.52650900
H	-3.14225300	-0.66002500	4.94888100
C	-2.33828600	0.37147200	-1.39205900
C	-2.93015900	-0.87906600	-1.65045900
C	-2.81905600	1.51523800	-2.04133400
C	-4.00135500	-0.97046000	-2.53750900
H	-2.53937000	-1.76498700	-1.15523800
C	-3.88880300	1.41261100	-2.93476100
H	-2.36393200	2.48257200	-1.85409800
C	-4.48249500	0.17395000	-3.18139800
H	-4.45786900	-1.93759700	-2.73052300
H	-4.25663800	2.30364000	-3.43664800
H	-5.31506400	0.09859200	-3.87584500

[Ni-L8-1l]

Ni	0.31495600	-1.26227700	-1.15528200
C	2.26907200	-2.18772000	-0.89178500
C	1.50635300	-2.93176800	-1.70798700
H	1.56169500	-2.81176700	-2.79107900
H	0.91920200	-3.76542400	-1.32920300
C	3.23207000	-1.69175800	-0.14043700
C	3.07095900	-0.45048300	0.66551000
C	1.93838900	-0.27768300	1.48005400
C	4.05444200	0.55096500	0.64190700
C	1.79959300	0.88089600	2.24609600
H	1.17954000	-1.05701400	1.51832000
C	3.90455100	1.70978200	1.40581000
H	4.93422200	0.43304600	0.01512400
C	2.77662400	1.87752900	2.21274600
H	0.92241100	1.00183600	2.87659900
H	4.67107500	2.47997200	1.37170900
H	1.21968000	-0.27018800	-1.77931400
H	2.66014000	2.77960800	2.80719500
C	4.55867400	-2.42845500	-0.06206000
H	4.54429300	-3.34260800	-0.66113900
H	5.37804800	-1.79518200	-0.42409900
P	-1.05017700	0.45030700	-1.14078000
H	4.78319000	-2.69027800	0.97890100
C	-1.22468100	-2.73309400	0.65384600
C	-2.48962900	-3.51899100	0.97675000
H	-2.71217800	-4.25061400	0.19494500
H	-2.39480400	-4.01057100	1.94771200
H	-3.32598000	-2.81043000	1.02361000
O	-0.47009500	-2.35954900	1.56184100
O	-1.07422600	-2.46709100	-0.60770100
C	-0.41022900	2.14020800	-0.83047700
C	-1.31037600	3.21169600	-0.69524100
C	0.96507300	2.38066300	-0.73244000
C	-0.83720800	4.50530600	-0.48732800
H	-2.38135800	3.02992300	-0.73927300
C	1.43561600	3.67916100	-0.52193600
H	1.66251300	1.55292300	-0.79856400
C	0.53932600	4.74088700	-0.40345400
H	-1.53946100	5.32838100	-0.38535000
H	2.50448900	3.84848000	-0.43369100
H	0.90776800	5.74969600	-0.23641000
C	-2.44820900	0.29719600	0.03243200
C	-3.78798300	0.22260100	-0.37176700
C	-2.13835400	0.21746800	1.39945000
C	-4.80281600	0.07935800	0.57785100
H	-4.05289500	0.26642600	-1.42336300
C	-3.15237500	0.07434000	2.34377000
H	-1.10238500	0.22934500	1.71910100

C	-4.48729000	0.00664700	1.93554600
H	-5.83829200	0.02005800	0.25346000
H	-2.89747300	-0.00156400	3.39683000
H	-5.27722900	-0.11092000	2.67242400
C	-1.82199200	0.57975300	-2.80994800
H	-2.53804400	1.40552500	-2.86437800
H	-1.01941000	0.75097300	-3.53182700
H	-2.31792000	-0.36438300	-3.05323300

[Ni-L14-1l]

Ni	0.36129900	-0.74510500	-0.99548200
C	2.33743500	-1.66195800	-1.27228700
C	1.41593900	-2.32031000	-1.98908600
H	1.24370700	-2.08087900	-3.03841900
H	0.91481800	-3.19588700	-1.57911300
C	3.43103900	-1.20128500	-0.69825700
C	3.41135300	-0.07545700	0.27582500
C	2.44752700	-0.04662600	1.29522000
C	4.36051400	0.95793500	0.21487100
C	2.42279900	0.99250700	2.22491000
H	1.71398200	-0.83863700	1.36956300
C	4.33373900	1.99821000	1.14447400
H	5.11373900	0.95881700	-0.56809000
C	3.36692600	2.01826200	2.15440600
H	1.65763300	0.98430200	2.99552000
H	5.06972500	2.79542400	1.07919500
H	0.90159500	0.26529500	-1.93504600
H	3.35314500	2.82817300	2.87911700
C	4.76601700	-1.85679000	-1.00988000
H	4.64480700	-2.70994600	-1.68232800
H	5.44723600	-1.14139400	-1.48702900
P	-1.08619500	0.87950500	-0.68339700
H	5.24609100	-2.19846300	-0.08503300
C	-0.75849400	-1.76828500	1.38307900
C	-1.50249500	-2.88712400	2.10005400
H	-1.10022200	-3.86717400	1.82867700
H	-1.46183000	-2.74029500	3.18169700
H	-2.55193200	-2.85294800	1.78193400
O	-0.57889300	-0.67507600	1.94011900
O	-0.39599700	-2.06193900	0.17098900
C	-2.70809500	0.33056700	-0.03186800
C	-3.22344200	-0.87579100	-0.53239100
C	-3.45805300	1.05540800	0.90400500
C	-4.47024300	-1.34077300	-0.11369100
H	-2.62401600	-1.46822300	-1.21733800
C	-4.70296000	0.58546600	1.32629200
H	-3.07202400	1.97931200	1.32204800
C	-5.21303900	-0.61024100	0.81701600
H	-4.85499500	-2.27872700	-0.50515300
H	-5.27164700	1.15267200	2.05832100
H	-6.18113100	-0.97510700	1.14955700
C	-1.56104800	1.74059600	-2.26260900
H	-0.62316200	2.06121500	-2.72815900
H	-1.97665300	0.96076000	-2.91205900
C	-0.45528900	2.21637700	0.42031500
H	-1.25059100	2.94862900	0.60092100
H	-0.22899700	1.71070500	1.36226700
C	0.78937200	2.90566500	-0.15355400
H	1.18394700	3.62604700	0.57015600
H	1.57952500	2.18015700	-0.36394100
H	0.56238900	3.44797500	-1.07878100
C	-2.54248100	2.90787100	-2.11560700
H	-2.80580800	3.31251600	-3.09963100
H	-3.46765000	2.59185900	-1.62213400
H	-2.10815200	3.72494600	-1.52856700

[Ni-L7-1l]

Ni	0.34889900	-1.27060700	-0.90690100
C	2.34986700	-2.21335300	-0.94405200
C	1.44212000	-2.98851600	-1.55357200
H	1.30200200	-2.94481400	-2.63387800
H	0.92163100	-3.77342400	-1.00692500
C	3.43346700	-1.65551700	-0.44225600
C	3.39750700	-0.36456800	0.29746100
C	2.40624800	-0.13899800	1.26440300
C	4.34986000	0.63946800	0.05798400
C	2.35179100	1.06603800	1.96339100
H	1.66496600	-0.90020600	1.47093300
C	4.29922400	1.84262100	0.76367300
H	5.12310000	0.48917900	-0.69019800
C	3.30194000	2.05907700	1.72011300
H	1.55257000	1.21652400	2.68227600
H	5.04004300	2.61292700	0.56462700
H	0.94477300	-0.42035100	-1.97131600
H	3.26462700	2.99846300	2.26533500
C	4.77061500	-2.36098600	-0.59380200
H	4.65999600	-3.32438000	-1.09803400
H	5.46690900	-1.74801700	-1.17935300
P	-1.20656200	0.25274700	-1.16193900
H	5.22657800	-2.52465200	0.38993100
C	-0.87239600	-1.81492900	1.52070300
C	-1.73020900	-2.70118000	2.41300900
H	-1.48914400	-3.75863600	2.27808700
H	-1.61403600	-2.40924800	3.45963500
H	-2.77944500	-2.54952400	2.12962200
O	-0.61504900	-0.64774500	1.85818700
O	-0.50088700	-2.36421800	0.40630400
C	-0.76375300	1.98971000	-0.80389200
C	-1.74676300	2.98917200	-0.71743900
C	0.58543300	2.32792100	-0.63764200
C	-1.37848100	4.31135700	-0.47431300
H	-2.79602200	2.73229600	-0.83011800
C	0.94844100	3.65478100	-0.39833100
H	1.34518400	1.55574600	-0.68272100
C	-0.02986200	4.64592500	-0.31662800
H	-2.14341700	5.08030700	-0.40708400
H	1.99684700	3.90018300	-0.26001300
H	0.25403900	5.67728300	-0.12415900
C	-2.87119200	-0.03590300	-0.45527700
C	-3.81980100	-0.73140500	-1.21949000
C	-3.19648400	0.36584800	0.84888800
C	-5.07524400	-1.02741700	-0.68731900
H	-3.58136900	-1.04629800	-2.23288600
C	-4.45664400	0.07570100	1.37291200
H	-2.45802900	0.87398800	1.45683900
C	-5.39604000	-0.62292700	0.61017600
H	-5.80170100	-1.56856100	-1.28742400
H	-4.70053700	0.39049900	2.38387400
H	-6.37416300	-0.85099200	1.02516900
H	-1.56650800	0.30168300	-2.52802500

[Ni-L22-1l]

Ni	0.01247800	0.83004100	-0.87116500
C	-2.09013100	1.26028500	-1.41871700
C	-1.26879100	2.16266900	-1.97063300
H	-0.84781200	2.01408100	-2.96529400
H	-1.14724300	3.13725400	-1.50441400
C	-3.09823500	0.53927400	-0.97613100
C	-2.92681700	-0.51883100	0.05730200
C	-2.19191300	-0.24030500	1.22278600
C	-3.52061500	-1.78135500	-0.08936600
C	-2.05147500	-1.21555000	2.21198000
H	-1.77911400	0.75770000	1.35115900
C	-3.36614200	-2.75653200	0.89731100
H	-4.09342200	-2.00979100	-0.98409100
C	-2.63141500	-2.47693700	2.05226800
H	-1.50032700	-0.98095800	3.11958700
H	-3.82279800	-3.73391600	0.76488700
H	-0.28345600	-0.24247800	-1.83345100
H	-2.52165500	-3.23297600	2.82542400
C	-4.50044500	0.82493400	-1.48454000
H	-4.50443400	1.65042100	-2.20106300
H	-4.92637500	-0.05802600	-1.97723300
P	1.58507000	-0.61926400	-0.29325700
H	-5.15792800	1.08243400	-0.64539000
C	-0.10871900	3.01832100	0.97612200
C	0.65280500	4.14857200	1.66562400

H	1.31539500	4.65102200	0.95327900
H	-0.04215000	4.86584600	2.10716400
H	1.28687300	3.72948300	2.45667500
O	-1.33035700	2.89906700	1.11163300
O	0.65970100	2.22922400	0.28199100
C	1.34792600	-2.35749700	-0.95042300
H	2.12617600	-2.97495200	-0.48576700
C	1.68540800	-0.84333900	1.57481600
H	0.62325100	-0.85282600	1.84687700
C	3.24164000	-0.08490400	-0.99776100
H	3.04957600	-0.17671400	-2.07495500
C	-0.03095200	-2.89015200	-0.53082300
H	-0.82834900	-2.29596900	-0.98587600
H	-0.14513500	-3.92999900	-0.86110700
H	-0.19012500	-2.86329100	0.55066900
C	1.51733100	-2.43817900	-2.47484500
H	1.28679200	-3.45548400	-2.81392400
H	0.83010600	-1.75184200	-2.98234500
H	2.53805800	-2.20856000	-2.79766100
C	4.42353000	-0.99855900	-0.63760100
H	5.31076700	-0.69575500	-1.20781300
H	4.67564300	-0.91700400	0.42490300
H	4.22902000	-2.05251000	-0.86237800
C	3.57124300	1.39479200	-0.72892200
H	4.37730200	1.71105700	-1.40289000
H	2.70335400	2.04132100	-0.87598600
H	3.91833200	1.54860000	0.29638900
C	2.31681700	-2.15664600	2.06750900
H	3.36353100	-2.24876500	1.76035700
H	2.29469900	-2.17116700	3.16451400
H	1.77984800	-3.04361300	1.72018600
C	2.33354100	0.36731700	2.26761300
H	2.12735400	0.32196700	3.34429200
H	3.42230200	0.35558000	2.14429900
H	1.94422500	1.30703700	1.87205700

[Ni-L23-1l]

Ni	0.31997000	-1.42139000	-0.32973400
C	2.19473900	-2.51069100	-0.75193300
C	1.13576800	-3.33031400	-0.80757200
H	0.61726900	-3.51670600	-1.74943700
H	0.85229000	-3.94329600	0.04500000
C	3.39165600	-1.96030200	-0.76149500
C	3.62450100	-0.54171100	-0.37409600
C	3.08641700	-0.04754400	0.82567000
C	4.39438800	0.31010800	-1.18133100
C	3.31209100	1.27842800	1.20054000
H	2.49769300	-0.71064600	1.45542200
C	4.61115900	1.63635200	-0.80366000
H	4.81195400	-0.05761200	-2.11471400
C	4.07199800	2.12432300	0.39027400
H	2.88733400	1.64794200	2.12968700
H	5.20312400	2.28790000	-1.44165700
H	0.79177900	-0.79706500	-1.57978100
H	4.24309600	3.15662500	0.68373700
C	4.59670700	-2.80368300	-1.14049500
H	4.30830900	-3.83109000	-1.37753800
H	5.10737400	-2.38031700	-2.01429800
P	-0.99347600	0.34021900	-0.31184500
H	5.31951800	-2.81971300	-0.31579300
C	-0.00431300	-2.28564100	2.34008200
C	-0.87506200	-2.90883600	3.42886900
H	-1.38940600	-3.79826500	3.05227200
H	-0.27258900	-3.15853800	4.30471400
H	-1.64691300	-2.18667100	3.72447300
O	1.13090100	-1.87016800	2.60579200
O	-0.58826800	-2.21200400	1.18383300
C	-2.57448800	-0.12710700	-1.17932500
C	-2.26002800	-0.55493600	-2.62851400
C	-3.30883900	-1.24947100	-0.41543700
H	-3.22611400	0.75760900	-1.21034700
C	-3.52885900	-1.00214400	-3.36868300
H	-1.54009200	-1.38478500	-2.59640900
H	-1.76813900	0.26454700	-3.16615900
C	-4.56872900	-1.70005500	-1.17169000
H	-2.62597300	-2.09602500	-0.27380400
H	-3.58418100	-0.91043800	0.58993800
C	-4.24431500	-2.12874400	-2.60963000
H	-3.27020600	-1.32541500	-4.38487500
H	-4.21058200	-0.14492100	-3.47434800
H	-5.05096700	-2.52061200	-0.62581100
H	-5.29204200	-0.87096800	-1.19784400
H	-5.16031500	-2.42469200	-3.13626900
H	-3.59279600	-3.01485700	-2.58282700
C	-1.45871900	0.81593800	1.42632200
C	-0.19773600	1.24388200	2.20383200
C	-2.57857200	1.86127500	1.56367600
H	-1.80051500	-0.13837900	1.84567000
C	-0.53078400	1.49913700	3.68105900
H	0.21491200	2.15753300	1.75246900
H	0.56348700	0.46377900	2.12781700
C	-2.90938700	2.10164100	3.04747900
H	-2.25179000	2.80695300	1.11106400
H	-3.48171500	1.54241700	1.02770100
C	-1.66237500	2.52499700	3.83823200
H	0.36909700	1.83507700	4.21151500
H	-0.82770000	0.54717000	4.14441200
H	-3.69660700	2.86147700	3.13498500
H	-3.31526900	1.17439200	3.47826300
H	-1.91347400	2.66112900	4.89778800
H	-1.31781100	3.50175100	3.46682600
C	-0.42752600	1.88355500	-1.12243000
C	0.94371500	2.06626100	-1.35329100
C	-1.32077400	2.90761900	-1.48351200
C	1.41380500	3.25201500	-1.92024600
H	1.64029700	1.27828700	-1.09309400
C	-0.84941500	4.08700200	-2.06061300
H	-2.38783900	2.78944600	-1.32194300
C	0.52004300	4.26280600	-2.27652900
H	2.48069600	3.37432400	-2.08089200
H	-1.55146500	4.86747800	-2.34188800
H	0.88578600	5.18322800	-2.72413000

[Ni-L24-1l]

Ni	2.41855400	0.51239600	-0.91482700
C	3.94297400	-0.78381500	-1.83023700
C	4.09103400	0.48785300	-2.23192700
H	4.74890000	1.17760600	-1.70091200
H	3.69067600	0.81774200	-3.18739800
C	4.02164600	-2.08928200	-1.66030600
C	3.01072700	-2.84757000	-0.87548800
C	1.64279000	-2.63792500	-1.11677600
C	3.39722800	-3.77611300	0.10256700
C	0.68404400	-3.33552300	-0.38114800
H	1.35167400	-1.93436800	-1.89245100
C	2.43449500	-4.46800700	0.83977300
H	4.45173400	-3.94535700	0.30336900
C	1.07528300	-4.25057900	0.59887100
H	-0.36983000	-3.15195200	-0.56945600
H	2.74676800	-5.17615200	1.60308100
H	3.15045800	-0.15107600	0.17044400
H	0.32699700	-4.79045500	1.17320600
C	5.15491200	-2.86044700	-2.31285400
H	5.81367100	-2.19888100	-2.88148200
H	5.75875500	-3.37899800	-1.55769600
P	0.99245200	0.91829700	0.71464600
H	4.75130000	-3.62416300	-2.98864600
C	0.93109800	1.01242700	-3.20939800
C	0.21968600	1.94489000	-4.18365700
H	0.82489800	2.83622400	-4.37614700
H	-0.00292600	1.42635100	-5.11850400
H	-0.72067300	2.28050300	-3.72882200
O	0.96353300	-0.21155100	-3.40855200
O	1.43658000	1.61070800	-2.17673300
C	-0.69790100	0.25088600	0.47094800
C	-1.04515400	-0.33692700	-0.75077500

C	-1.67638200	0.34052700	1.47794100
C	-2.33769600	-0.82143900	-0.95852400
H	-0.31502000	-0.41486400	-1.54774700
C	-2.96315200	-0.14134000	1.26723200
H	-1.43911400	0.79578000	2.43588300
C	-3.31775200	-0.73182100	0.04053700
H	-2.59322000	-1.25886900	-1.91995500
H	-3.71137500	-0.05250900	2.04674300
C	-4.68190800	-1.27904400	-0.18832500
C	-4.80279300	-2.58762600	-0.67473200
C	-5.86188100	-0.53936500	0.09584200
C	-6.04543000	-3.19673000	-0.85022100
H	-3.89417700	-3.14760100	-0.87976900
C	-7.10589500	-1.16637200	-0.08020700
C	-7.19902800	-2.48179400	-0.53749900
H	-6.10632900	-4.21930200	-1.21121500
H	-8.01813000	-0.61601200	0.12228200
H	-8.17845700	-2.93662000	-0.66127000
N	-5.76200600	0.79932100	0.56172400
C	-5.20266700	1.76579400	-0.38554400
H	-5.93357400	2.04945200	-1.16334400
H	-4.90320300	2.67115000	0.15467300
H	-4.31746300	1.35868600	-0.87498800
C	-6.91009000	1.34127900	1.27006700
H	-6.60424100	2.26189300	1.77969500
H	-7.76157300	1.59354400	0.61110700
H	-7.25507700	0.62917800	2.02536900
C	0.78151800	2.77625600	0.90269200
C	2.09936600	3.49603400	0.53276100
C	-0.37481800	3.32900800	0.04179500
H	0.55479300	2.96218400	1.96464800
C	1.97112900	5.01706600	0.70808100
H	2.32370100	3.26802800	-0.51676000
H	2.93627600	3.11444500	1.12791500
C	-0.50034500	4.85288000	0.20218600
H	-0.17171000	3.07648300	-1.00602500
H	-1.32042300	2.84894100	0.31424000
C	0.81356000	5.57240100	-0.13325200
H	2.91532100	5.50237900	0.42975100
H	1.79711200	5.25012100	1.76956400
H	-1.31289400	5.22320700	-0.43612100
H	-0.78490900	5.08551000	1.23985500
H	0.70897500	6.65363100	0.02337800
H	1.04271000	5.42412000	-1.19835000
C	1.44652200	0.29238600	2.41330800
C	1.49073400	-1.24874600	2.40030700
C	2.77338400	0.88890500	2.92061300
H	0.64599300	0.61764900	3.09433000
C	1.90323000	-1.82305000	3.76232600
H	2.20838800	-1.57880100	1.63976200
H	0.51827600	-1.65149600	2.10065000
C	3.17634600	0.29379400	4.28058500
H	3.56089300	0.68041800	2.18358300
H	2.69458200	1.97916300	3.00710200
C	3.24307600	-1.23873200	4.22845500
H	1.96347500	-2.91583900	3.68695700
H	1.12841900	-1.59672500	4.51041200
H	4.14182700	0.71256500	4.59174400
H	2.44154300	0.59702800	5.04150600
H	3.51993100	-1.64197400	5.21085300
H	4.03177500	-1.54285100	3.52454800

[Ni-L25-1l]

Ni	0.06849100	-1.20935900	-0.75823900
C	1.78314000	-2.39695300	-1.48953400
C	0.61909200	-2.94848300	-1.85926700
H	0.14433500	-2.69327200	-2.80791700
H	0.19655000	-3.77767100	-1.29671300
C	3.04755100	-2.12064400	-1.24361900
C	3.45895900	-1.03195000	-0.31530800
C	2.91692800	-0.98390000	0.97927300
C	4.40805700	-0.07160000	-0.69668600
C	3.32943200	0.00452800	1.87444000
H	2.17936200	-1.72885600	1.26656300
C	4.80332100	0.92545000	0.19657600
H	4.82898600	-0.09510800	-1.69844600
C	4.27078900	0.96125200	1.48846000
H	2.91137200	0.02382200	2.87707400
H	5.53042200	1.67110700	-0.11457200
H	0.89491900	-0.28722100	-1.55674700
H	4.58862100	1.73060900	2.18736700
C	4.13939700	-2.96788800	-1.87247900
H	3.72160900	-3.74711400	-2.51543000
H	4.81247600	-2.34696100	-2.47697000
P	-0.91589600	0.64869800	-0.15817800
H	4.74696900	-3.44123100	-1.09144400
C	-0.83186500	-2.97031000	1.18508100
C	-1.92996100	-3.71477900	1.93913300
H	-2.60982000	-4.20917200	1.23789200
H	-1.49712700	-4.44504600	2.62578300
H	-2.52432200	-2.99197500	2.51203400
O	0.35543700	-3.07747200	1.52189300
O	-1.27002300	-2.22229800	0.21947200
C	-2.56669600	0.72496900	-1.06307800
C	-2.42221700	0.07206100	-2.45989000
C	-3.72673500	0.04584300	-0.30210100
H	-2.80963200	1.79097800	-1.19243500
C	-3.72699200	0.16212300	-3.26445000
H	-2.15902000	-0.98473700	-2.31354100
H	-1.59633900	0.51902200	-3.02081200
C	-5.03623600	0.11552700	-1.10668400
H	-3.45305400	-0.99948700	-0.11087500
H	-3.88756900	0.52224200	0.67084800
C	-4.88629700	-0.49652400	-2.50543000
H	-3.59042700	-0.31109200	-4.24519400
H	-3.96635600	1.21928800	-3.45486000
H	-5.83407900	-0.39214300	-0.54980400
H	-5.34219500	1.16821200	-1.20459100
H	-5.82274400	-0.39730100	-3.06883600
H	-4.68502100	-1.57332700	-2.40829200
C	-0.08461300	2.27847300	-0.53849600
C	1.30817600	2.32682000	0.12282600
C	0.02296000	2.53544400	-2.05633000
H	-0.71929900	3.06449800	-0.10319400
C	2.07084700	3.61747300	-0.20730500
H	1.89189100	1.46408500	-0.21871000
H	1.21860900	2.22952100	1.20802400
C	0.79354000	3.82983700	-2.36570500
H	0.54148300	1.68696100	-2.52303500
H	-0.97427600	2.59177500	-2.50734800
C	2.18572600	3.82372600	-1.72159800
H	3.06502600	3.57015300	0.25318600
H	1.55090500	4.47914300	0.23821000
H	0.86996400	3.95657800	-3.45314100
H	0.22424700	4.69206600	-1.98714800
H	2.71604700	4.75787800	-1.94615700
H	2.78216100	3.00509900	-2.15039000
C	-1.33618900	0.65739800	1.66062400
C	-0.18724800	0.18194200	2.57352600
C	-1.93670300	1.97672800	2.18146700
H	-2.10144700	-0.12608200	1.71249000
C	-0.69942100	-0.00047000	4.01085700
H	0.62372500	0.91877200	2.57641400
H	0.23103000	-0.75507100	2.19977300
C	-2.44336600	1.80337500	3.62397000
H	-1.16440700	2.75857600	2.16449100
H	-2.75173300	2.32539700	1.53434100
C	-1.33063400	1.29149900	4.55189000
H	0.12332700	-0.32608400	4.65966400
H	-1.44578500	-0.80854000	4.02502200
H	-2.84668700	2.75376600	3.99646200
H	-3.27621000	1.08470900	3.62440500
H	-1.72459300	1.13345400	5.56378600
H	-0.55096900	2.06372300	4.63422800

[Ni-L26-1l]

Ni	-0.05213100	-1.43063100	-0.58414400
C	1.32804700	-2.92158400	-1.34120100
C	0.13065300	-3.10387500	-1.92082600
H	-0.06988400	-2.69620600	-2.91071000
H	-0.63361800	-3.73988000	-1.47260700
C	2.60004700	-3.02946000	-1.00252100
C	3.29897800	-2.12849800	-0.04398100
C	2.69268700	-1.73928200	1.16350100
C	4.59665200	-1.66911400	-0.32310000
C	3.36215000	-0.89142900	2.04770100
H	1.70321400	-2.11447000	1.40537300
C	5.25858100	-0.81618800	0.56067400
H	5.08730600	-1.95904500	-1.24752500
C	4.64232000	-0.41901400	1.74953300
H	2.88163200	-0.60634600	2.98074400
H	6.25715300	-0.46143300	0.31889100
H	0.16873500	-0.58770500	-1.75843800
H	5.15898900	0.24215200	2.44010700
C	3.41171800	-4.15301100	-1.63327200
H	2.77624000	-4.81284000	-2.22897800
H	4.19404900	-3.75336600	-2.29045800
P	-0.58442700	0.52755500	0.29302400
H	3.90756900	-4.74575200	-0.85522000
C	-1.15236600	-3.46212200	1.11404100
C	-1.03805600	-4.24441300	2.41941500
H	-1.17546400	-3.57125100	3.27292900
H	-1.77918100	-5.04538500	2.45371000
H	-0.03135800	-4.67015800	2.50366500
O	-1.94916400	-3.80481900	0.23226700
O	-0.35333200	-2.43676500	1.05503500
C	-2.34983600	1.07085200	-0.04622600
H	-2.31122300	2.16737400	0.02990400
C	-3.41774000	0.57353600	0.96563600
C	-2.82130400	0.70318100	-1.48459100
H	-3.10095100	0.79764400	1.98961500
C	-3.65877200	-0.94011800	0.81432500
C	-4.73217600	1.33520800	0.67870900
H	-2.06134300	1.00307600	-2.21535400
C	-3.08851500	-0.81064100	-1.61901900
C	-4.13727100	1.46427300	-1.76267400
H	-4.39826400	-1.26787600	1.55746500
H	-2.73959600	-1.49916000	1.00270700
C	-4.15905700	-1.25623400	-0.60803300
H	-4.57887700	2.41639300	0.80535600
H	-5.49473600	1.03117200	1.40868600
C	-5.21883700	1.02735700	-0.75166500
H	-2.17211000	-1.39322800	-1.45640600
H	-3.41584000	-1.02585900	-2.64554100
H	-3.97054500	2.54887900	-1.69511400
H	-4.47277100	1.25431300	-2.78723900
H	-4.31407900	-2.33651200	-0.69896500
C	-5.46903600	-0.49064000	-0.88415400
H	-6.14866800	1.57662300	-0.94976600
H	-5.83771500	-0.72373300	-1.89271300
H	-6.24882800	-0.80382400	-0.17610900
C	0.39117400	2.08580000	-0.07960300
H	0.04039700	2.76043700	0.71468000
C	0.11151500	2.82204600	-1.42060700
C	1.92704100	1.92032800	0.08519900
H	-0.96802200	2.92807300	-1.57837800
C	0.74555200	2.09740100	-2.62512300
C	0.73477900	4.23438000	-1.30749300
H	2.15426500	1.35478300	0.99462200
C	2.54611900	1.18987200	-1.12361100
C	2.54863000	3.33120300	0.18927200
H	0.53081600	2.66832400	-3.53924800
H	0.30698800	1.10289600	-2.75290000
C	2.26919100	1.97763700	-2.41852500
H	0.27678000	4.78386700	-0.47294700
H	0.51917700	4.80266200	-2.22260400
C	2.26027400	4.12555900	-1.10160200
H	2.14382600	0.17683000	-1.20250500
H	3.62570000	1.08854300	-0.96092400
H	2.14319000	3.86632000	1.05969800
H	3.63244300	3.24166300	0.34367300
H	2.71263900	1.44720900	-3.27128000
C	2.88424700	3.38760500	-2.30493400
H	2.69140300	5.13193900	-1.01897700
H	3.97300600	3.31470300	-2.17634000
H	2.70517300	3.95615200	-3.22820900
C	-0.43541400	0.38689900	2.13858500
H	0.58856100	0.04146800	2.31610300
H	-1.06101700	-0.47231000	2.38988000
C	-0.75551500	1.62749600	3.00315900
H	-1.39073900	1.32109000	3.84494000
H	-1.35207400	2.36049000	2.44253800
C	0.49413000	2.32232200	3.56206500
H	1.16818700	2.58988800	2.73965900
H	1.04813700	1.60650000	4.18559200
C	0.16032600	3.57390500	4.37820500
H	1.06492400	4.04331200	4.78083900
H	-0.35633900	4.31896000	3.75999300
H	-0.49672500	3.33218900	5.22278900

Additional Experiments

Scheme S1. Ligand evaluation of ^{t}Bu-substituted substrate

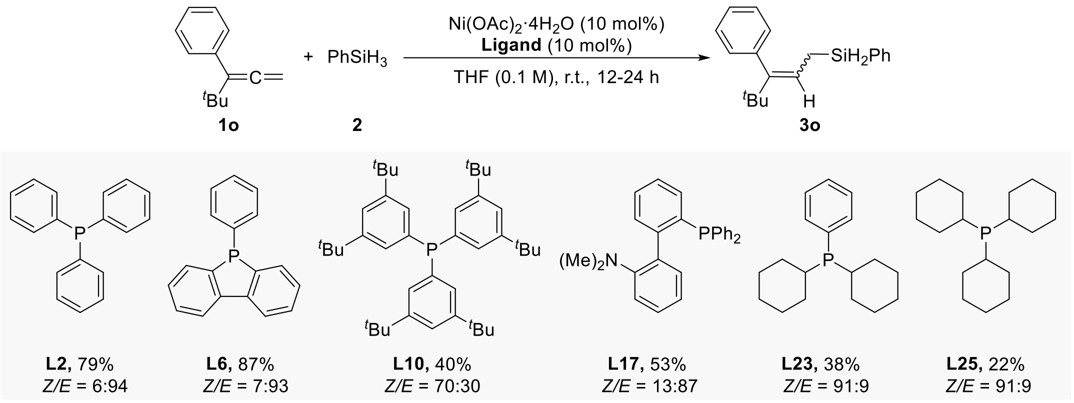

Scheme S2. Reactions of mono-substituted allenes

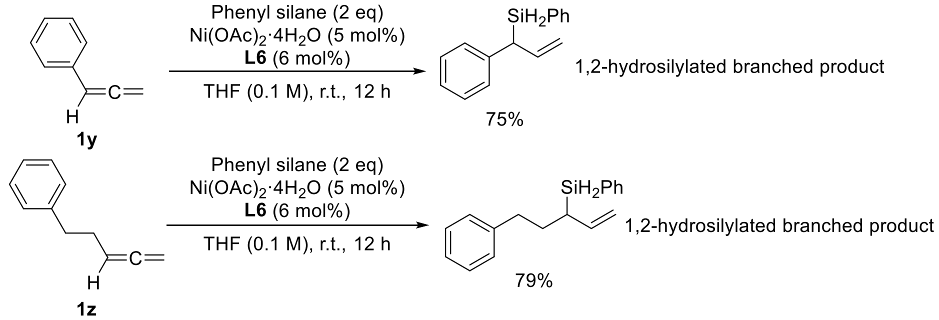

Analytic Data for Synthesized Compounds

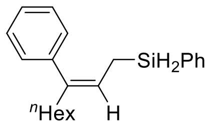

(*Z*)-phenyl(3-phenylnon-2-en-1-yl)silane, (*Z*)-**3a** (111 mg, 90%): colorless liquid; **^{1}H NMR (600 MHz, CDCl$_3$)** δ 7.50 (d, *J* = 7.4 Hz, 2H), 7.40 (t, *J* = 7.5 Hz, 1H), 7.34 (dd, *J* = 7.5, 7.4 Hz, 2H), 7.29 (dd, *J* = 7.3, 7.2 Hz, 2H), 7.22 (t, *J* = 7.3 Hz, 1H), 7.03 (d, *J* = 7.2 Hz, 2H), 5.50 (t, *J* = 8.4 Hz, 1H), 4.27 (t, *J* = 3.6 Hz, 2H), 2.30 (t, *J* = 7.0 Hz, 2H), 1.76 (dt, *J* = 8.4, 3.6 Hz, 2H), 1.29 – 1.21 (m, 8H), 0.86 (t, *J* = 7.0 Hz, 3H); **^{13}C NMR (151 MHz, CDCl$_3$)** δ 141.43, 141.29, 135.50, 132.30, 129.84, 128.69, 128.25, 128.13, 126.47, 121.36, 39.69, 31.90, 28.97, 28.31, 22.86, 14.31, 13.05; **IR (neat)**: ν_{max} = 2926, 2136, 933, 864, 842, 700 cm^{-1}; **HRMS** m/z (EI) calc. for $C_{21}H_{28}Si$ [M^{+}] 308.1960, found 308.1960; ***R$_f$*** 0.70 (Hexane).

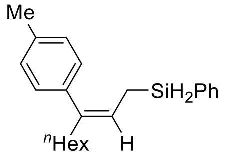

(Z)-phenyl(3-(p-tolyl)non-2-en-1-yl)silane, (Z)-**3b** (99 mg, 77%): colorless liquid; **^{1}H NMR (600 MHz, $CDCl_3$)** δ 7.52 (d, J = 7.1 Hz, 2H), 7.40 (t, J = 7.4 Hz, 1H), 7.35 (dd, J = 7.4, 7.1 Hz, 2H), 7.11 (d, J = 7.7 Hz, 2H), 6.94 (d, J = 7.8 Hz, 2H), 5.49 (t, J = 8.3 Hz, 1H), 4.28 (t, J = 3.6 Hz, 2H), 2.35 (s, 3H), 2.29 (t, J = 6.7 Hz, 2H), 1.78 (dt, J = 8.3, 3.6 Hz, 2H), 1.28 – 1.22 (m, 8H), 0.87 (t, J = 7.0 Hz, 3H); **^{13}C NMR (151 MHz, $CDCl_3$)** δ 141.17, 138.36, 135.95, 135.50, 132.37, 129.81, 128.96, 128.55, 128.11, 121.13, 39.75, 31.90, 28.98, 28.35, 22.87, 21.37, 14.31, 13.03; **IR (neat)**: ν_{max} = 2958, 2926, 2135, 933, 837, 700 cm^{-1}; **HRMS** m/z (EI) calc. for $C_{22}H_{30}Si$ [M^+] 322.2117, found 322.2117; ***R_f*** 0.60 (Hexane).

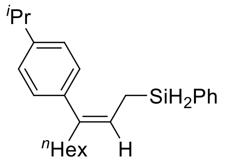

(Z)-(3-(4-isopropylphenyl)non-2-en-1-yl)(phenyl)silane, **3c** (127 mg, 91%): colorless liquid; **^{1}H NMR (400 MHz, $CDCl_3$)** δ 7.52 (d, J = 6.3 Hz, 2H), 7.40 (t, J = 7.3 Hz 1H), 7.34 (dd, J = 7.3, 6.3 Hz, 2H), 7.15 (d, J = 8.1, 2H), 6.98 (d, J = 8.1, 2H), 5.49 (t, J = 8.2 Hz, 1H), 4.29 (t, J = 3.5 Hz, 2H), 2.91 (hept, J = 7.0 Hz, 1H), 2.29 (t, J = 6.8 Hz, 2H), 1.79 (dt, J = 8.2, 3.5 Hz, 2H), 1.30 – 1.23 (m, 14H), 0.87 (t, J = 6.8 Hz, 3H); **^{13}C NMR (101 MHz, $CDCl_3$)** δ 146.86, 141.21, 138.68, 135.50, 132.42, 129.80, 128.51, 128.11, 126.24, 121.08, 39.70, 33.95, 31.91, 29.01, 28.40, 24.22, 22.88, 14.33, 13.01; **IR (neat)**: ν_{max} = 2926, 2135, 933, 864, 837, 701 cm^{-1}; **HRMS m/z (EI)** calc. for $C_{24}H_{34}Si$ [M^+] = 350.2430, found 350.2430; ***R_f*** 0.48 (Hexane).

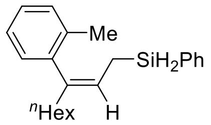

(Z)-phenyl(3-(*o*-tolyl)non-2-en-1-yl)silane, **3d** (93 mg, 72%): colorless liquid; **^{1}H NMR (400 MHz, $CDCl_3$)** δ 7.48 (d, J = 7.3 Hz, 2H), 7.38 (t, J = 7.2 Hz, 1H), 7.33 (dd, J = 7.3, 7.2 Hz, 2H), 7.20 – 7.14 (m, 2H), 7.11 (dd, J = 7.3, 7.1 Hz, 1H), 6.82 (d, J = 7.4 Hz, 1H), 5.52 (t, J = 8.0 Hz, 1H), 4.22 (t, J = 3.8 Hz, 2H), 2.21 (t, J = 6.7 Hz, 2H), 2.18 (s, 3H), 1.58 (dt, J = 8.0, 3.8 Hz, 2H), 1.30 – 1.22 (m, 8H), 0.88 (t, J = 6.8 Hz, 3H). **^{13}C NMR (101 MHz, $CDCl_3$)** δ 140.96, 140.90, 135.58, 135.46, 132.38, 130.05, 129.80, 129.44, 128.10, 126.65, 125.57, 121.47, 39.08, 31.99, 29.31, 28.17, 22.88, 19.50, 14.33, 13.02; **IR (neat)**: ν_{max} = 2925, 2135, 933, 880, 840, 702 cm^{-1}; **HRMS m/z (EI)** calc. for $C_{22}H_{30}Si$ [M^+] = 322.2117, found 322.2117; **R_f** 0.48 (Hexane).

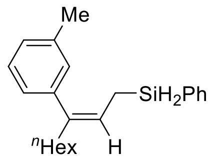

(Z)-phenyl(3-(*m*-tolyl)non-2-en-1-yl)silane, **3e** (95 mg, 74%): colorless liquid; **^{1}H NMR (400 MHz, $CDCl_3$)** δ 7.50 (d, J = 6.8 Hz, 2H), 7.38 (t, J = 7.3 Hz, 1H), 7.33 (dd, J = 7.3, 6.8 Hz , 2H), 7.17 (dd, J = 7.6, 7.5 Hz, 1H), 7.02 (d, J = 7.5 Hz, 1H), 6.83 (d, J = 7.6 Hz, 1H), 6.80 (s, 1H), 5.47 (t, J = 8.6 Hz, 1H), 4.27 (t, J = 3.7 Hz, 2H), 2.30 (s, 3H), 2.26 (t, J = 6.9 Hz, 1H), 1.77 (dt, J = 8.6, 3.7 Hz, 2H), 1.32 – 1.16 (m, 8H), 0.86 (t, J = 6.8 Hz, 3H); **^{13}C NMR (101 MHz, $CDCl_3$)** δ 141.39, 141.38, 137.69, 135.52, 132.37, 129.81, 129.32, 128.11, 128.09, 127.21, 125.73, 121.12, 39.70, 31.90, 28.99, 28.33, 22.86, 21.69, 14.32, 13.11;

IR (neat): ν_{max} = 2925, 2136, 934, 860, 842, 700 cm^{-1}; **HRMS m/z (EI)** calc. for $C_{22}H_{30}Si$ [M^+] = 322.2117, found 322.2117; **R*f*** 0.48 (Hexane).

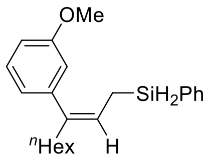

(*Z*)-3-(1-(phenylsilyl)non-2-en-3-yl)phenol, **3f** (97 mg, 72%): colorless liquid; **^{1}H NMR (400 MHz, $CDCl_3$)** δ 7.52 (d, *J* = 7.2 Hz, 2H), 7.40 (t, *J* = 7.3 Hz, 1H), 7.35 (dd, *J* = 7.3, 7.2 Hz, 1H), 7.22 (dd, *J* = 8.2, 7.8 Hz, 1H), 6.78 (d, *J* = 8.2 Hz, 1H), 6.65 (d, *J* = 7.8 Hz, 1H), 6.63 (s, 1H), 5.50 (t, *J* = 8.2 Hz, 1H), 4.30 (t, *J* = 3.5 Hz, 2H), 3.78 (s, 3H), 2.30 (t, *J* = 6.6 Hz, 1H), 1.80 (dt, *J* = 8.2, 3.5 Hz, 2H), 1.35 – 1.19 (m, 8H), 0.88 (t, *J* = 6.8 Hz, 3H). **^{13}C NMR (101 MHz, $CDCl_3$)** δ 159.57, 142.93, 141.14, 135.49, 132.28, 129.85, 129.24, 128.13, 121.42, 121.17, 114.20, 112.02, 55.32, 39.64, 31.90, 28.99, 28.33, 22.86, 14.32, 13.11; **IR (neat)**: ν_{max} = 2925, 2134, 932, 837, 781, 700 cm^{-1}; **HRMS m/z (EI)** calc. for $C_{21}H_{25}OSi$ [M^+] = 338.2066, found 338.2066; **R*f*** 0.38 (Hexane).

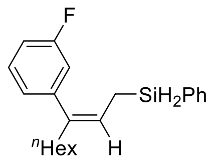

(*Z*)-(3-(3-fluorophenyl)non-2-en-1-yl)(phenyl)silane, **3g** (91 mg, 70%): colorless liquid; **^{1}H NMR (400 MHz, $CDCl_3$)** δ 7.49 (d, *J* = 7.5 Hz, 2H), 7.40 (t, *J* = 7.3 Hz, 1H), 7.34 (dd, *J* = 7.5, 7.3 Hz, 2H), 7.25 – 7.20 (m, 1H), 6.93 – 6.87 (m, 1H), 6.79 (d, $J_{H\text{-}F}$ = 7.6 Hz, 1H), 6.69 (d, *J* = 9.9 Hz, 1H), 5.51 (t, *J* = 8.1 Hz, 1H), 4.27 (t, *J* = 3.6 Hz, 2H), 2.26 (d, *J* = 6.6 Hz, 2H), 1.75 (dt, *J* = 8.1, 3.6 Hz, 2H), 1.29 – 1.20 (m, 8H), 0.86 (t, *J* = 6.8 Hz, 3H). **^{13}C NMR (101 MHz, $CDCl_3$)** δ 162.91 (d, $J_{C–F}$ = 245.6 Hz), 143.73 (d, $J_{C–F}$ = 7.6 Hz), 140.09 (d, $J_{C–F}$ = 1.8 Hz), 135.48, 132.02, 129.94, 129.68 (d, $J_{C–F}$ = 8.4 Hz), 128.18, 124.43 (d, $J_{C–F}$ = 2.8 Hz), 122.20, 115.52 (d, $J_{C–F}$ = 20.7 Hz), 113.36 (d, $J_{C–F}$ = 21.0 Hz), 39.48, 31.86, 28.92, 28.25, 22.84, 14.30, 13.18; **IR (neat)**: ν_{max} = 2925, 2138, 1580, 933, 843, 701 cm^{-1}; **HRMS m/z (EI)** calc. for $C_{21}H_{27}FSi$ [M^+] = 326.1866, found 326.1866; ***Rf*** 0.47 (Hexane).

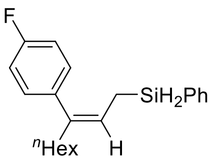

(*Z*)-(3-(4-fluorophenyl)non-2-en-1-yl)(phenyl)silane, (*Z*)-**3h** (117 mg, 90%): colorless liquid; **^{1}H NMR (600 MHz, $CDCl_3$)** δ 7.50 (d, *J* = 7.2 Hz, 2H), 7.41 (t, *J* = 7.4 Hz, 1H), 7.35 (dd, *J* = 7.4, 7.2 Hz, 2H), 7.00 – 6.94 (m, 4H), 5.52 (t, *J* = 8.4 Hz, 1H), 4.27 (t, *J* = 3.6 Hz, 2H), 2.27 (t, *J* = 6.2 Hz, 2H), 1.74 (dt, *J* = 8.4, 3.6 Hz, 2H), 1.29 – 1.20 (m, 8H), 0.87 (t, *J* = 7.0 Hz, 3H); **^{13}C NMR (151 MHz, $CDCl_3$)** δ 161.66 (d, $J_{C\text{-}F}$ = 244.8 Hz), 140.25, 137.14 (d, $J_{C\text{-}F}$ = 3.5 Hz), 135.49, 132.14, 130.17 (d, $J_{C\text{-}F}$ = 7.5 Hz), 129.91, 128.17, 121.90, 115.12 (d, $J_{C\text{-}F}$ = 21.1 Hz), 39.74, 31.88, 28.93, 28.23, 22.85, 14.30, 13.12; **IR (neat)**: ν_{max} = 2926, 2134, 1507, 863, 836, 701 cm^{-1}; **HRMS** m/z (EI) calc. for $C_{22}H_{16}F_3$ [M^+] 326.1866, found 326.1868; ***Rf*** 0.47 (Hexane).

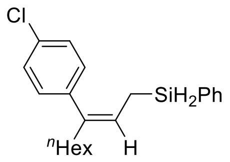

(Z)-(3-(4-chlorophenyl)non-2-en-1-yl)(phenyl)silane, (Z)-**3i** (122 mg, 89%): colorless liquid; **^{1}H NMR (600 MHz, CDCl$_3$)** δ 7.49 (d, J = 7.2 Hz, 2H), 7.41 (t, J = 7.4 Hz, 1H), 7.35 (dd, J = 7.4, 7.2 Hz, 2H), 7.25 (d, J = 8.0 Hz, 2H), 6.92 (d, J = 8.0 Hz, 2H), 5.52 (t, J = 8.4 Hz, 1H), 4.27 (t, J = 3.6 Hz, 2H), 2.27 (t, J = 6.7 Hz, 2H), 1.74 (dt, J = 8.4, 3.6 Hz, 2H), 1.30 – 1.18 (m, 8H), 0.87 (t, J = 7.1 Hz, 3H); **^{13}C NMR (151 MHz, CDCl$_3$)** δ 140.06, 139.76, 135.49, 132.24, 132.05, 130.07, 129.94, 128.48, 128.18, 122.15, 39.55, 31.87, 28.92, 28.23, 22.84, 14.30, 13.19; **IR (neat)**: ν_{max} = 2926, 2137, 1489, 932, 835, 701 cm^{-1}; **HRMS** m/z (EI) calc. for $C_{21}H_{27}ClSi$ [M$^+$] 342.1571, found 342.1571; $\boldsymbol{R_f}$ 0.76 (Hexane).

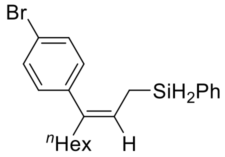

(Z)-(3-(4-bromophenyl)non-2-en-1-yl)(phenyl)silane, **3j** (142 mg, 92%): colorless liquid; **^{1}H NMR (400 MHz, CDCl$_3$)** δ 7.49 (d, J = 7.2, 2H), 7.45 – 7.36 (m, 2H), 7.34 (dd, J = 8.4, 7.2 Hz, 2H), 6.86 (d, J = 8.4, 2H), 5.51 (t, J = 8.4 Hz, 1H), 4.26 (t, J = 3.6 Hz, 2H), 2.26 (t, J = 6.1 Hz, 2H), 1.73 (dt, J = 8.4, 3.6 Hz, 2H), 1.24 – 1.15 (m, 8H), 0.86 (t, J = 6.9 Hz, 3H). **^{13}C NMR (101 MHz, CDCl$_3$)** δ 140.25, 140.04, 135.49, 132.02, 131.42, 130.45, 129.95, 128.18, 122.15, 120.37, 39.49, 31.86, 28.91, 28.23, 22.84, 14.30, 13.20; **IR (neat)**: ν_{max} = 2925, 2136, 931, 834, 729, 702 cm^{-1}; **HRMS m/z (EI)** calc. for $C_{21}H_{27}BrSi$ [M$^+$] = 386.1065, found 386.1065; **R_f** 0.46 (Hexane).

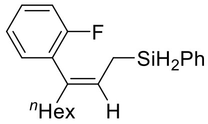

(Z)-(3-(2-fluorophenyl)non-2-en-1-yl)(phenyl)silane, **3k** (89 mg, 68%): colorless liquid; **^{1}H NMR (400 MHz, CDCl$_3$)** δ 7.50 (d, J = 6.3 Hz, 2H), 7.40 (t, J = 7.3 Hz, 1H), 7.34 (dd, J = 7.3, 6.3 Hz, 2H), 7.26 – 7.20 (m, 1H), 7.09 – 7.02 (m, 1H), 6.95 (dd, J = 7.7, 7.4, 1H), 5.64 (t, J = 8.1 Hz, 1H), 4.26 (t, J = 3.7 Hz, 2H), 2.31 (t, J = 6.7 Hz, 1H), 1.62 (dt, J = 8.1, 3.7 Hz, 1H), 1.34 – 1.18 (m, 8H), 0.87 (t, J = 6.8 Hz, 3H); **^{13}C NMR (101 MHz, CDCl$_3$)** δ 159.87 (d, $J_{C–F}$ = 244.9 Hz), 135.45, 132.21, 131.30 (d, $J_{C–F}$ = 4.5 Hz), 129.85, 128.49 (d, $J_{C–F}$ = 8.0 Hz), 128.49 (d, $J_{C–F}$ = 17.1 Hz), 128.13, 123.95, 123.90, 115.74 (d, $J_{C–F}$ = 22.7 Hz), 38.76 (d, $J_{C–F}$ = 1.3 Hz), 31.91, 28.97, 28.25, 22.84, 14.31, 13.37 (one carbon signal is missing due to the overlap of aromatic carbon peaks); **IR (neat)**: ν_{max} = 2926, 2136, 859, 841, 756, 700 cm^{-1}; **HRMS m/z (EI)** calc. for $C_{21}H_{27}FSi$ [M$^+$] = 326.1866, found 326.1866; $\boldsymbol{R_f}$ 0.47 (Hexane).

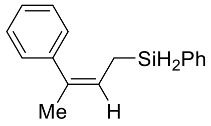

(Z)-phenyl(3-phenylbut-2-en-1-yl)silane, (Z)-**3l** (61 mg, 64%): colorless liquid; **^{1}H NMR (600 MHz, CDCl$_3$)** δ 7.41 (d, J = 7.9 Hz, 2H), 7.30 (t, J = 7.4 Hz, 1H), 7.24 (dd, J = 7.4, 7.0 Hz, 2H), 7.21 (dd, J = 7.9, 7.4 Hz, 2H), 7.13 (t, J = 7.4 Hz, 1H), 7.02 (d, J = 7.3 Hz, 2H), 5.45 (t, J = 8.2 Hz, 1H), 4.20 (t, J = 3.7 Hz, 2H), 1.92 (s, 3H), 1.73 (dt, J = 8.2, 3.7 Hz, 2H); **^{13}C NMR (151 MHz, CDCl$_3$)** δ 142.19, 136.30, 135.46, 132.21, 129.86, 128.37, 128.19, 128.15, 126.59, 121.87, 25.96, 13.17; **IR (neat)**: ν_{max} = 2964, 2133, 932, 864, 836, 697 cm^{-1}; **HRMS** m/z (EI) calc. for $C_{16}H_{18}Si$ [M$^+$] 238.1178, found 238.1178; $\boldsymbol{R_f}$ 0.48 (Hexane).

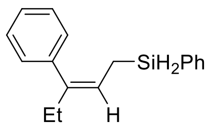

(*Z*)-phenyl(3-phenylpent-2-en-1-yl)silane, (*Z*)-**3m** (68 mg, 67%): colorless liquid; **^{1}H NMR (600 MHz, $CDCl_3$)** δ 7.56 (d, *J* = 7.0 Hz, 2H), 7.45 (t, *J* = 7.4 Hz, 1H), 7.39 (dd, *J* = 7.4, 7.0 Hz, 2H), 7.35 (dd, *J* = 7.4, 7.2 Hz, 2H), 7.28 (t, *J* = 7.2 Hz, 1H), 7.09 (d, *J* = 7.4 Hz, 2H), 5.57 (t, *J* = 8.4 Hz, 1H), 4.33 (t, *J* = 3.7 Hz, 2H), 2.38 (q, *J* = 7.4 Hz, 2H), 1.82 (dt, *J* = 8.4, 3.7 Hz, 2H), 1.00 (t, *J* = 7.4 Hz, 3H); **^{13}C NMR (151 MHz, $CDCl_3$)** δ 142.86, 141.49, 135.49, 132.30, 129.83, 128.70, 128.27, 128.12, 126.50, 120.27, 32.54, 13.43, 12.96; **IR (neat)**: ν_{max} = 2963, 2134, 932, 858, 840, 699 cm^{-1}; **HRMS** m/z (EI) calc. for $C_{17}H_{20}Si$ [M^+] 252.1334, found 252.1334; ***R_f*** 0.50 (Hexane).

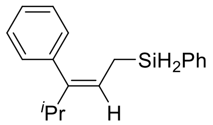

(*Z*)-(4-methyl-3-phenylpent-2-en-1-yl)(phenyl)silane, **3n** (74 mg, 70%): colorless liquid; **^{1}H NMR (600 MHz, $CDCl_3$)** δ 7.48 (d, *J* = 7.2 Hz, 2H), 7.39 (t, *J* = 7.6 Hz, 1H), 7.33 (dd, *J* = 7.6, 7.2 Hz, 2H), 7.27 (dd, *J* = 7.4, 7.1 Hz, 2H), 7.21 (t, *J* = 7.4 Hz, 1H), 6.93 (d, *J* = 7.1 Hz, 2H), 5.48 (t, *J* = 8.3 Hz, 1H), 4.23 (t, *J* = 3.4 Hz, 2H), 2.51 (hept, *J* = 6.8 Hz, 1H), 1.64 (dt, *J* = 8.3, 3.4 Hz, 2H), 0.97 (d, *J* = 6.8 Hz, 6H); **^{13}C NMR (151 MHz, $CDCl_3$)** δ 147.48, 141.10, 135.54, 132.38, 129.81, 129.30, 128.08, 128.05, 126.37, 119.11, 36.39, 22.13, 12.99; **IR (neat)**: ν_{max} = 2958, 2924, 2137, 932, 842, 700 cm^{-1}; **HRMS** m/z (EI) calc. for $C_{18}H_{22}Si$ [M^+] 266.1491, found 266.1491; ***R_f*** 0.53 (Hexane).

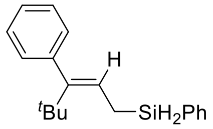

(*E*)-(4,4-dimethyl-3-phenylpent-2-en-1-yl)(phenyl)silane, (*E*)-**3o** (95 mg, 85%): colorless liquid; **^{1}H NMR (600 MHz, $CDCl_3$)** δ 7.61 (d, *J* = 7.1 Hz, 2H), 7.42 (t, *J* = 7.2 Hz, 1H), 7.38 (dd, *J* = 7.2, 7.1 Hz, 2H), 7.24 (dd, *J* = 7.4, 7.3 Hz, 2H), 7.19 (t, *J* = 7.4 Hz, 1H), 7.04 (d, *J* = 7.3 Hz, 2H), 5.26 (t, *J* = 9.2 Hz, 1H), 4.40 (t, *J* = 3.7 Hz, 2H), 2.19 (dt, *J* = 9.2, 3.7 Hz, 2H), 1.14 (s, 9H); **^{13}C NMR (151 MHz, $CDCl_3$)** δ 149.02, 146.98, 135.54, 132.19, 129.98, 129.05, 128.20, 127.38, 125.79, 125.04, 35.53, 31.69, 15.19; **IR (neat)**: ν_{max} = 2961, 2137, 932, 872, 836, 701 cm^{-1}; **HRMS** m/z (EI) calc. for $C_{19}H_{24}Si$ [M^+] 280.1647, found 280.1647; ***R_f*** 0.42 (Hexane).

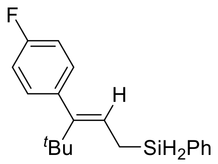

(*E*)-(3-(4-fluorophenyl)-4,4-dimethylpent-2-en-1-yl)(phenyl), **3p** (79 mg, 66%): colorless liquid; **^{1}H NMR (400 MHz, $CDCl_3$)** δ 7.60 (d, *J* = 6.1 Hz, 2H), 7.42 – 7.35 (m, 3H), 6.98 (dd, *J* = 8.8 Hz, $J_{H\text{-}F}$ = 5.6 Hz, 2H), 6.92 (dd, *J* = 8.8 Hz, $J_{H\text{-}F}$ = 8.7 Hz, 2H), 5.23 (t, *J* = 9.2 Hz, 1H), 4.38 (t, *J* = 3.7 Hz, 2H), 2.16 (dt, *J* = 9.2, 3.7 Hz, 2H), 1.11 (s, 9H); **^{13}C NMR (101 MHz, $CDCl_3$)** δ 161.37 (d, $J_{C\text{–}F}$ = 243.5 Hz), 147.95, 142.80 (d, $J_{C\text{–}F}$ = 3.3 Hz), 135.52, 132.07, 130.41 (d, $J_{C\text{–}F}$ = 7.7 Hz), 130.03, 128.22, 125.67, 114.10 (d, $J_{C\text{–}F}$ = 21.0 Hz), 35.56, 31.63, 15.20; **IR (neat)**: ν_{max} = 2139, 1505, 1219, 872, 835, 701 cm^{-1}; **HRMS m/z (EI)** calc. for $C_{19}H_{23}FSi$ [M^+] = 298.1553, found 298.1553; ***R_f*** 0.44 (Hexane)

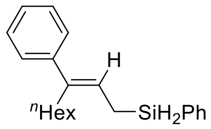

(*E*)-phenyl(3-phenylnon-2-en-1-yl)silane, (*E*)-**3a** (73 mg, 59%): colorless liquid; **^{1}H NMR (600 MHz, CDCl$_3$)** δ 7.61 (d, *J* = 6.4 Hz, 1H), 7.42 (t, *J* = 7.3 Hz, 1H), 7.38 (dd, *J* = 7.3, 6.4 Hz, 2H), 7.30 – 7.28 (m, 3H), 7.23 – 7.19 (m, 1H), 5.74 (t, *J* = 8.5 Hz, 1H), 4.38 (t, *J* = 3.6 Hz, 2H), 2.40 (d, *J* = 7.5 Hz, 1H), 2.05 (dt, *J* = 8.5, 3.6 Hz, 2H), 1.32 – 1.16 (m, 4H), 0.86 (t, *J* = 7.0 Hz, 3H); **^{13}C NMR (151 MHz, CDCl$_3$)** δ 143.59, 140.10, 135.50, 132.24, 129.98, 128.33, 128.24, 126.50, 126.42, 123.30, 31.90, 29.74, 29.64, 28.72, 22.83, 14.28, 13.56; **IR (neat)**: ν_{max} = 2926, 2137, 932, 867, 838, 698 cm^{-1}; **HRMS** m/z (EI) calc. for $C_{21}H_{28}Si$ [M$^+$] 308.1960, found 308.1960; $\boldsymbol{R_f}$ 0.68 (Hexane).

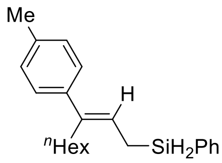

(*E*)-phenyl(3-(p-tolyl)non-2-en-1-yl)silane, (*E*)-**3b** (41 mg, 32%): colorless liquid; **^{1}H NMR (600 MHz, CDCl$_3$)** δ 7.60 (d, *J* = 7.1 Hz, 2H), 7.41 (t, *J* = 7.3 Hz, 1H), 7.37 (dd, *J* = 7.3, 7.1 Hz, 2H), 7.18 (d, *J* = 7.7 Hz, 2H), 7.10 (d, *J* = 7.8 Hz, 2H), 5.70 (t, *J* = 8.5 Hz, 1H), 4.36 (t, *J* = 3.7 Hz, 2H), 2.37 (t, *J* = 7.0 Hz, 2H), 2.33 (s, 3H), 2.03 (dt, *J* = 8.5, 3.7 Hz, 2H), 1.26 – 1.19 (m, 8H), 0.85 (t, *J* = 7.0 Hz, 3H); **^{13}C NMR (151 MHz, CDCl$_3$)** δ 140.67, 139.88, 136.10, 135.50, 132.32, 129.95, 129.04, 128.21, 126.27, 122.49, 31.91, 29.72, 29.67, 28.73, 22.84, 21.23, 14.29, 13.47; **IR (neat)**: ν_{max} = 2954, 2925, 2138, 933, 840, 700 cm^{-1}; **HRMS** m/z (EI) calc. for $C_{22}H_{30}Si$ [M$^+$] 322.2117, found 322.2117; $\boldsymbol{R_f}$ 0.43 (Hexane).

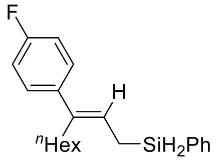

(*E*)-(3-(4-fluorophenyl)non-2-en-1-yl)(phenyl)silane, (*E*)-**3h** (40 mg, 31%): colorless liquid; **^{1}H NMR (600 MHz, CDCl$_3$)** δ 7.59 (d, *J* = 7.5 Hz, 2H), 7.42 (t, *J* = 7.4 Hz, 1H), 7.37 (dd, *J* = 7.5, 7.4 Hz, 2H), 7.22 (dd, *J* = 8.8 Hz, $J_{H\text{-}F}$ = 5.4 Hz, 2H), 6.96 (dd, *J* = 8.8 Hz, $J_{H\text{-}F}$ = 8.7 Hz, 2H), 5.66 (t, *J* = 8.5 Hz, 1H), 4.36 (t, *J* = 3.7 Hz, 2H), 2.36 (t, *J* = 7.1 Hz, 2H), 2.02 (dt, *J* = 8.5, 3.7 Hz, 2H), 1.25 – 1.20 (m, 8H), 0.85 (t, *J* = 6.9 Hz, 3H); **^{13}C NMR (151 MHz, CDCl$_3$)** δ 161.86 (d, $J_{C\text{-}F}$ = 244.5 Hz), 139.63 (d, $J_{C\text{-}F}$ = 3.2 Hz), 139.15, 135.48, 132.14, 130.03, 128.26, 127.87 (d, $J_{C\text{-}F}$ = 7.8 Hz), 123.32, 115.06 (d, $J_{C\text{-}F}$ = 21.4 Hz), 31.89, 29.86, 29.56, 28.60, 22.82, 14.27, 13.51; **IR (neat)**: ν_{max} = 2927, 1508, 1231, 867, 834, 700 cm^{-1}; **HRMS** m/z (EI) calc. for $C_{21}H_{27}FSi$ [M$^+$] 326.1866, found 326.1866; $\boldsymbol{R_f}$ 0.45 (Hexane).

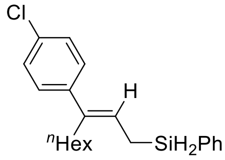

(*E*)-(3-(4-chlorophenyl)non-2-en-1-yl)(phenyl)silane, (*E*)-**3i** (68 mg, 50%): colorless liquid; **^{1}H NMR (600 MHz, CDCl$_3$)** δ 7.59 (d, *J* = 7.1 Hz, 2H), 7.42 (t, *J* = 7.4 Hz, 1H), 7.37 (dd, *J* = 7.4, 7.1 Hz, 2H), 7.24 (d, *J* = 8.4 Hz, 2H), 7.19 (d, *J* = 8.5 Hz, 2H), 5.71 (t, *J* = 8.5 Hz, 1H), 4.36 (t, *J* = 3.7 Hz, 2H), 2.36 (t, *J* = 7.0 Hz, 2H), 2.03 (dt, *J* = 8.5, 3.7 Hz, 2H), 1.26 – 1.19 (m, 8H), 0.85 (t, *J* = 7.0 Hz, 3H); **^{13}C NMR (151 MHz, CDCl$_3$)** δ 142.00, 139.00, 135.48, 132.16, 132.04, 130.06, 128.44, 128.27, 127.70, 123.98, 31.88,

29.61, 29.56, 28.60, 22.81, 14.27, 13.65; **IR (neat)**: ν_{max} = 2927, 2139, 1490, 932, 827, 701 cm^{-1}; **HRMS** m/z (EI) calc. for $C_{21}H_{27}ClSi$ [M$^+$] 342.1571, found 342.1571; ***R*$_f$** 0.50 (Hexane).

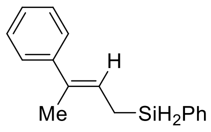

(*E*)-phenyl(3-phenylbut-2-en-1-yl)silane, (*E*)-**3l** (30 mg, 31%): colorless liquid; **^{1}H NMR (600 MHz, CDCl$_3$)** δ 7.60 (d, *J* = 7.0 Hz, 2H), 7.41 (t, *J* = 7.2 Hz, 1H), 7.36 (dd, *J* = 7.2, 7.0 Hz, 2H), 7.33 (d, *J* = 7.7 Hz, 2H), 7.30 (dd, *J* = 7.7, 7.3 Hz, 2H), 7.21 (t, *J* = 7.3 Hz, 1H), 5.90 (t, *J* = 8.6 Hz, 1H), 4.37 (t, *J* = 3.6 Hz, 2H), 2.06 (dt, *J* = 8.6, 3.6 Hz, 2H), 1.93 (s, 3H); **^{13}C NMR (151 MHz, CDCl$_3$)** δ 144.17, 135.48, 134.33, 132.25, 129.98, 128.36, 128.23, 126.54, 125.70, 123.23, 15.79, 13.89; **IR (neat)**: ν_{max} = 2923, 2853, 2139, 933, 869, 699 cm^{-1}; **HRMS** m/z (EI) calc. for $C_{16}H_{18}Si$ [M$^+$] 238.1178, found 238.1178; ***R*$_f$** 0.35 (Hexane).

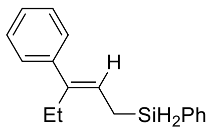

(*E*)-phenyl(3-phenylpent-2-en-1-yl)silane, (*E*)-**3m** (38 mg, 38%): colorless liquid; **^{1}H NMR (600 MHz, CDCl$_3$)** δ 7.61 (d, *J* = 6.9 Hz, 2H), 7.41 (t, *J* = 7.2 Hz, 1H), 7.37 (dd, *J* = 7.2, 6.9 Hz, 2H), 7.32 – 7.27 (m, 4H), 7.23 – 7.19 (m, 1H), 5.74 (t, *J* = 8.5 Hz, 1H), 4.37 (t, *J* = 3.7 Hz, 2H), 2.44 (q, *J* = 7.6 Hz, 2H), 2.05 (dt, *J* = 8.5, 3.7 Hz, 2H), 0.91 (t, *J* = 7.6 Hz, 3H); **^{13}C NMR (151 MHz, CDCl$_3$)** δ 143.18, 141.24, 135.50, 132.22, 130.00, 128.36, 128.24, 126.54, 126.40, 122.79, 22.75, 13.47, 13.45; **IR (neat)**: ν_{max} = 2966, 2138, 933, 868, 839, 698 cm^{-1}; **HRMS** m/z (EI) calc. for $C_{17}H_{20}Si$ [M$^+$] 252.1334, found 252.1334; ***R*$_f$** 0.33 (Hexane).

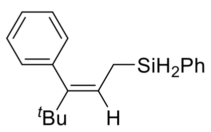

(*Z*)-(4,4-dimethyl-3-phenylpent-2-en-1-yl)(phenyl)silane, (*Z*)-**3o** (49 mg, 44%): colorless liquid; **^{1}H NMR (600 MHz, CDCl$_3$)** δ 7.48 (d, *J* = 7.2 Hz, 2H), 7.40 (t, *J* = 7.5 Hz, 1H), 7.34 (dd, *J* = 7.5, 7.2 Hz, 2H), 7.26 (dd, *J* = 7.3, 7.0 Hz, 2H), 7.22 (t, *J* = 7.0 Hz, 1H), 6.84 (d, *J* = 7.3 Hz, 2H), 5.61 (t, *J* = 8.3 Hz, 1H), 4.21 (t, *J* = 3.6 Hz, 2H), 1.47 (dt, *J* = 8.3, 3.6 Hz, 2H), 1.04 (s, 9H); **^{13}C NMR (151 MHz, CDCl$_3$)** δ 150.27, 140.54, 135.58, 132.48, 130.29, 129.79, 128.05, 127.73, 126.18, 118.74, 36.22, 29.99, 13.64; **IR (neat)**: ν_{max} = 2963, 2135, 932, 860, 842, 703 cm^{-1}; **HRMS** m/z (EI) calc. for $C_{19}H_{24}Si$ [M$^+$] 280.1647, found 280.1647; ***R*$_f$** 0.49 (Hexane).

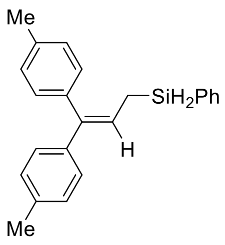

(3,3-di-p-tolylallyl)(phenyl)silane, **3q** (122 mg, 93%): yellow liquid; **^{1}H NMR (600 MHz, CDCl$_3$)** δ 7.58 (d, *J* = 7.1 Hz, 2H), 7.45 (t, *J* = 7.5 Hz, 1H), 7.39 (dd, *J* = 7.5, 7.1 Hz, 2H), 7.20 (d, *J* = 7.7 Hz, 2H), 7.13 (d, *J* = 8.1 Hz, 2H), 7.10 (d, *J* = 8.1 Hz, 2H), 7.05 (d, *J* = 7.7 Hz, 2H), 6.20 (t, *J* = 8.6 Hz, 1H), 4.40 (t, *J* = 3.6 Hz, 2H), 2.43 (s, 3H), 2.37 (s, 3H), 2.05 (dt, *J* = 8.6, 3.6 Hz, 2H); **^{13}C NMR (151 MHz, CDCl$_3$)** δ 141.11, 140.36, 137.27, 136.51, 136.46, 135.51, 131.99, 130.05, 129.94, 129.17, 128.96, 128.20, 127.04, 123.63, 21.45, 21.23, 14.64; **IR (neat)**: ν_{max} = 2133, 931, 859, 839, 815, 700 cm^{-1}; **HRMS** m/z (EI) calc. for $C_{23}H_{24}Si$ [M$^+$] 328.1647, found 328.1649; ***R*$_f$** 0.24 (Hexane).

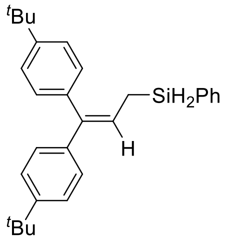

(3,3-bis(4-(*tert*-butyl)phenyl)allyl)(phenyl)silane, **3r** (97 mg, 59%): colorless liquid; **^{1}H NMR (400 MHz, CDCl$_3$)** δ 7.51 (d, *J* = 7.2, 2H), 7.38 (t, *J* = 7.3 Hz, 1H), 7.35-7.30 (m, 4H), 7.25 (d, *J* = 8.5 Hz, 2H), 7.11 (d, *J* = 8.6 Hz, 2H), 7.02 (d, *J* = 8.5 Hz, 2H), 6.15 (t, *J* = 8.6 Hz, 1H), 4.33 (t, *J* = 3.6 Hz, 2H), 1.98 (dt, *J* = 8.7, 3.6 Hz, 2H), 1.34 (s, 9H), 1.29 (s, 9H).; **^{13}C NMR (101 MHz, CDCl$_3$)** δ 149.69, 149.65, 140.96, 140.18, 137.08, 135.50, 132.08, 129.92, 129.75, 128.20, 126.71, 125.28, 125.14, 123.79, 34.74, 34.61, 31.64, 31.54, 14.62; **IR (neat)**: ν_{max} = 2135, 1116, 932, 862, 832, 700 cm^{-1}; **HRMS m/z (EI)** calc. for $C_{29}H_{36}Si$ [M$^+$] = 412.2586, found 412.2586; ***R$_f$*** 0.40 (Hexane).

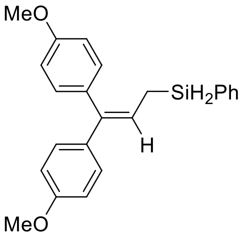

(3,3-bis(4-methoxyphenyl)allyl)(phenyl)silane, **3s** (118 mg, 82%): brown liquid; **^{1}H NMR (600 MHz, CDCl$_3$)** δ 7.55 (d, *J* = 7.4 Hz, 2H), 7.42 (t, *J* = 7.6 Hz, 1H), 7.37 (dd, *J* = 7.6, 7.4 Hz, 2H), 7.12 (d, *J* = 9.0 Hz, 2H), 7.04 (d, *J* = 8.8 Hz, 2H), 6.90 (d, *J* = 8.8 Hz, 2H), 6.80 (d, *J* = 9.0 Hz, 2H), 6.08 (t, *J* = 8.6 Hz, 1H), 4.36 (t, *J* = 3.6 Hz, 2H), 3.85 (s, 3H), 3.80 (s, 3H), 2.01 (dt, *J* = 8.6, 3.6 Hz, 2H); **^{13}C NMR (151 MHz, CDCl$_3$)** δ 158.71, 158.60, 140.37, 136.05, 135.48, 132.61, 132.00, 131.23, 129.92, 128.21, 128.19, 122.68, 113.84, 113.62, 55.43, 55.37, 14.52; **IR (neat)**: ν_{max} = 2134, 1508, 1241, 1032, 828, 701 cm^{-1}; **HRMS** m/z (EI) calc. for $C_{23}H_{24}O_2Si$ [M$^+$] 360.1546, found 360.1546; ***R$_f$*** 0.44 (Hexane).

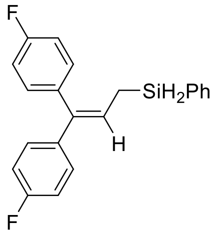

(3,3-bis(4-fluorophenyl)allyl)(phenyl)silane, **3t** (78 mg, 58%): yellow liquid; **^{1}H NMR (600 MHz, CDCl$_3$)** δ 7.52 (d, *J* = 7.6 Hz, 2H), 7.43 (t, *J* = 7.4 Hz, 1H), 7.36 (t, *J* = 7.4 Hz, 2H), 7.11 (dd, *J* = 8.5 Hz, $J_{H\text{-}F}$ = 5.5 Hz, 2H), 7.06 – 7.00 (m, 4H), 6.94 (dd, $J_{H\text{-}F}$ =8.7 Hz, *J* =8.5 Hz, 2H), 6.14 (t, *J* = 8.6 Hz, 1H), 4.35 (t, *J* = 3.6 Hz, 2H), 1.98 (dt, *J* = 8.6, 3.6 Hz, 2H); **^{13}C NMR (151 MHz, CDCl$_3$)** δ 162.91 (d, $J_{C\text{-}F}$ = 8.6 Hz), 161.28 (d, $J_{C\text{-}F}$ = 8.7 Hz), 139.32, 138.89 (d, $J_{C\text{-}F}$ = 3.2 Hz), 135.73 (d, $J_{C\text{-}F}$ = 3.3 Hz), 135.47, 131.68 (d, $J_{C\text{-}F}$ = 7.9 Hz), 131.58, 130.11, 128.55 (d, $J_{C\text{-}F}$ = 7.8 Hz), 128.29, 125.04, 115.55 (d, $J_{C\text{-}F}$ = 21.2 Hz), 115.14 (d, $J_{C\text{-}F}$ = 21.3 Hz), 14.80; **IR (neat)**: ν_{max} = 2140, 1507, 1223, 864, 835, 703 cm^{-1}; **HRMS** m/z (EI) calc. for $C_{21}H_{18}F_2Si$ [M$^+$] 336.1146, found 336.1146; ***R$_f$*** 0.30 (Hexane).

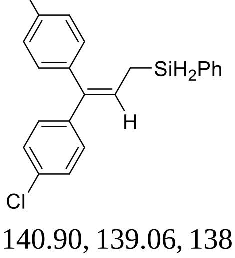

(3,3-bis(4-chlorophenyl)allyl)(phenyl)silane, **3u** (118 mg, 80%): colorless liquid; **^{1}H NMR (600 MHz, CDCl$_3$)** δ 7.52 (d, *J* = 7.4 Hz, 2H), 7.43 (t, *J* = 7.5 Hz, 1H), 7.37 (dd, *J* = 7.5, 7.4 Hz, 2H), 7.32 (d, *J* = 8.4 Hz, 2H), 7.22 (d, *J* = 8.7 Hz, 2H), 7.06 (d, *J* = 8.7 Hz, 2H), 6.97 (d, *J* = 8.4 Hz, 2H), 6.20 (t, *J* = 8.7 Hz, 1H), 4.36 (t, *J* = 3.5 Hz, 2H), 1.98 (dt, *J* = 8.7, 3.5 Hz, 2H); **^{13}C NMR (151 MHz, CDCl$_3$)** δ 140.90, 139.06, 138.00, 135.46, 133.19, 132.85, 131.47, 131.39, 130.16, 128.88, 128.50, 128.31, 128.27,

126.01, 15.00; **IR (neat)**: ν_{max} = 2136, 1489, 1089, 859, 824, 700 cm^{-1}; **HRMS** m/z (EI) calc. for $C_{21}H_{18}Cl_2Si$ [M^+] 368.0555, found 368.0557; $\boldsymbol{R_f}$ 0.33 (Hexane).

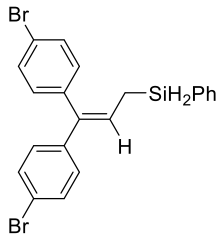

(3,3-bis(4-bromophenyl)allyl)(phenyl)silane, **3v** (87 mg, 48%): colorless liquid; **^{1}H NMR (400 MHz, $CDCl_3$)** δ 7.49 (d, *J* = 7.1 Hz, 2H), 7.45 (dd, *J* = 7.1, 7.4 Hz, 2H), 7.41 (t, *J* = 7.4 Hz, 1H), 7.37 – 7.33 (m, 4H), 6.99 (d, *J* = 8.6 Hz, 2H), 6.90 (d, *J* = 8.4 Hz, 2H), 6.19 (t, *J* = 8.6 Hz, 1H), 4.33 (t, *J* = 3.5 Hz, 2H), 1.96 (dt, *J* = 8.6, 3.5 Hz, 2H).; **^{13}C NMR (101 MHz, $CDCl_3$)** δ 141.28, 139.11, 138.41, 135.47, 131.86, 131.83, 131.46, 131.37, 130.18, 128.64, 128.33, 126.12, 121.39, 121.03, 15.06; **IR (neat)**: ν_{max} = 2139, 1486, 1072, 860, 843, 824 cm^{-1}; **HRMS m/z (EI)** calc. for $C_{21}H_{18}Br_2Si$ [M^+] = 455.9545, found 455.9545 $\boldsymbol{R_f}$ 0.45 (Hexane).

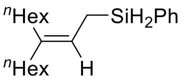

(3-hexylnon-2-en-1-yl)(phenyl)silane, **3w** (17 mg, 53%): colorless liquid; **^{1}H NMR (400 MHz, $CDCl_3$)** δ 7.57 (d, *J* = 6.8 Hz, 2H), 7.41 – 7.32 (m, 3H), 5.18 (t, *J* = 8.3 Hz, 1H), 4.27 (t, *J* = 3.7 Hz, 2H), 3.70 (t, *J* = 5.6 Hz, 2H), 1.99 – 1.90 (m, 5H), 1.81 (dt, *J* = 8.3, 3.7 Hz, 2H), 1.70 (t, *J* = 5.8 Hz, 2H), 1.36 – 1.19 (m, 10H), 0.88 (t, *J* = 6.7 Hz, 6H); **^{13}C NMR (101 MHz, $CDCl_3$)** δ 139.85, 135.46, 132.76, 129.78, 128.11, 118.47, 63.05, 37.15, 32.06, 32.03, 30.08, 29.96, 29.81, 29.28, 28.51, 28.42, 22.89, 14.34, 11.90; **IR (neat)**: ν_{max} = 2926, 2137, 1118, 934, 843, 699 cm^{-1}; **LRMS** m/z (EI) calc. for $C_{21}H_{36}Si$ [M+] = 316.3, found 316.3; $\boldsymbol{R_f}$ 0.71 (Hexane).

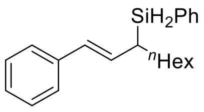

(*E*)-phenyl(1-phenylnon-1-en-3-yl)silane, **3x** (22 mg, 70%): colorless liquid; **^{1}H NMR (400 MHz, $CDCl_3$)** δ 7.56 (d, *J* = 7.1 Hz, 2H), 7.43 – 7.38 (m, 2H), 7.36 – 7.31 (m, 2H), 7.29 – 7.24 (m, 3H), 7.17 (t, *J* = 9.6 Hz, 1H), 6.26 (d, *J* = 15.9 Hz, 1H), 6.13 (dd, *J* = 15.9, 9.1 Hz, 1H), 4.30 (d, *J* = 2.9 Hz, 2H), 2.18 (dtt, *J* = 9.1, 6.0, 2.9 Hz, 1H), 1.32 – 1.20 (m, 10H), 0.86 (t, *J* = 7.2 Hz, 6H); **^{13}C NMR (101 MHz, $CDCl_3$)** δ 138.27, 136.00, 132.21, 131.46, 129.95, 128.87, 128.68, 128.13, 126.77, 125.94, 31.94, 30.85, 29.89, 29.52, 29.34, 22.86, 14.28; **IR (neat)**: ν_{max} = 2924, 2134, 929, 835, 733, 695 cm^{-1}; **LRMS** m/z (EI) calc. for $C_{21}H_{28}Si$ [M+] = 308.2, found 308.2; $\boldsymbol{R_f}$ 0.32 (Hexane).

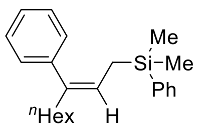

(*Z*)-dimethyl(phenyl)(3-phenylnon-2-en-1-yl)silane, **4** (38 mg, 95%): colorless liquid; **^{1}H NMR (400 MHz, $CDCl_3$)** δ 7.44 (d, *J* = 6.2 Hz, 2H), 7.38 – 7.30 (m, 3H), 7.26 (dd, *J* = 7.6, 7.3 Hz, 2H), 7.19 (t, *J* = 7.3 Hz, 1H), 6.99 (d, *J* = 7.6 Hz, 2H), 5.44 (t, *J* = 8.3 Hz, 1H), 2.28 (t, *J* = 5.6 Hz, 2H), 1.61 (d, *J* = 8.3 Hz, 2H), 1.27 – 1.21 (m, 8H), 0.86 (t, *J* = 6.7 Hz, 3H), 0.23 (s, 6H); **^{13}C NMR (101 MHz, $CDCl_3$)** δ 141.60, 139.99, 138.94, 133.65, 128.89, 128.70, 127.94, 127.67, 126.00, 121.87, 39.64, 31.72, 28.82, 28.24, 22.68, 18.26, 14.12, -3.09; **LRMS** m/z (EI) calc. for $C_{23}H_{32}Si$ [M+] = 336.2, found 336.2; $\boldsymbol{R_f}$ 0.27 (Hexane).

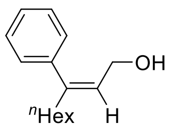

(*Z*)-3-phenylnon-2-en-1-ol, **5**[16] (18 mg, 82%): colorless liquid; **^{1}H NMR (400 MHz, $CDCl_3$)** δ 7.33 (dd, *J* = 7.4, 7.3 Hz, 2H), 7.27 (t, *J* = 7.3 Hz, 1H), 7.13 (d, *J* = 7.4 Hz, 2H), 5.68 (t, *J* = 6.9 Hz, 1H), 4.04 (d, *J* = 6.9 Hz, 2H), 2.38 (t, *J* = 7.1 Hz, 2H), 1.36 – 1.20 (m, 8H), 0.86 (t, *J* = 6.8 Hz, 3H); **^{13}C NMR (101 MHz, $CDCl_3$)** δ 145.27, 140.39, 128.33, 128.29, 127.23, 125.58, 60.50, 39.18, 31.84, 29.07, 28.08, 22.79, 14.27; **LRMS** m/z (EI) calc. for $C_{15}H_{22}O$ [M+] = 218.2, found 218.2; ***R_f*** 0.54 (Hex/EtOAc, 4/1).

References

(1) Z. Zhao, L. Racicot and G. K. Murphy, Fluorinative Rearrangements of Substituted Phenylallenes Mediated by (Difluoroiodo) toluene: Synthesis of α-(Difluoromethyl) styrenes, *Angew. Chem. Int. Ed.* 2017, **56**, 11620-11623.

(2) H. Clavier, K. L. Jeune, I. d. Riggi, A. Tenaglia and G. Buono, Highly selective cobalt-mediated [6+ 2] cycloaddition of cycloheptatriene and allenes, *Org. Lett.* 2011, **13**, 308-311.

(3) A. Köpfer and B. Breit, Rhodium-Catalyzed Hydroformylation of 1, 1-Disubstituted Allenes Employing the Self-Assembling 6-DPPon System, *Angew. Chem. Int. Ed.* 2015, **54**, 6913-6917.

(4) R. G. Parr and Y. Weitao, *Density-Functional Theory of Atoms and Molecules*, Oxford University Press, 1994.

(5) M. J. Frisch, G. W. Trucks, H. B. Schlegel, G. E. Scuseria, M. A. Robb, J. R. Cheeseman, G. Scalmani, V. Barone, G. A. Petersson, H. Nakatsuji, X. Li, M. Caricato, A. V. Marenich, J. Bloino, B. G. Janesko, R. Gomperts, B. Mennucci, H. P. Hratchian, J. V. Ortiz, A. F. Izmaylov, J. L. Sonnenberg, Williams, F. Ding, F. Lipparini, F. Egidi, J. Goings, B. Peng, A. Petrone, T. Henderson, D. Ranasinghe, V. G. Zakrzewski, J. Gao, N. Rega, G. Zheng, W. Liang, M. Hada, M. Ehara, K. Toyota, R. Fukuda, J. Hasegawa, M. Ishida, T. Nakajima, Y. Honda, O. Kitao, H. Nakai, T. Vreven, K. Throssell, J. A. Montgomery Jr., J. E. Peralta, F. Ogliaro, M. J. Bearpark, J. J. Heyd, E. N. Brothers, K. N. Kudin, V. N. Staroverov, T. A. Keith, R. Kobayashi, J. Normand, K. Raghavachari, A. P. Rendell, J. C. Burant, S. S. Iyengar, J. Tomasi, M. Cossi, J. M. Millam, M. Klene, C. Adamo, R. Cammi, J. W. Ochterski, R. L. Martin, K. Morokuma, O. Farkas, J. B. Foresman and D. J. Fox, Gaussian, Inc., Wallingford CT, 2016.

(6) S. Grimme, J. Antony, S. Ehrlich and H. Krieg, A Consistent and Accurate ab initio Parametrization of Density Functional Dispersion Correction (DFT-D) for the 94 Elements H-Pu. *J. Chem. Phys.* 2010, **132**, 154104.

(7) P. J. Hay and W. R. Wadt, Ab initio Effective Core Potentials for Molecular Calculations. Potentials for the Transition Metal Atoms Sc to Hg. *J. Chem. Phys.* 1985, **82**, 270–283.

(8) W. R. Wadt and P. J. Hay, Ab initio Effective Core Potentials for Molecular Calculations. Potentials for Main Group Elements Na to Bi. *J. Chem. Phys.* 1985, **82**, 284–298.

(9) P. J. Hay and W. R. Wadt, Ab initio Effective Core Potentials for Molecular Calculations. Potentials for K to Au Including the Outermost Core Orbitals. *J. Chem. Phys*. 1985, **82**, 299–310.

(10) A. Nicklass, M. Dolg, H. Stoll and H. Preuss, Ab initio Energy-Adjusted Pseudopotentials for the Noble Gases Ne through Xe: Calculation of Atomic Dipole and Quadrupole Polarizabilities. *J. Chem. Phys.* 1995, **102**, 8942–8952.

(11) C. Legault, CYLVIEW, 1.0 b, Université de Sherbrooke, 2009 (http://www.cylview.org).

(12) T. Lu and F. W. Chen, Multiwfn: A multifunctional wavefunction analyzer. *J. Comput. Chem.* 2012, **33**, 580–592.

(13) W. Humphrey, A. Dalke and K. Schulten, VMD: Visual molecular dynamics. *J. Mol. Graph. Model.* 1996, **14**, 33–38.

(14) A. Poater, F. Ragone, S. Giudice, C. Costabile, R. Dorta, S. P. Nolan and L. Cavallo, Thermodynamics of N-Heterocyclic Carbene Dimerization: The Balance of Sterics and Electronics. *Organometallics* 2008, **27**, 2679-2681.

(15) L. Falivene, Z. Cao, A. Petta, L. Serra, A. Poater, R. Oliva, V. Scarano and L. Cavallo, Towards the online computer-aided design of catalytic pockets. *Nat. Chem.* 2019, **11**, 872- 879.

(16) M. Lozanov and J. Montgomery, Nickel-catalyzed preparation of stereodefined allylic alcohols using silicon-tethered ynals, *Tetrahedron Lett.* 2001, **42**, 3259-3261.

NMR Spectra (^{1}H NMR, ^{13}C NMR and 1D NOESY)

(Z)-**3a**; ^{1}H NMR
(600 MHz, $CDCl_3$)

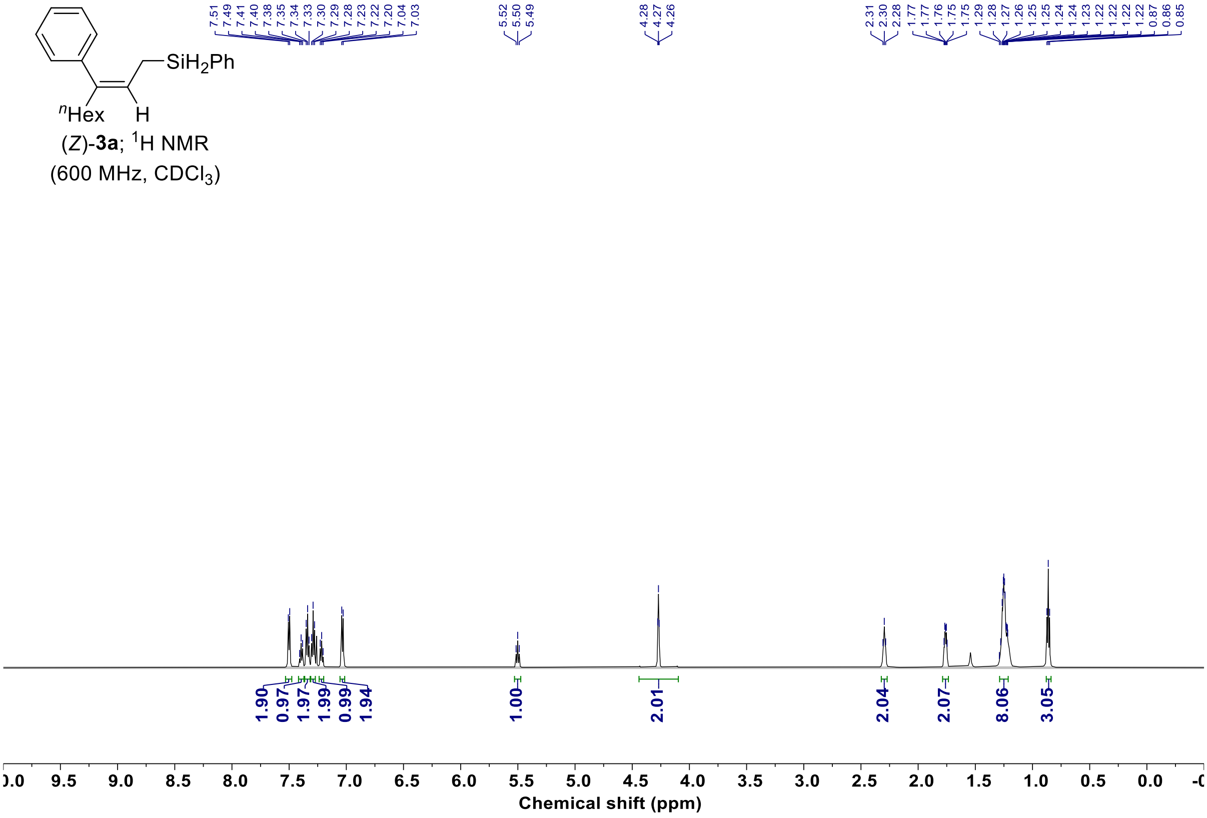

(Z)-**3a**; ^{13}C NMR
(151 MHz, $CDCl_3$)

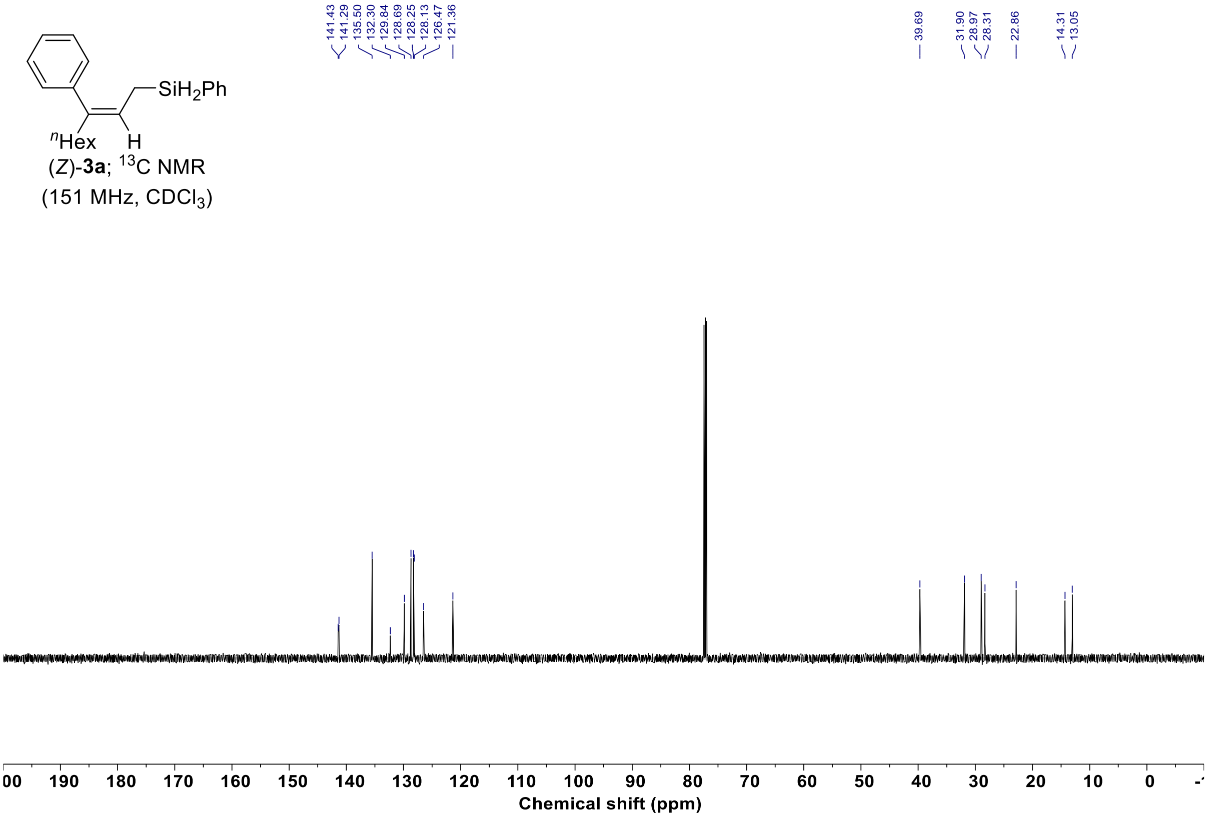

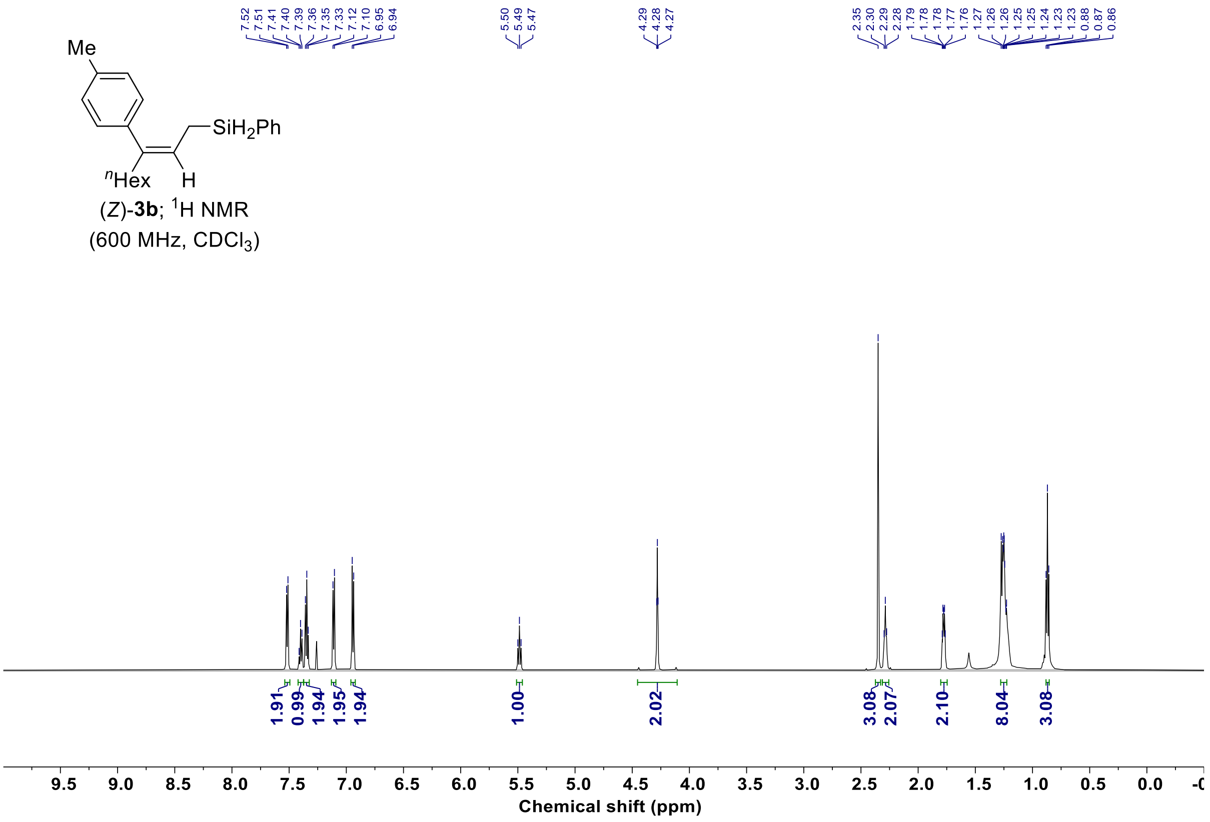

(*Z*)-**3b**; ^{1}H NMR
(600 MHz, $CDCl_3$)

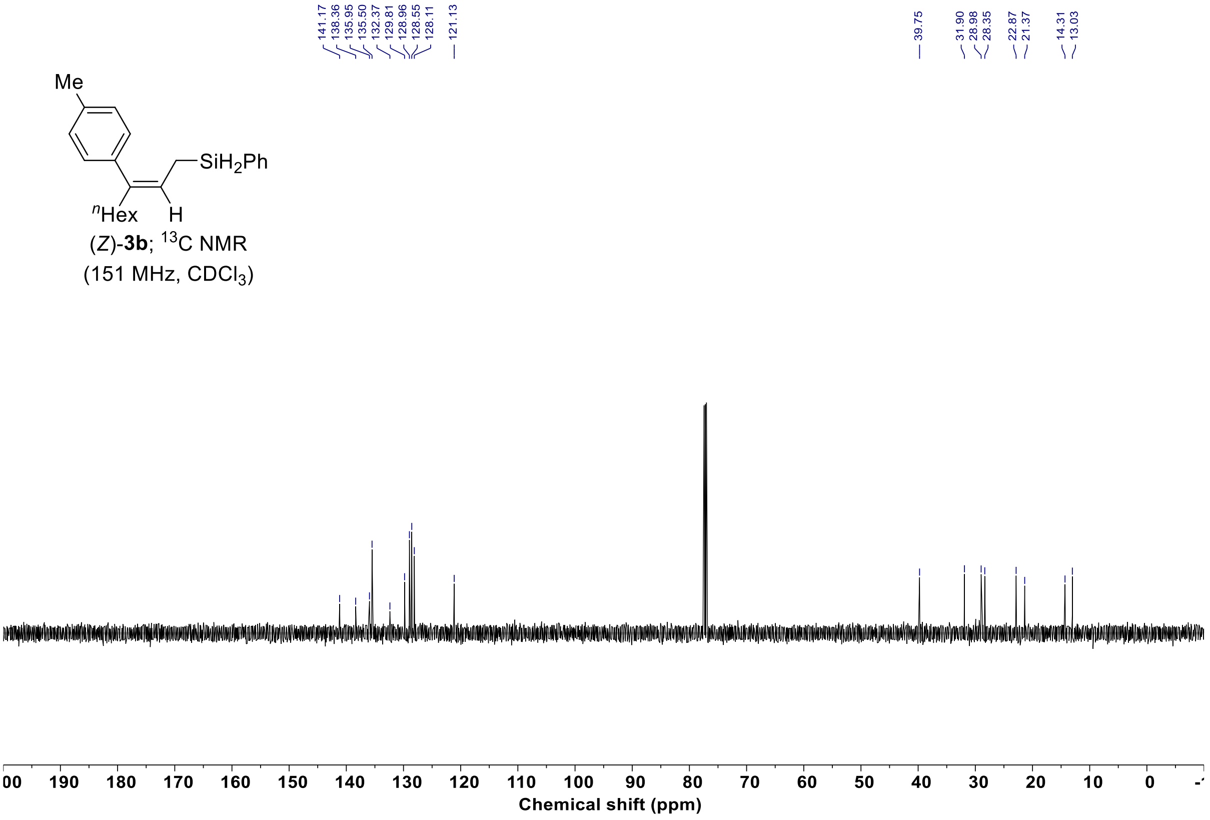

(*Z*)-**3b**; ^{13}C NMR
(151 MHz, $CDCl_3$)

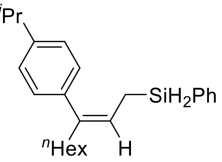

3c; ^{1}H NMR
(400 MHz, $CDCl_3$)

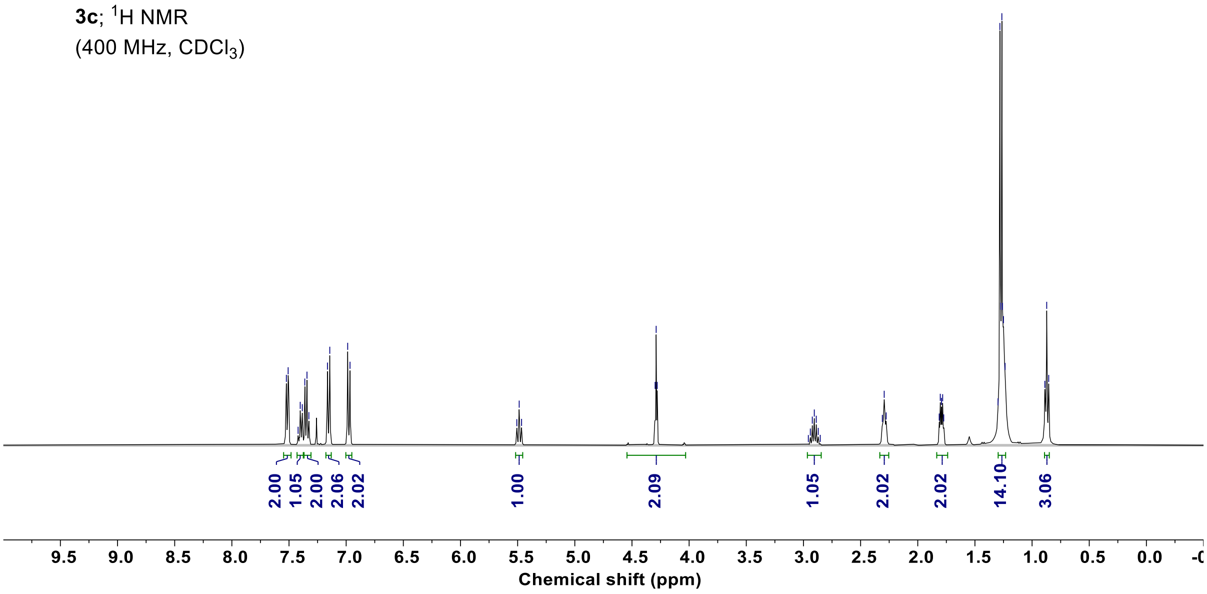

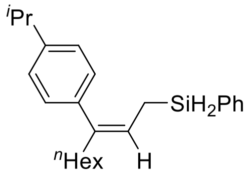

3c; ^{13}C NMR
(101 MHz, $CDCl_3$)

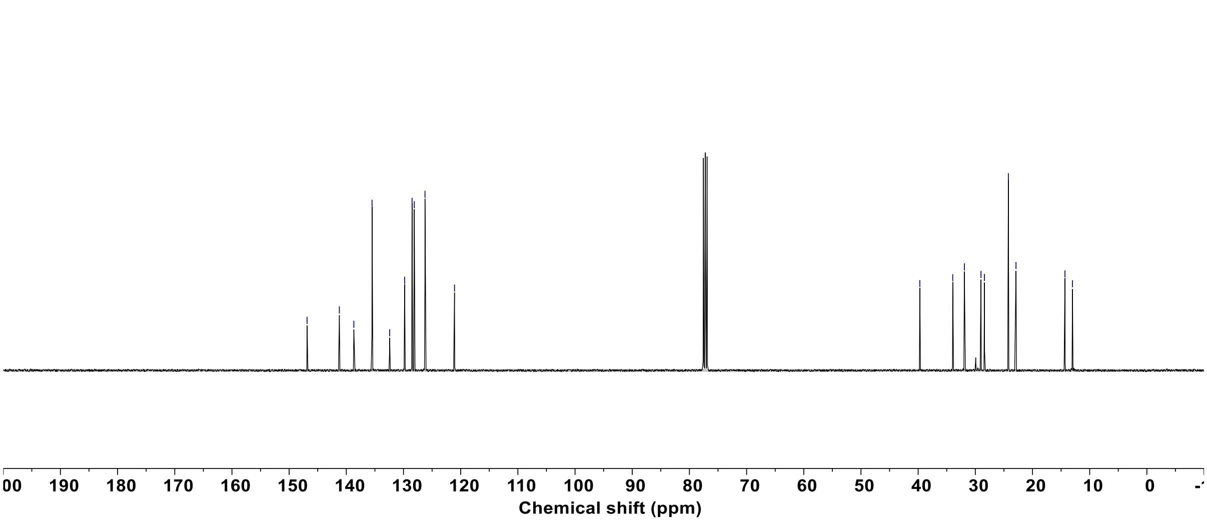

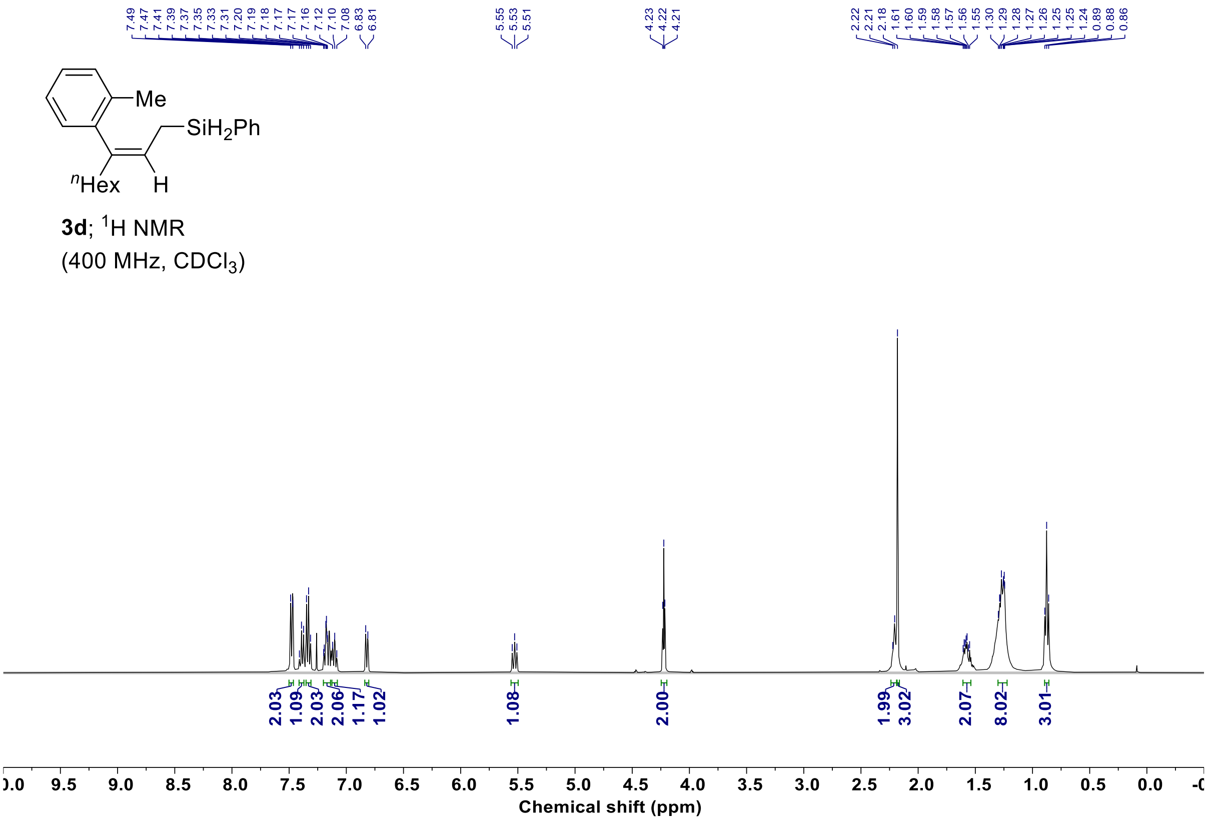

7.49 7.47 7.41 7.39 7.37 7.35 7.33 7.31 7.20 7.19 7.18 7.17 7.17 7.16 7.12 7.10 7.08 6.83 6.81
5.55 5.53 5.51
4.23 4.22 4.21
2.22 2.21 2.18 1.61 1.60 1.59 1.58 1.57 1.56 1.55 1.30 1.29 1.28 1.27 1.26 1.25 1.25 1.24 0.89 0.88 0.86
Me
SiH2Ph
nHex
H
3d; 1H NMR
(400 MHz, CDCl3)
2.03 1.09 2.03 2.06 1.17 1.02
1.08
2.00
1.99 3.02
2.07 8.02 3.01
Chemical shift (ppm)

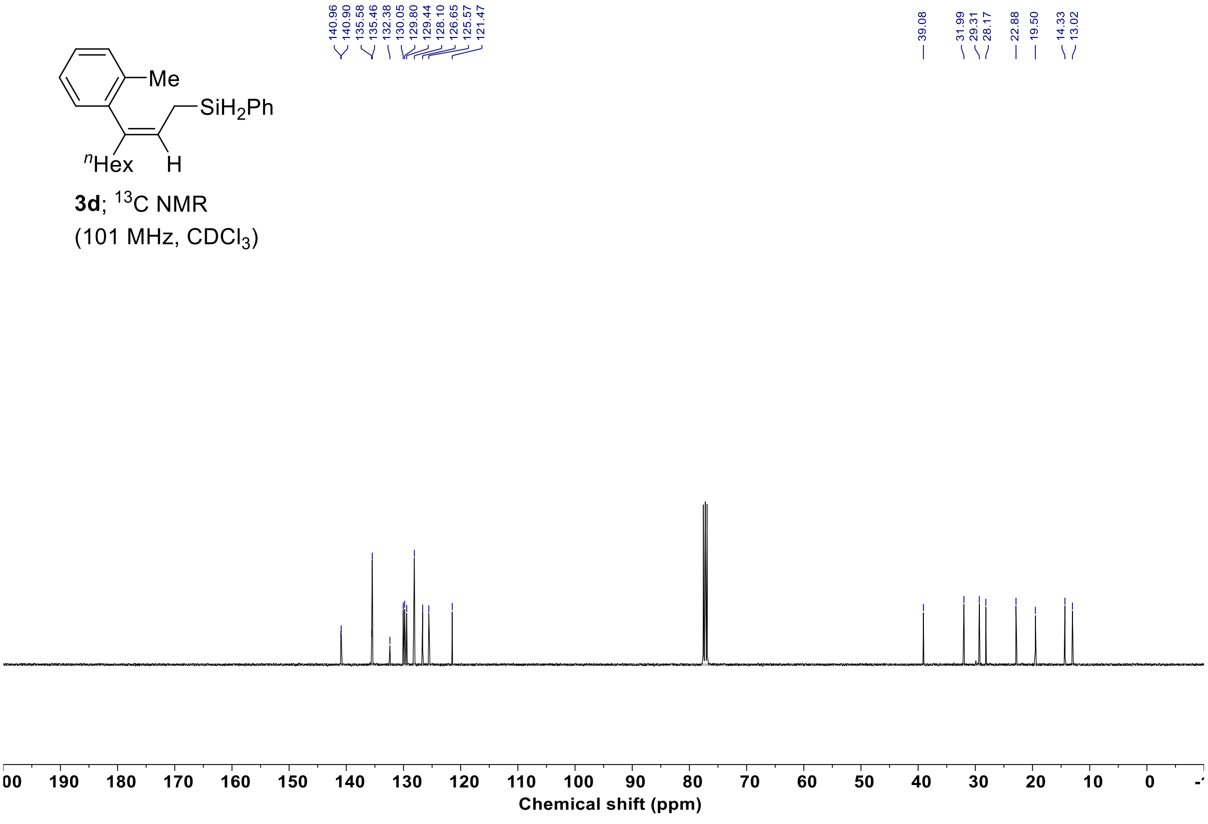

140.96 140.90 135.58 135.46 132.38 130.05 129.80 129.44 128.10 126.65 125.57 121.47
39.08 31.99 29.31 28.17 22.88 19.50 14.33 13.02
Me
SiH2Ph
nHex
H
3d; 13C NMR
(101 MHz, CDCl3)
Chemical shift (ppm)

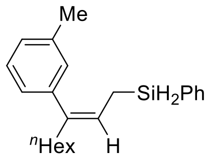

3e; ^{1}H NMR
(400 MHz, $CDCl_3$)

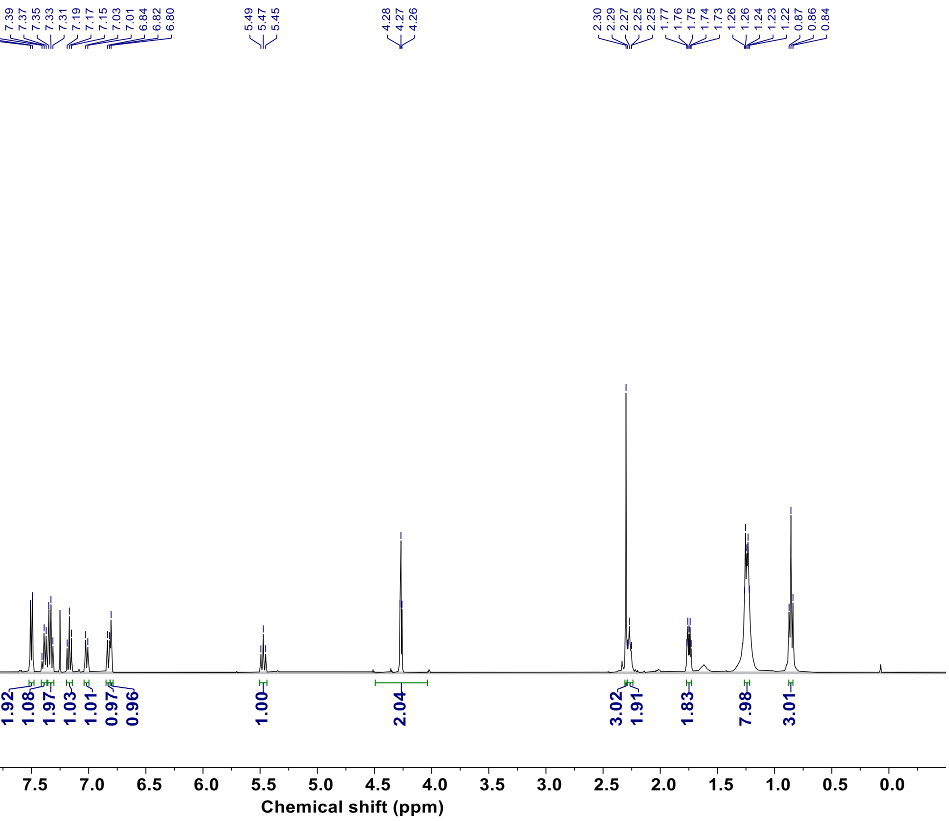

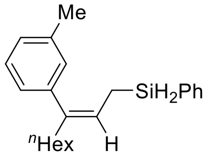

3e; ^{13}C NMR
(101 MHz, $CDCl_3$)

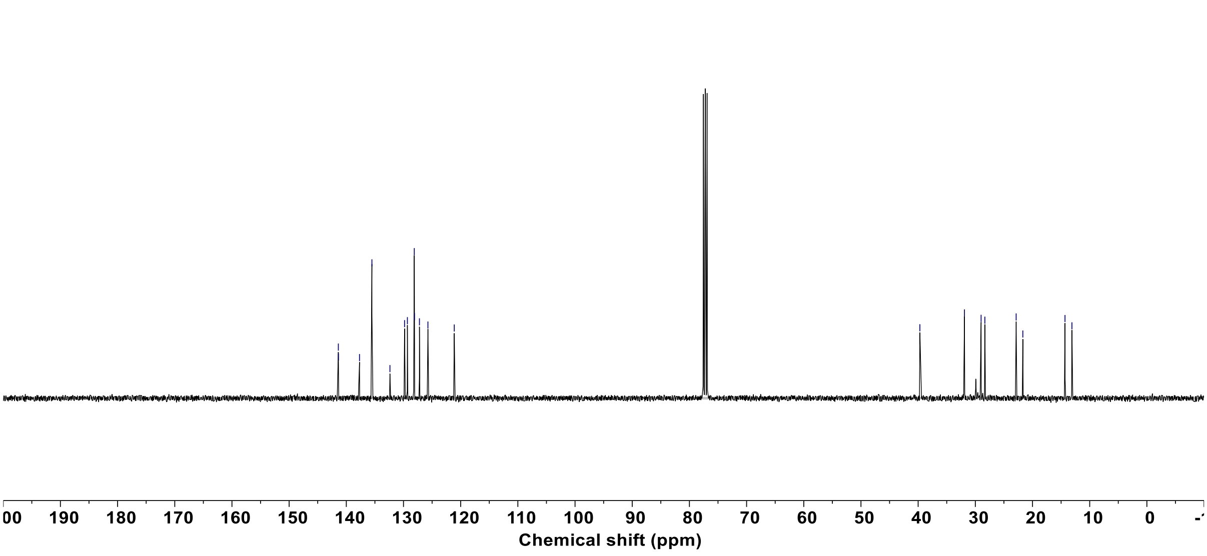

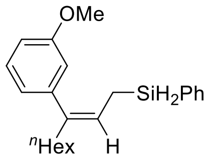

3f; ¹H NMR
(400 MHz, $CDCl_3$)

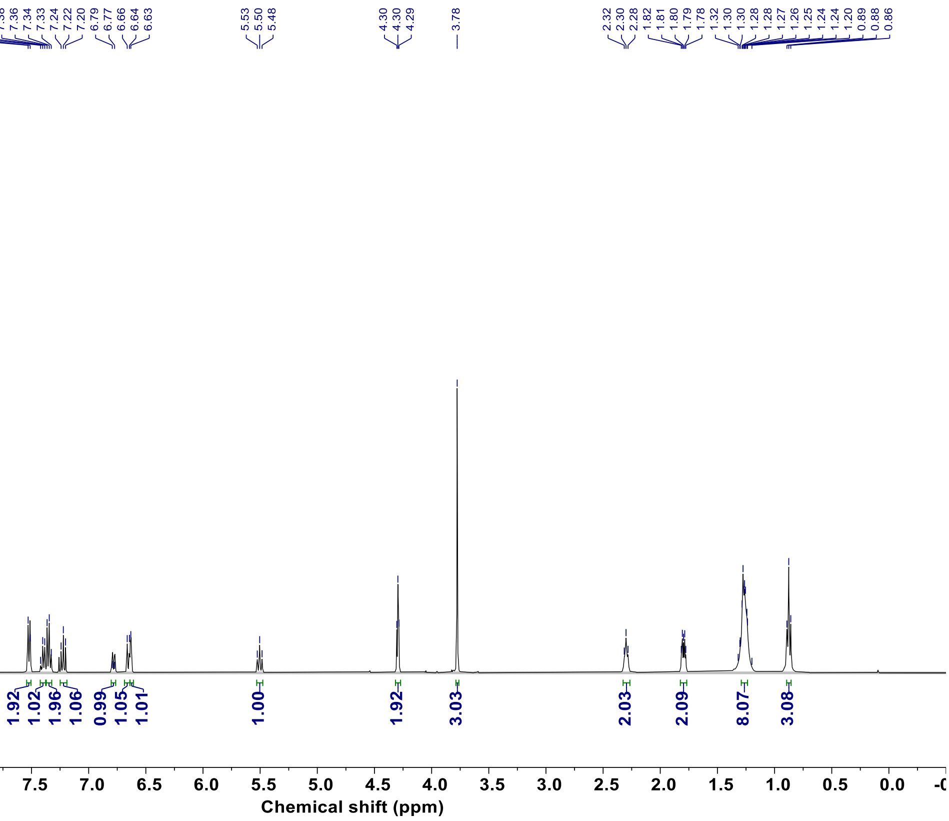

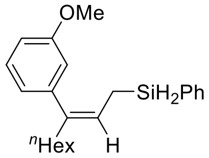

3f; ^{13}C NMR
(101 MHz, $CDCl_3$)

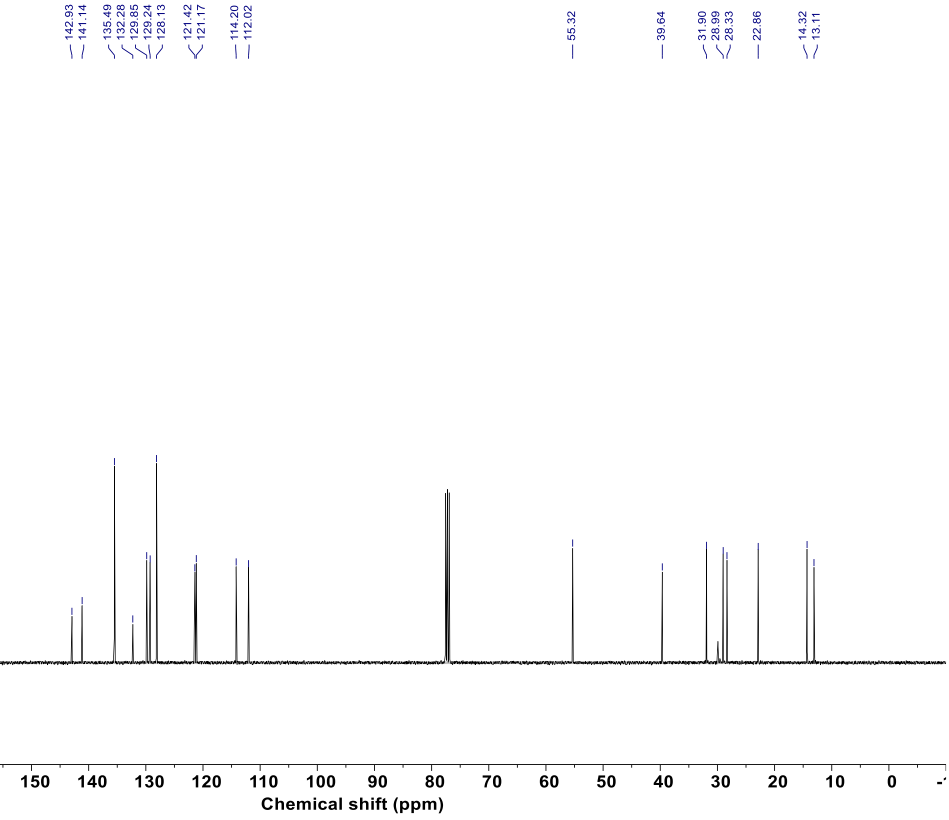

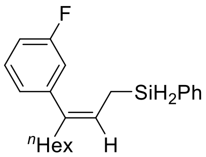

3g; ^{1}H NMR
(400 MHz, $CDCl_3$)

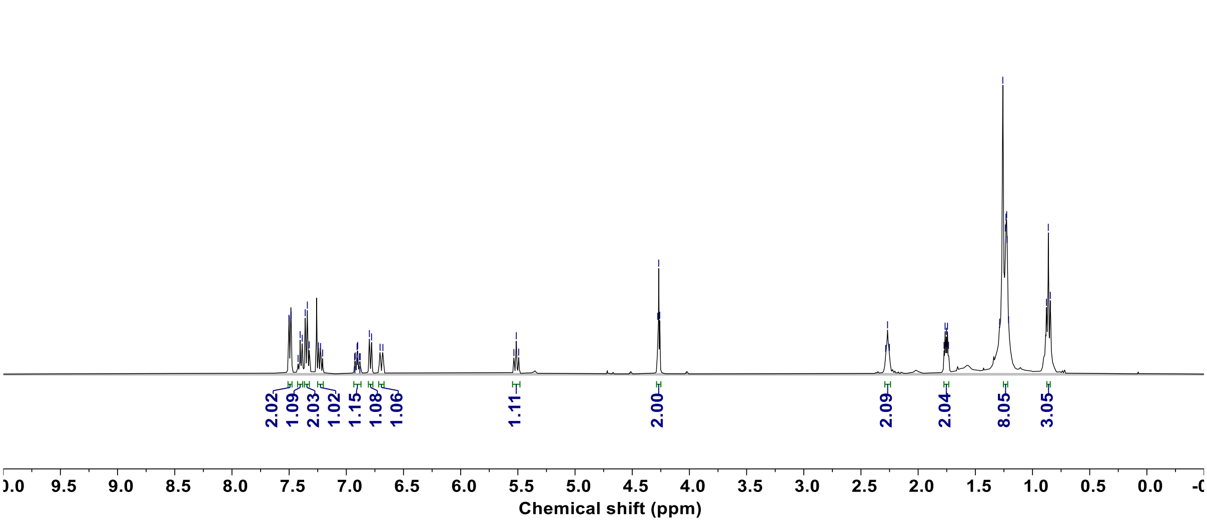

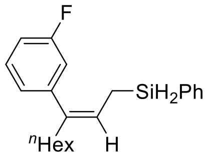

3g; ^{13}C NMR
(101 MHz, $CDCl_3$)

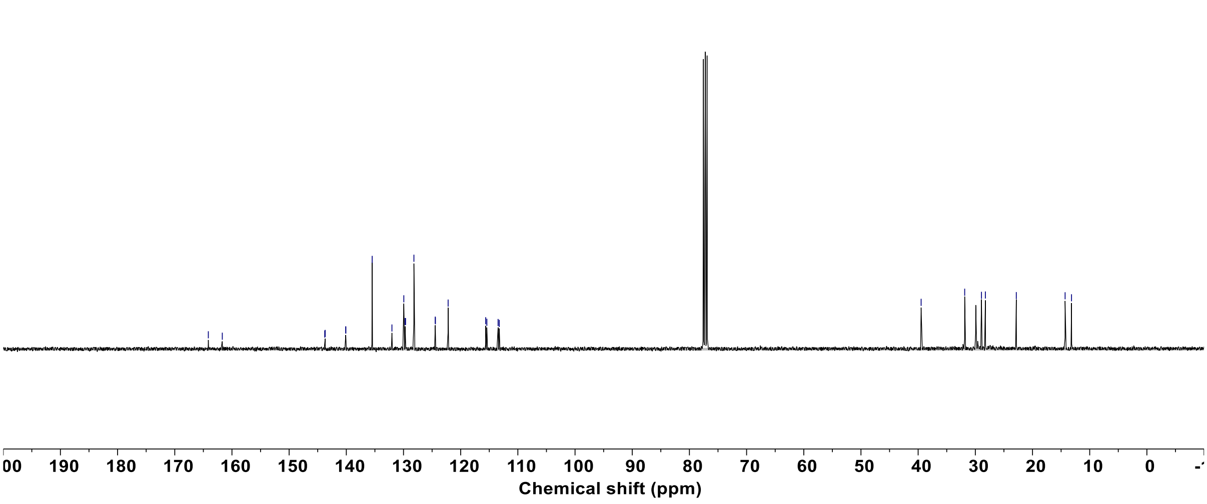

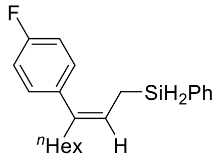

(*Z*)-**3h**; ^{1}H NMR (600 MHz, $CDCl_3$)

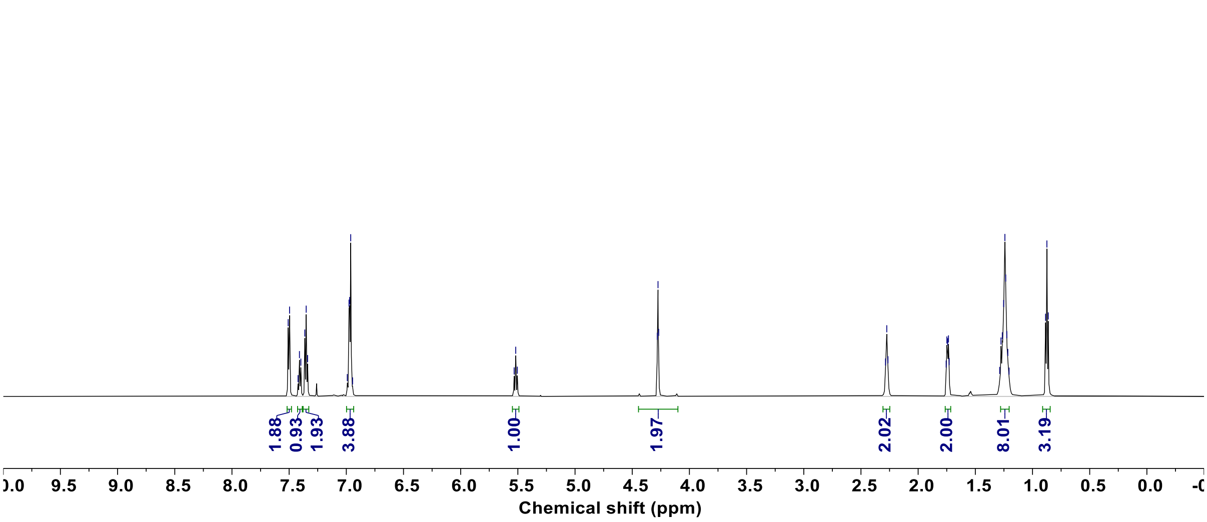

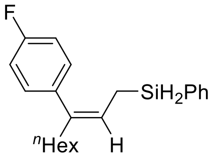

(*Z*)-**3h**; ^{13}C NMR (151 MHz, $CDCl_3$)

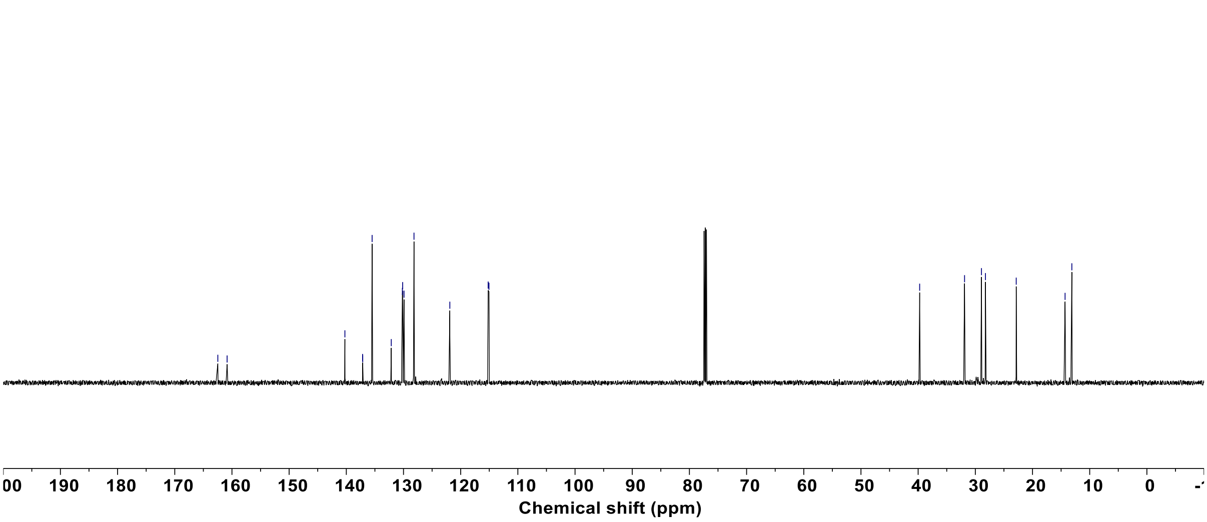

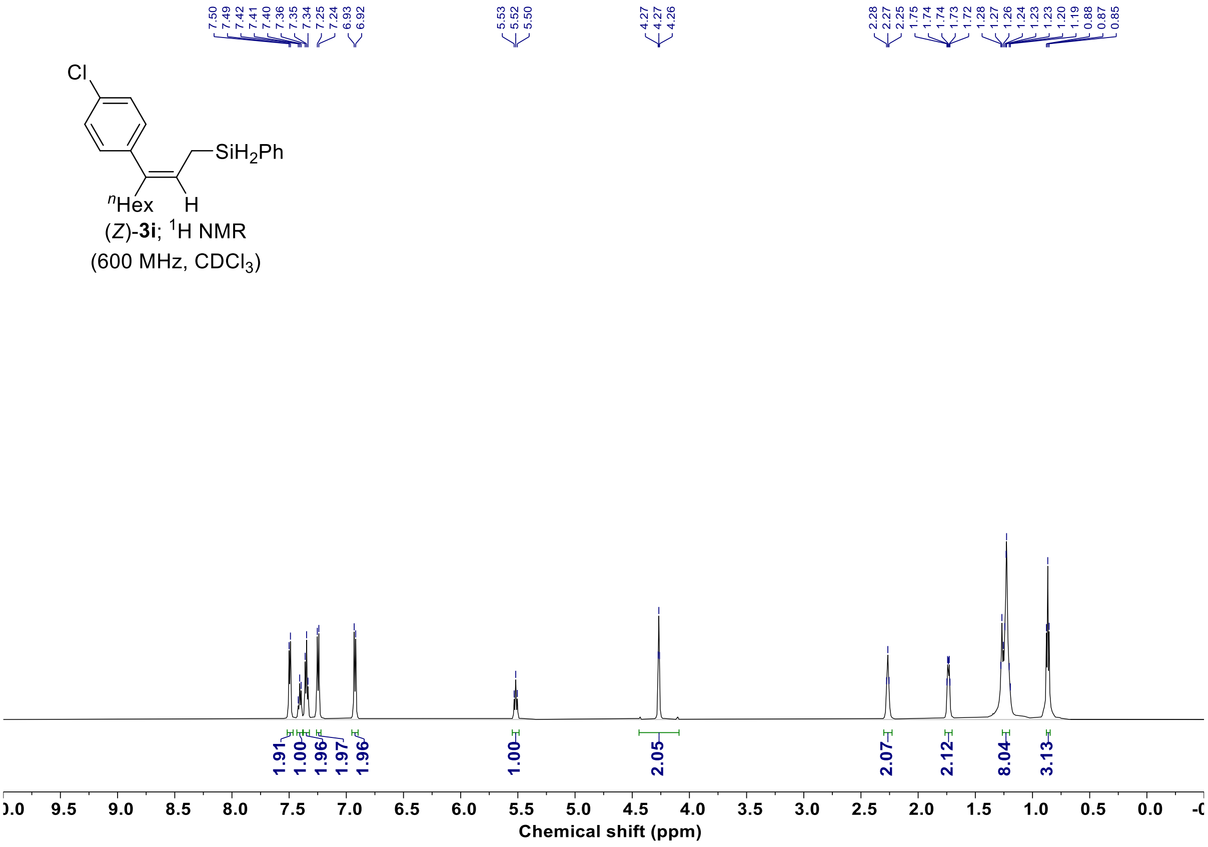

(Z)-**3i**; ^{1}H NMR (600 MHz, $CDCl_3$)

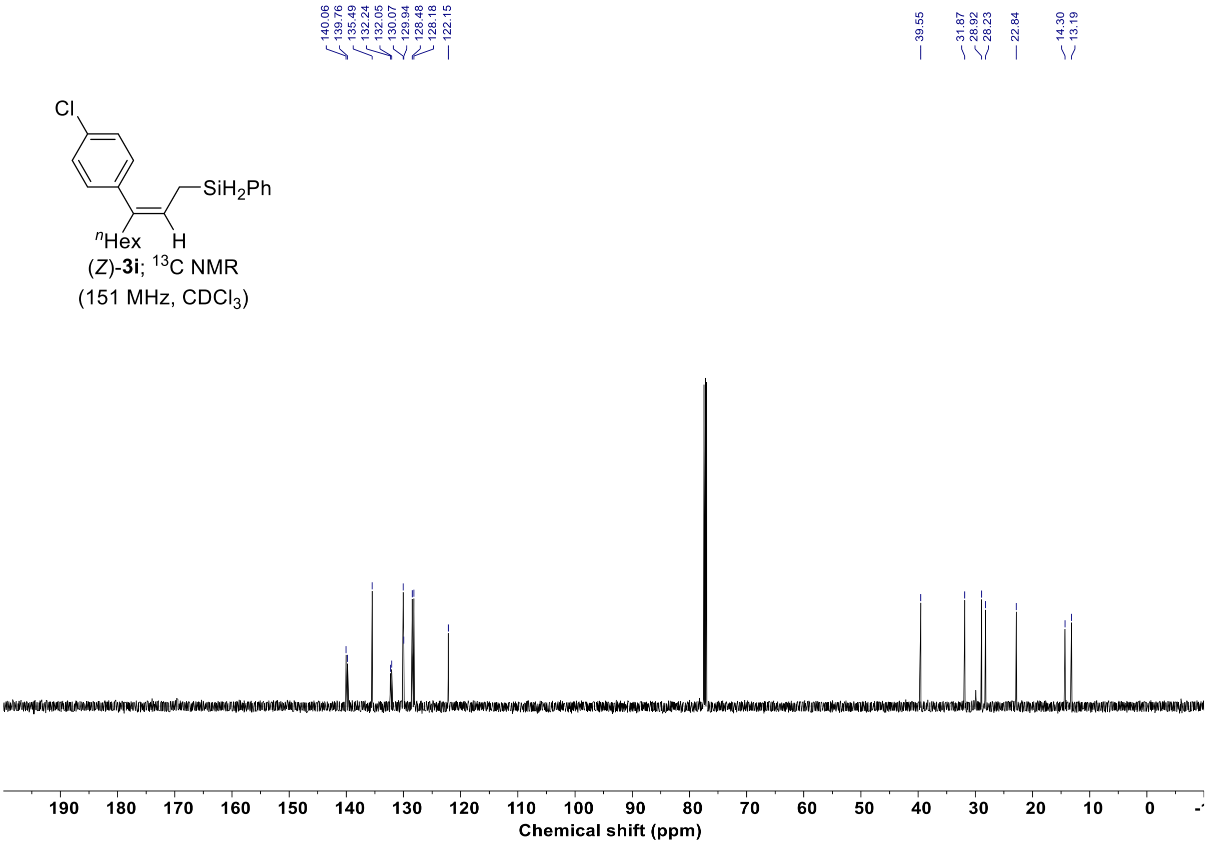

(Z)-**3i**; ^{13}C NMR (151 MHz, $CDCl_3$)

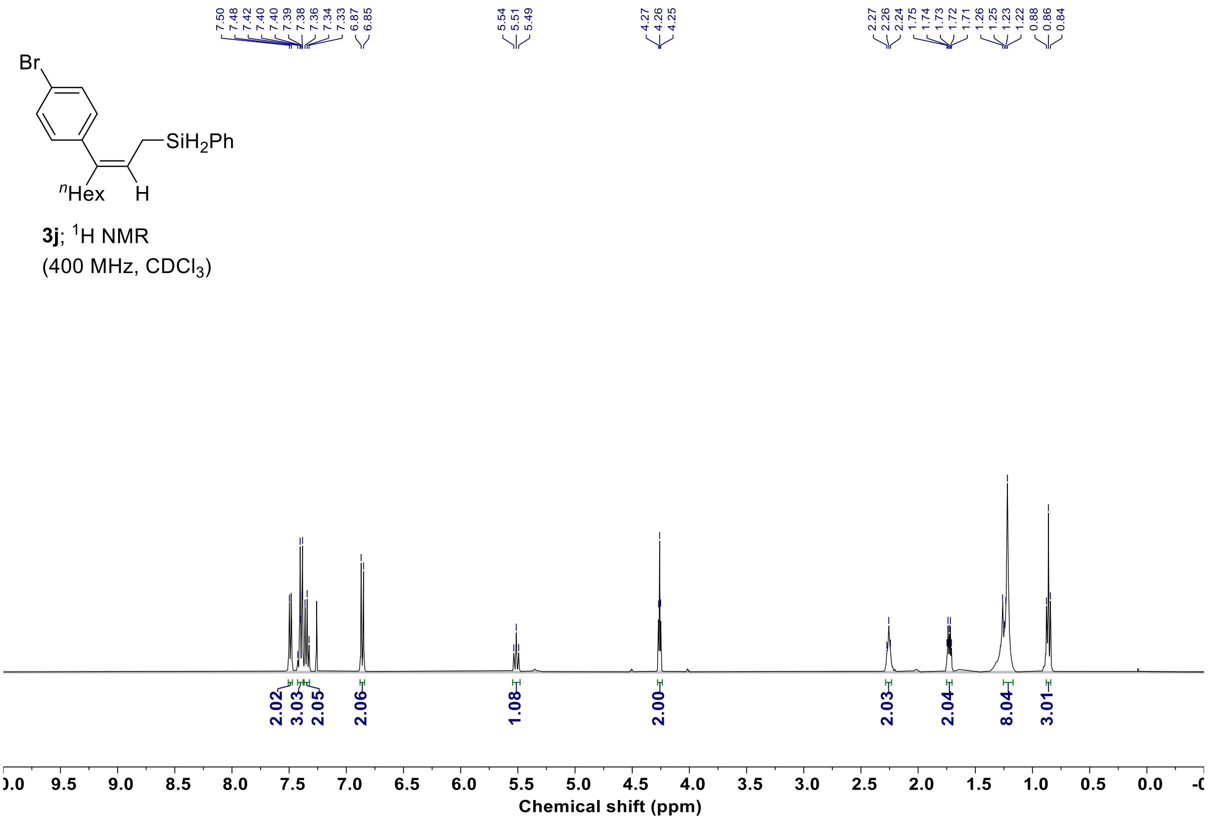

3j; ^{1}H NMR
(400 MHz, $CDCl_3$)

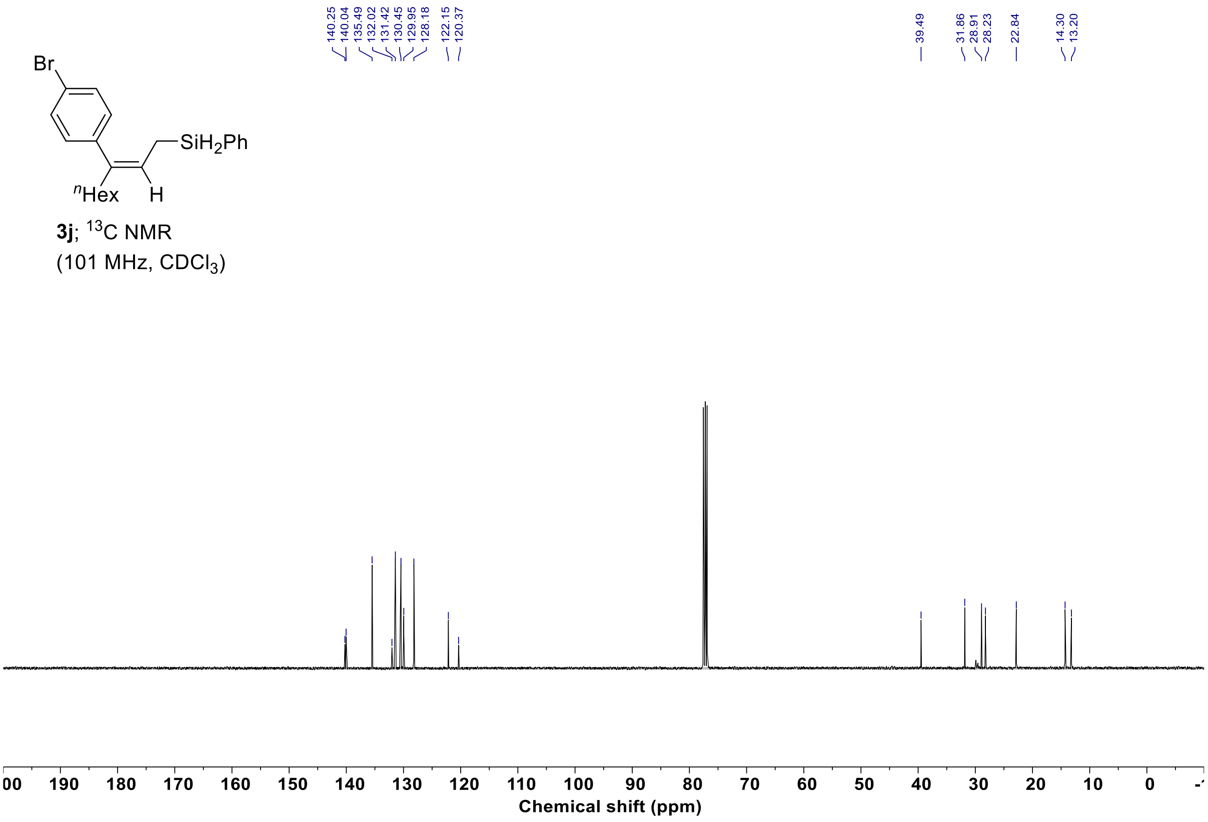

3j; ^{13}C NMR
(101 MHz, $CDCl_3$)

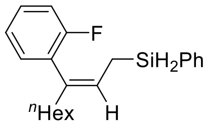

3k; ^{1}H NMR
(400 MHz, $CDCl_3$)

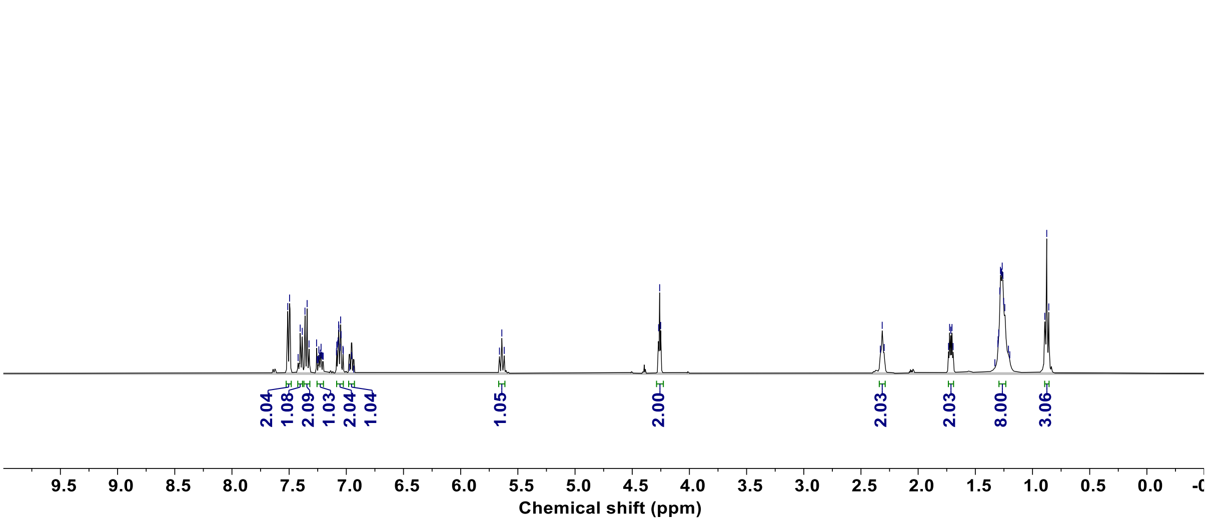

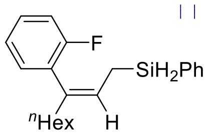

3k; ^{13}C NMR
(101 MHz, $CDCl_3$)

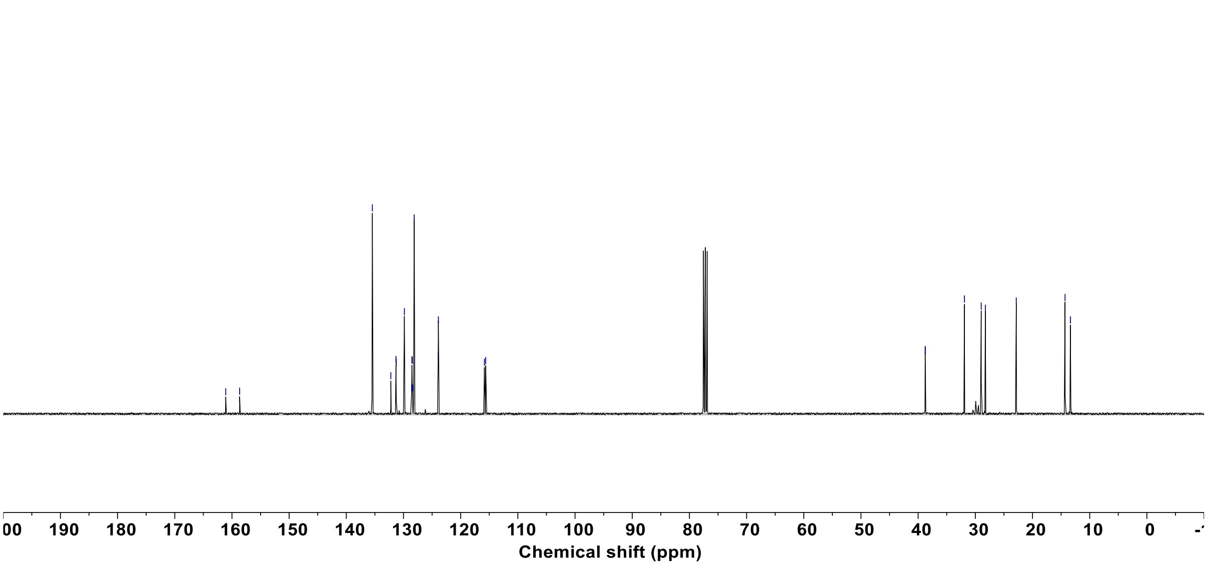

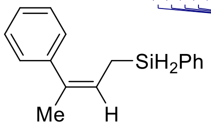

(Z)-**3l**; ^{1}H NMR
(600 MHz, $CDCl_3$)

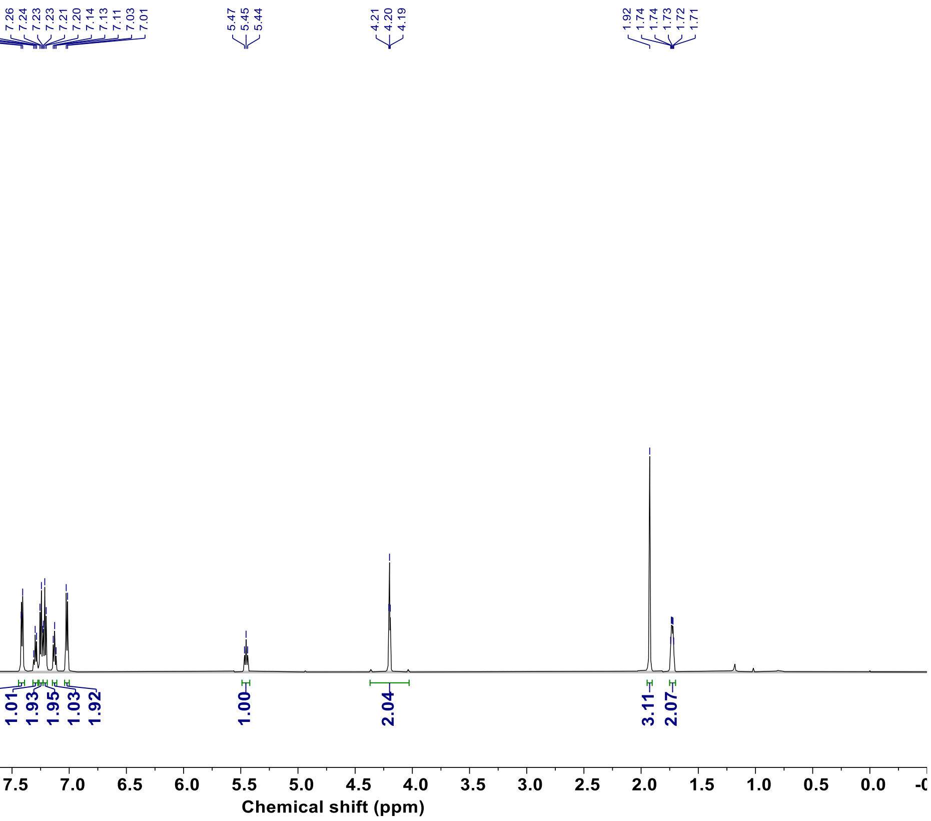

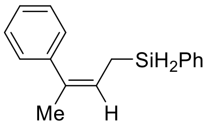

(Z)-**3l**; ^{13}C NMR
(151 MHz, $CDCl_3$)

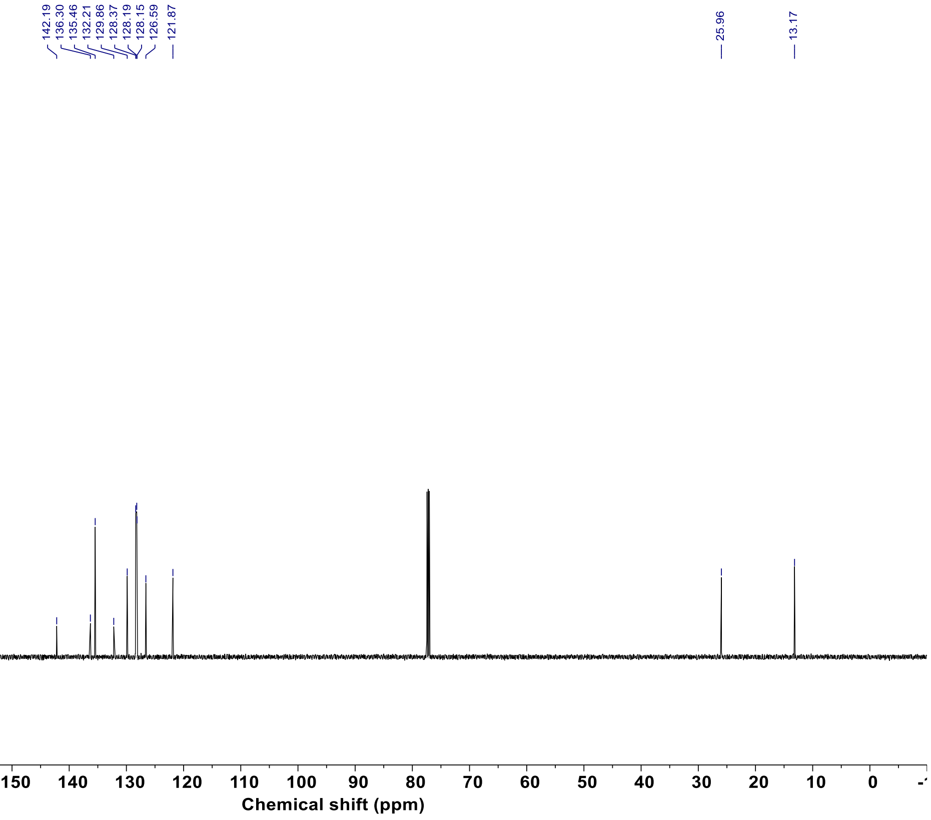

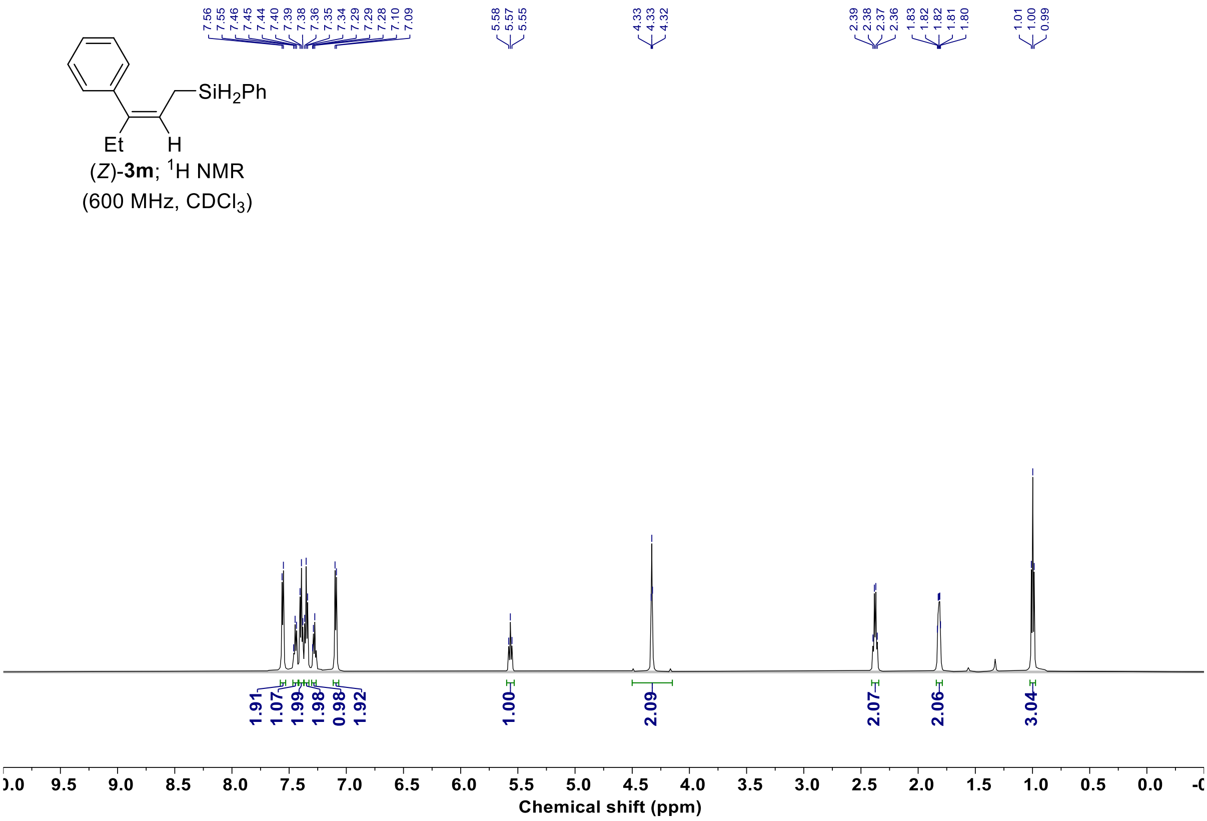

(*Z*)-**3m**; ^{1}H NMR
(600 MHz, $CDCl_3$)

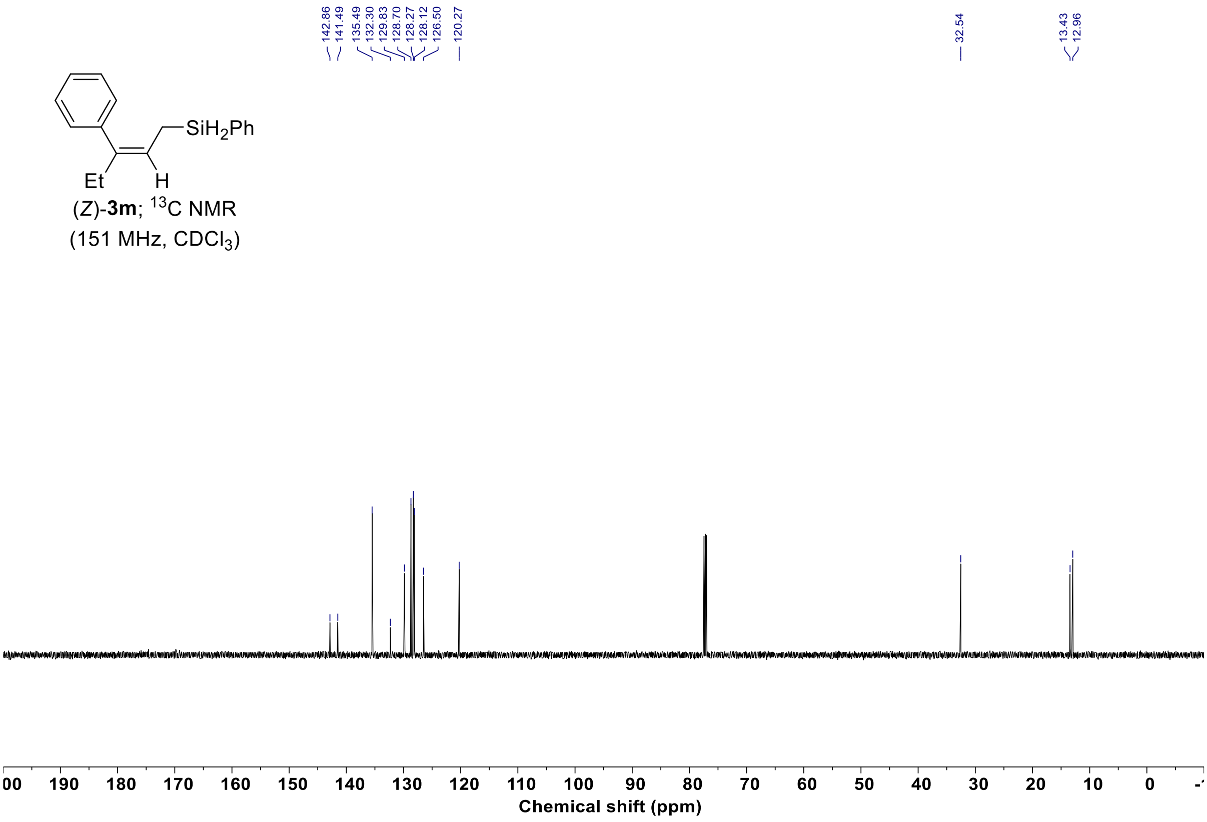

(*Z*)-**3m**; ^{13}C NMR
(151 MHz, $CDCl_3$)

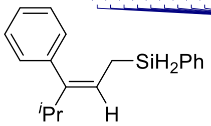

3n; ^{1}H NMR
(600 MHz, $CDCl_3$)

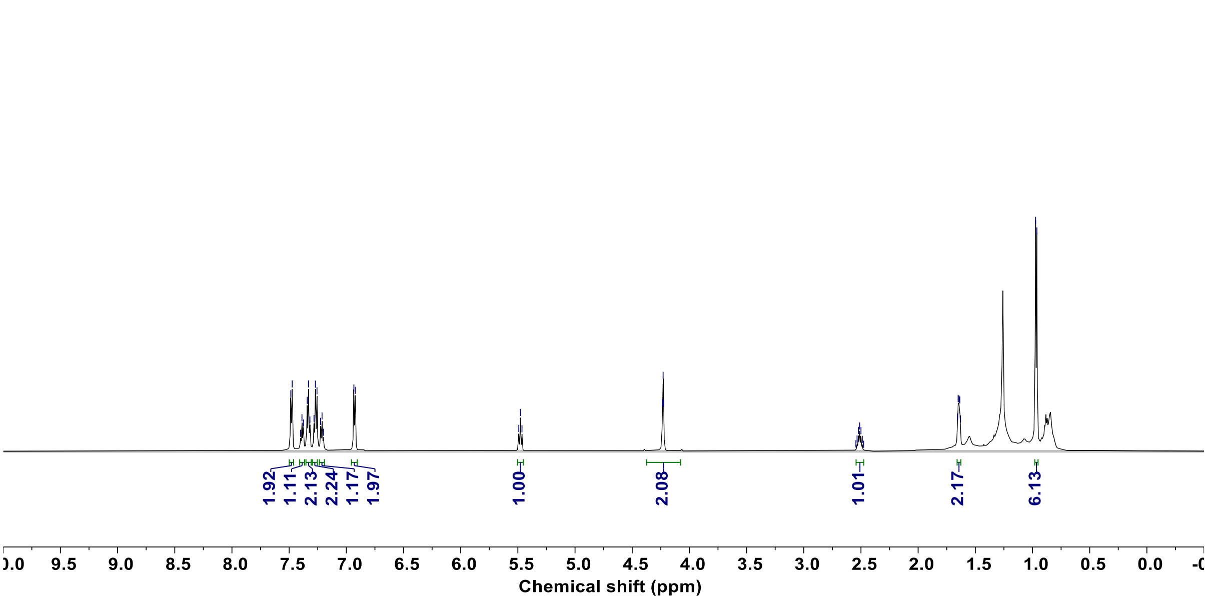

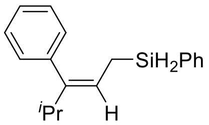

3n; ^{13}C NMR
(151 MHz, $CDCl_3$)

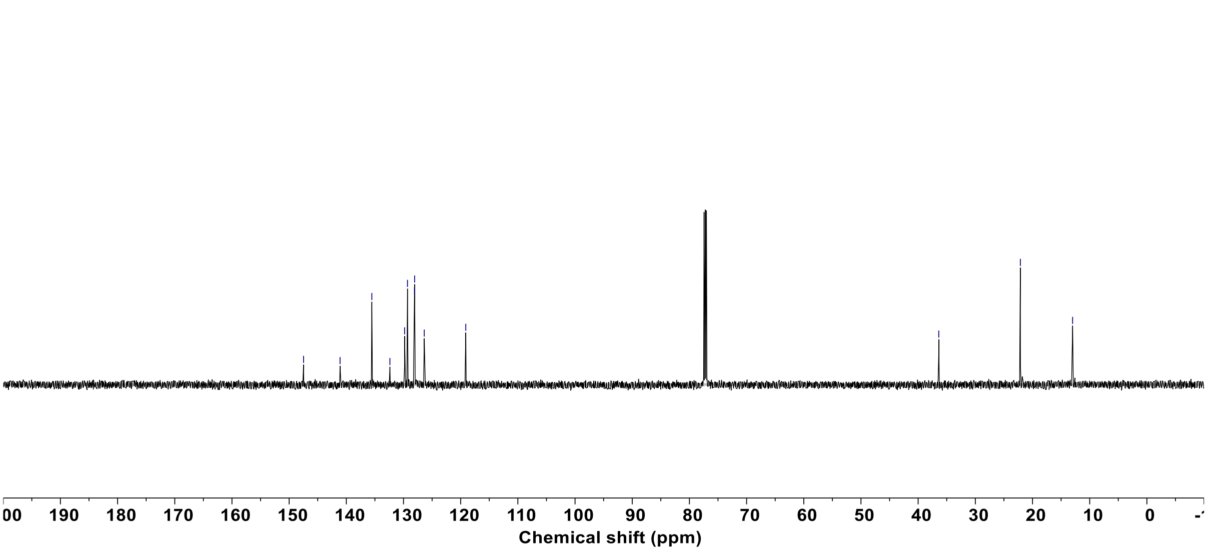

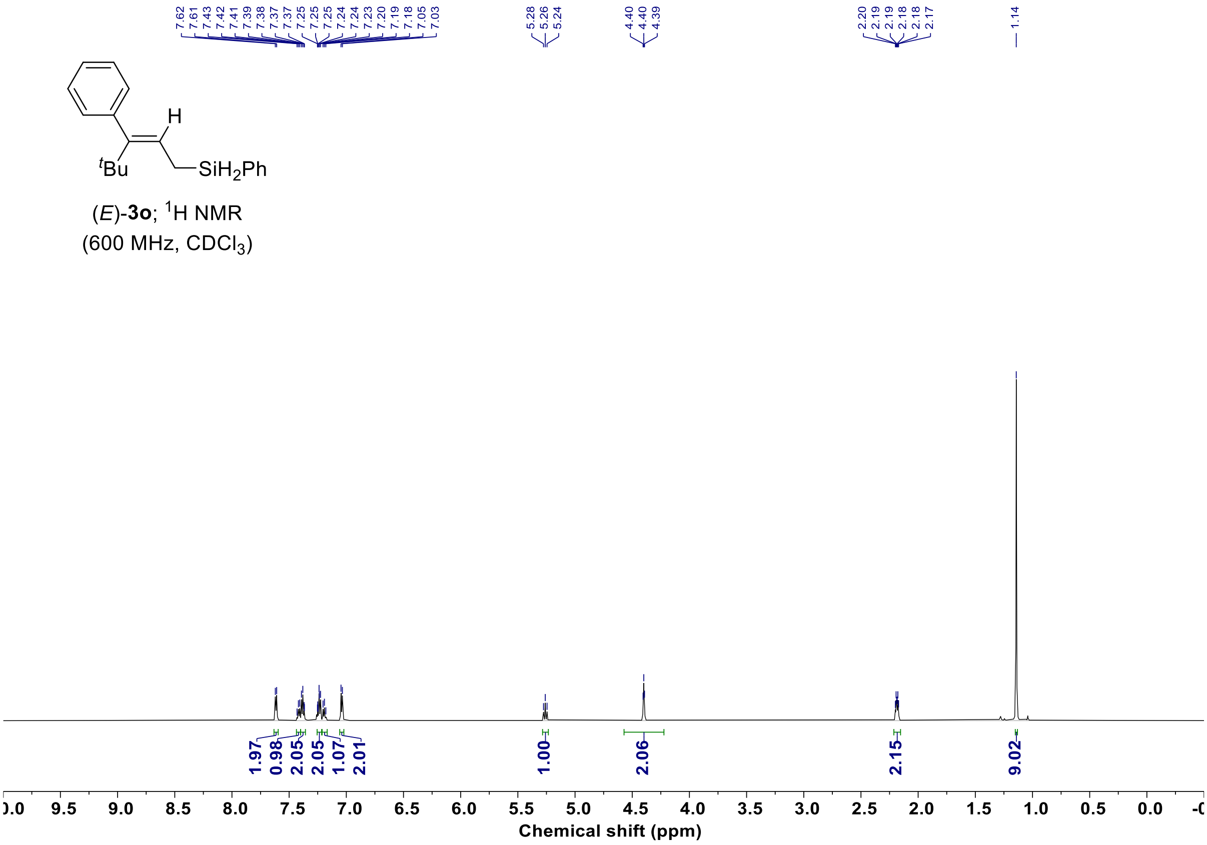
7.62 7.61 7.43 7.42 7.41 7.39 7.38 7.37 7.37 7.25 7.25 7.25 7.24 7.24 7.23 7.20 7.19 7.18 7.05 7.03
5.28 5.26 5.24
4.40 4.40 4.39
2.20 2.19 2.19 2.18 2.18 2.17
1.14
H
tBu
SiH2Ph
(E)-3o; 1H NMR
(600 MHz, CDCl3)
1.97 0.98 2.05 2.05 1.07 2.01
1.00
2.06
2.15
9.02
Chemical shift (ppm)

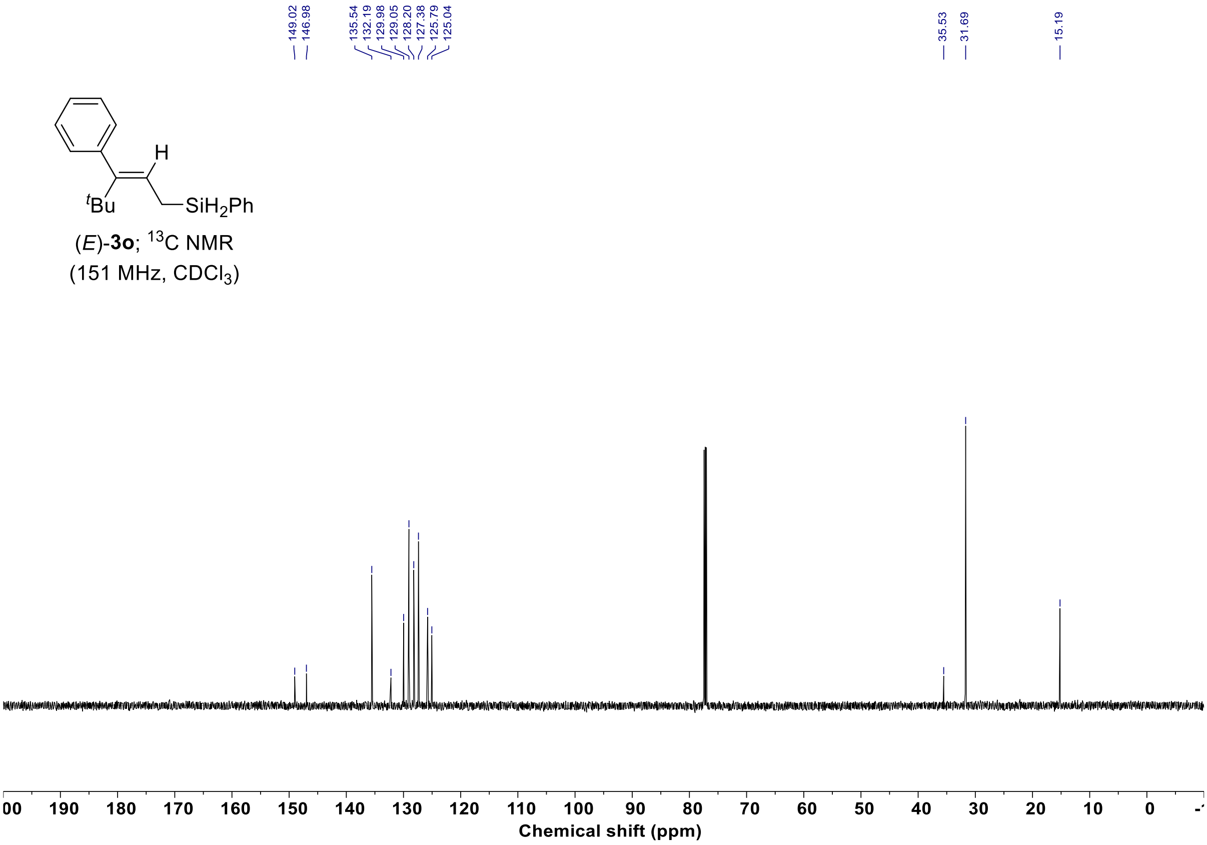
149.02 146.98
135.54 132.19 129.98 129.05 128.20 127.38 125.79 125.04
35.53 31.69
15.19
H
tBu
SiH2Ph
(E)-3o; 13C NMR
(151 MHz, CDCl3)
Chemical shift (ppm)

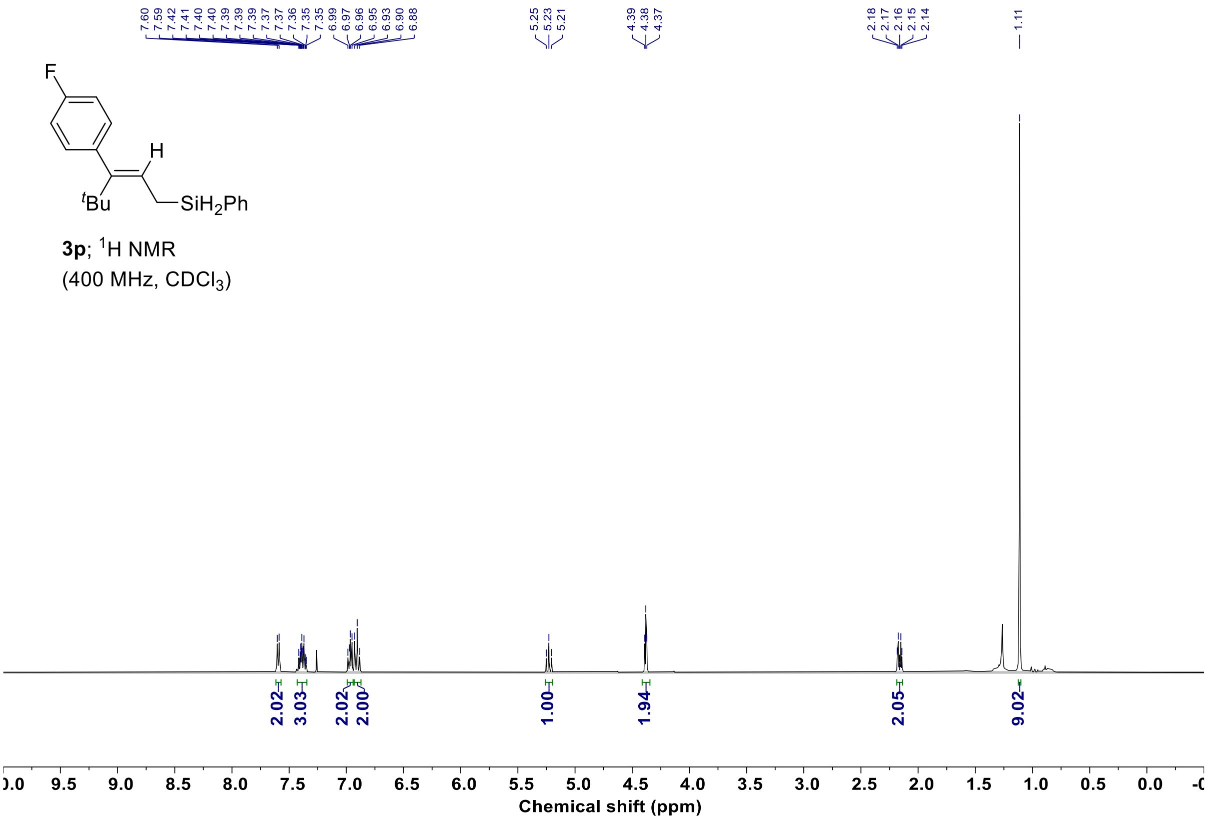
7.60 7.59 7.42 7.41 7.40 7.40 7.39 7.39 7.39 7.37 7.37 7.36 7.35 7.35 6.99 6.97 6.96 6.95 6.93 6.90 6.88
5.25 5.23 5.21
4.39 4.38 4.37
2.18 2.17 2.16 2.15 2.14
1.11
F
H
tBu
SiH2Ph
3p; 1H NMR
(400 MHz, CDCl3)
2.02 3.03 2.02 2.00 1.00 1.94 2.05 9.02
Chemical shift (ppm)

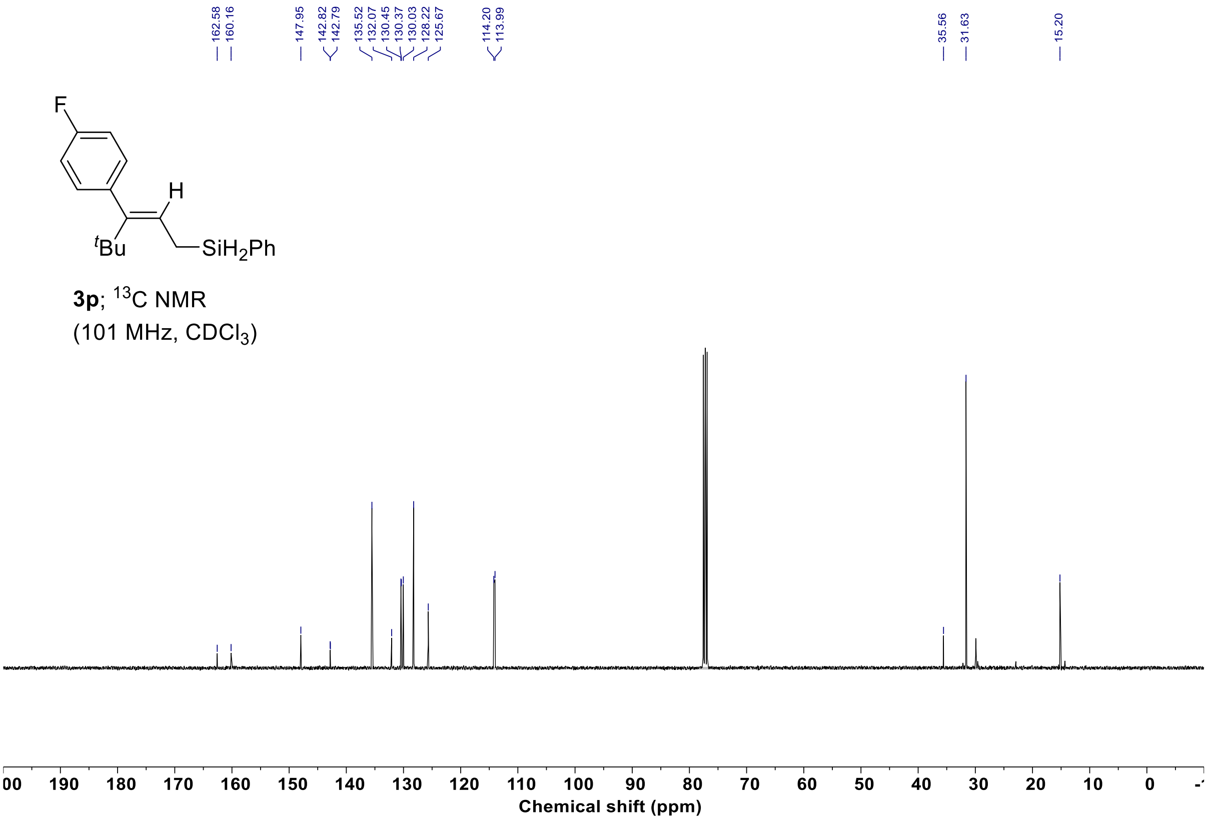
162.58 160.16 147.95 142.82 142.79 135.52 132.07 130.45 130.37 130.03 128.22 125.67 114.20 113.99
35.56 31.63 15.20
F
H
tBu
SiH2Ph
3p; 13C NMR
(101 MHz, CDCl3)
Chemical shift (ppm)

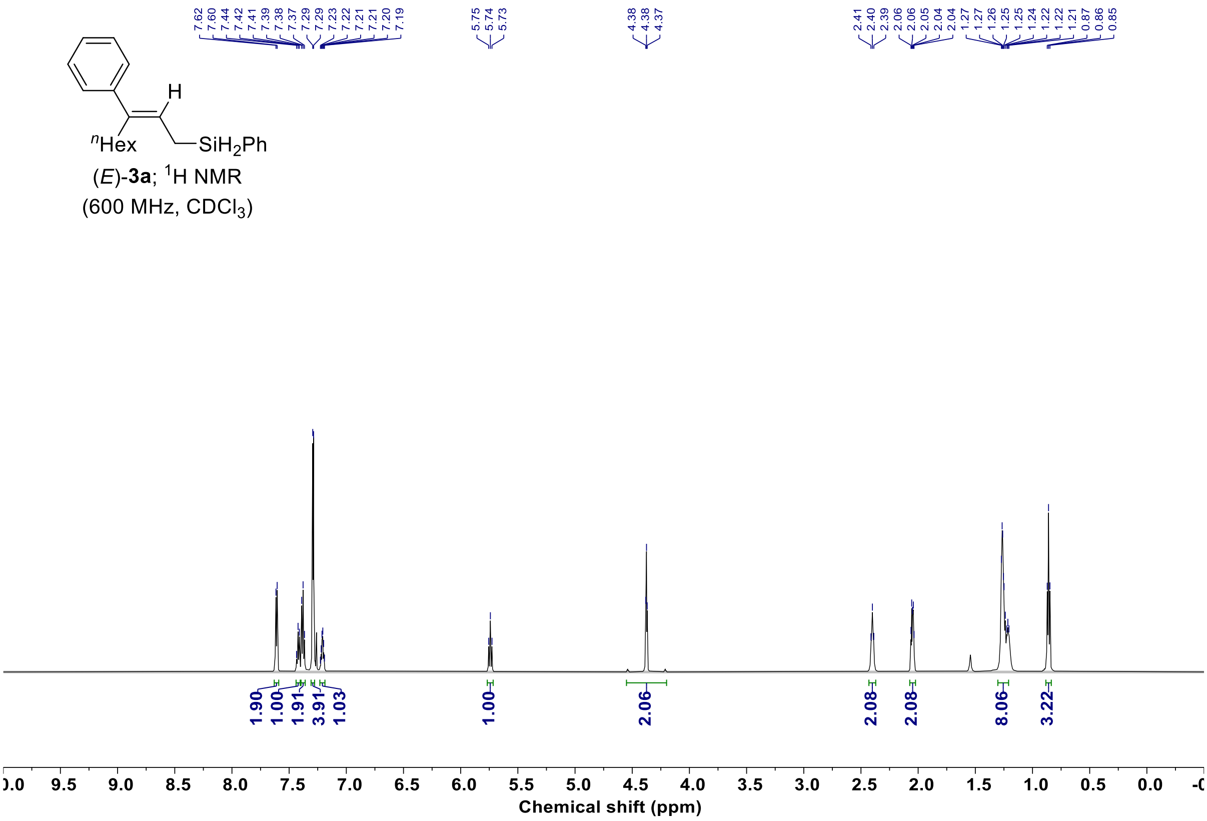

7.62 7.60 7.44 7.42 7.41 7.39 7.38 7.37 7.29 7.29 7.23 7.22 7.21 7.21 7.20 7.19
5.75 5.74 5.73
4.38 4.38 4.37
2.41 2.40 2.39 2.06 2.05 2.04 2.04 1.27 1.27 1.26 1.25 1.25 1.24 1.22 1.22 1.21 0.87 0.86 0.85
H
^{n}Hex
SiH$_2$Ph
(*E*)-**3a**; ^{1}H NMR
(600 MHz, CDCl$_3$)
1.90 1.00 1.91 3.91 1.03 1.00 2.06 2.08 2.08 8.06 3.22
Chemical shift (ppm)

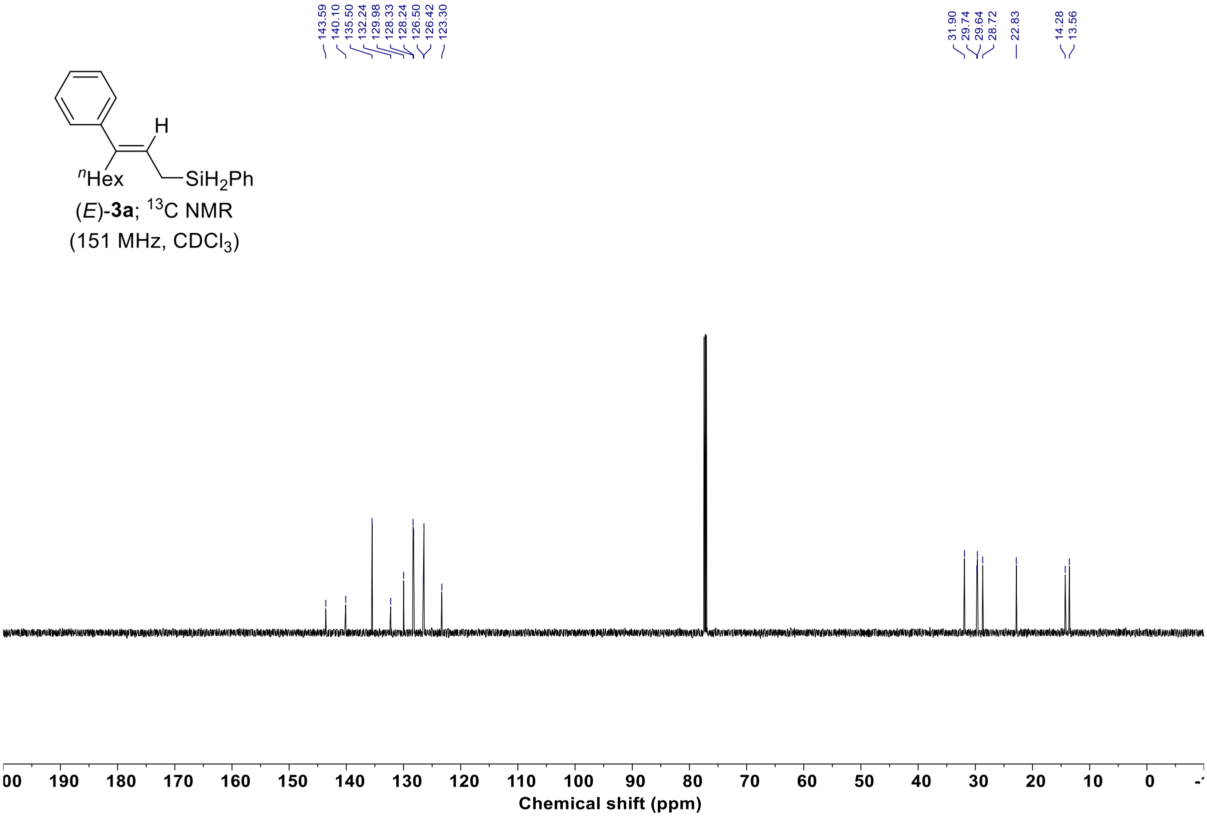

143.59 140.10 135.50 132.24 129.98 128.33 128.24 126.50 126.42 123.30
31.90 29.74 29.64 28.72 22.83 14.28 13.56
H
^{n}Hex
SiH$_2$Ph
(*E*)-**3a**; ^{13}C NMR
(151 MHz, CDCl$_3$)
Chemical shift (ppm)

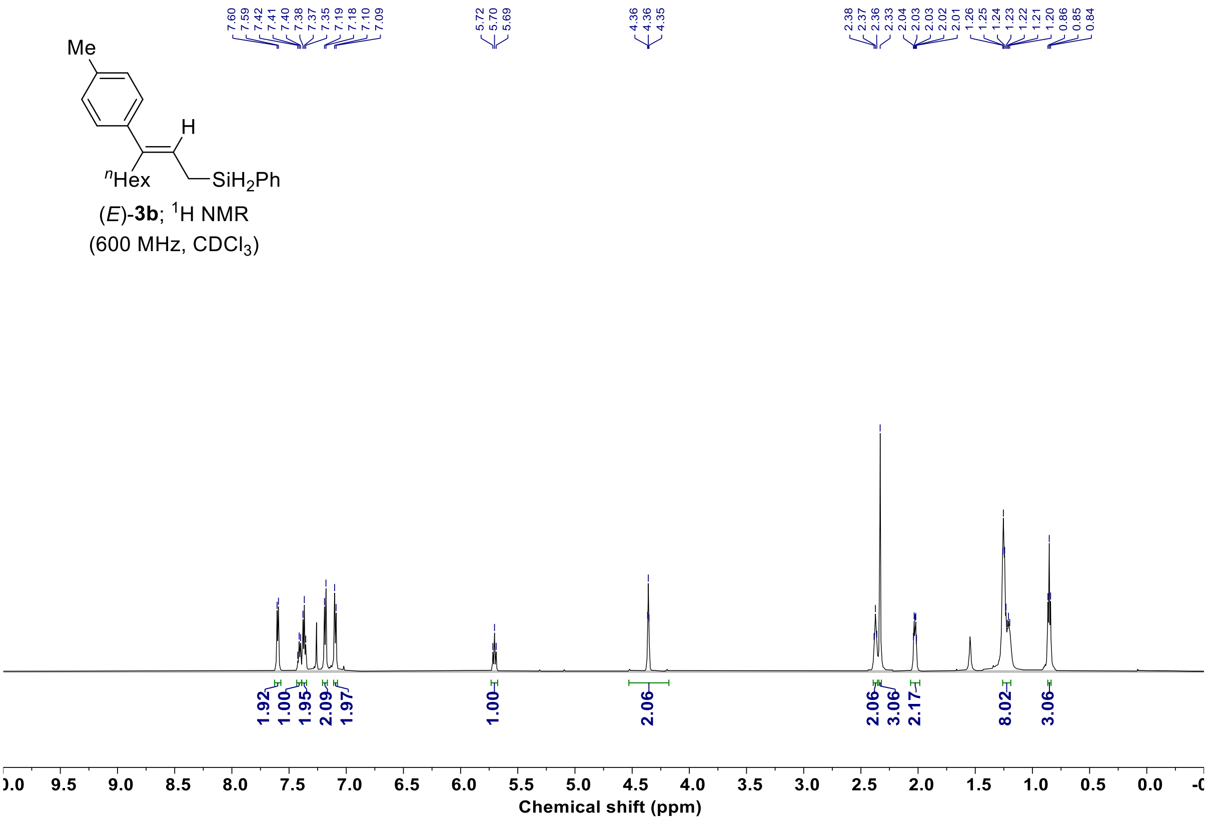
Me
H
nHex
SiH2Ph
(E)-3b; 1H NMR
(600 MHz, CDCl3)
Chemical shift (ppm)

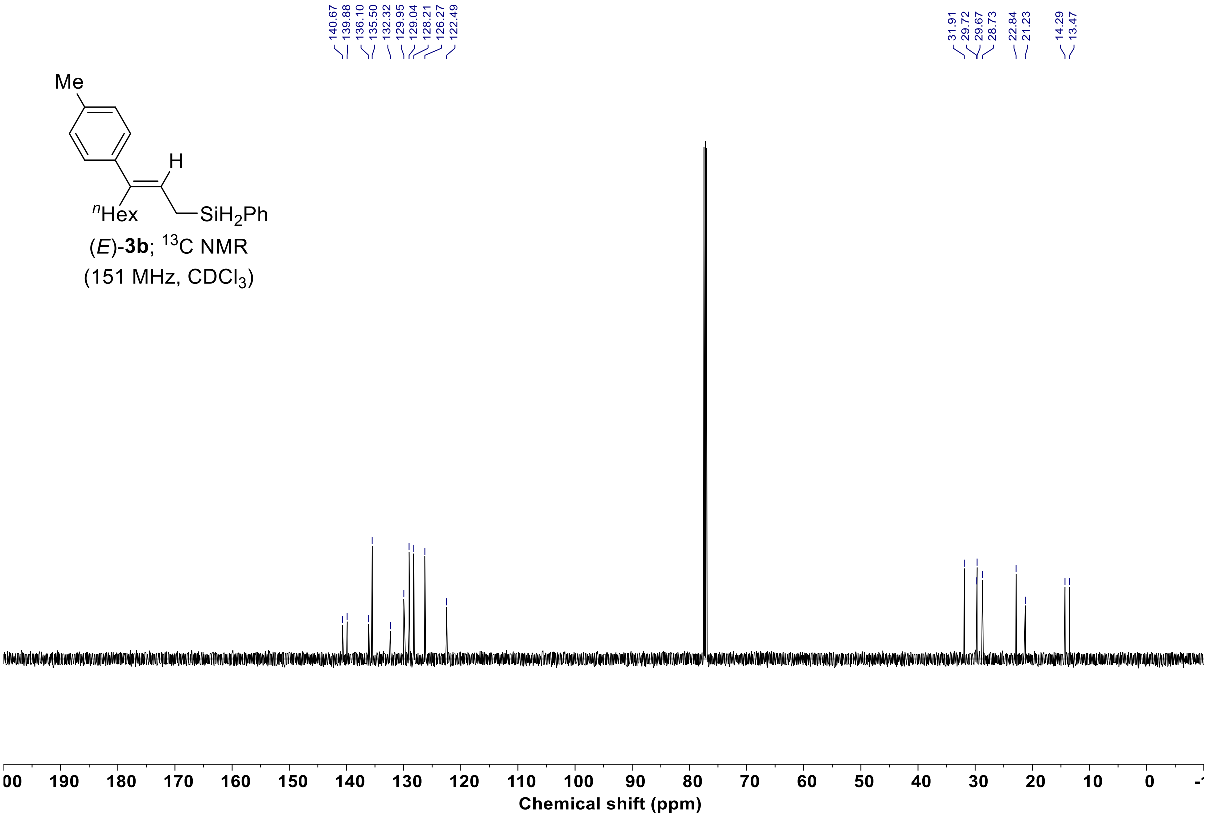
Me
H
nHex
SiH2Ph
(E)-3b; 13C NMR
(151 MHz, CDCl3)
Chemical shift (ppm)

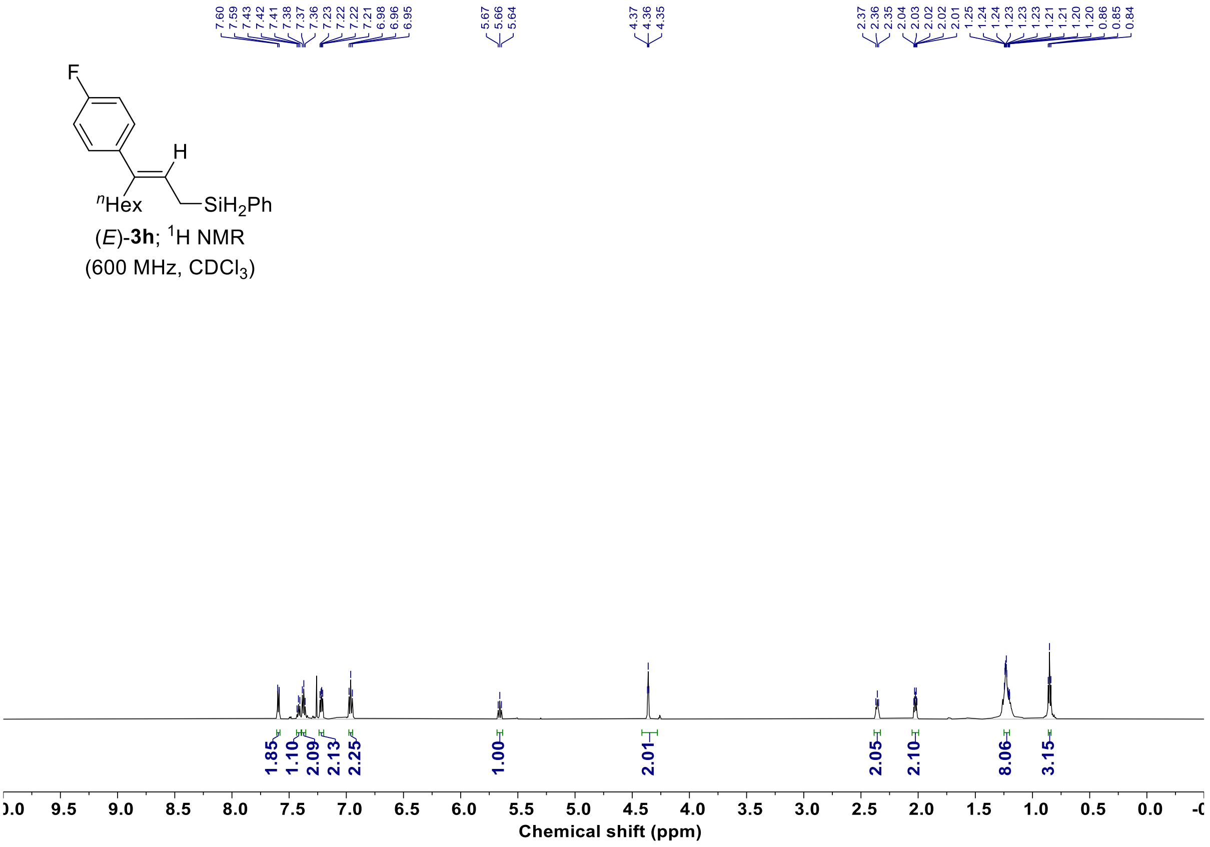

(*E*)-**3h**; ^{1}H NMR
(600 MHz, $CDCl_3$)

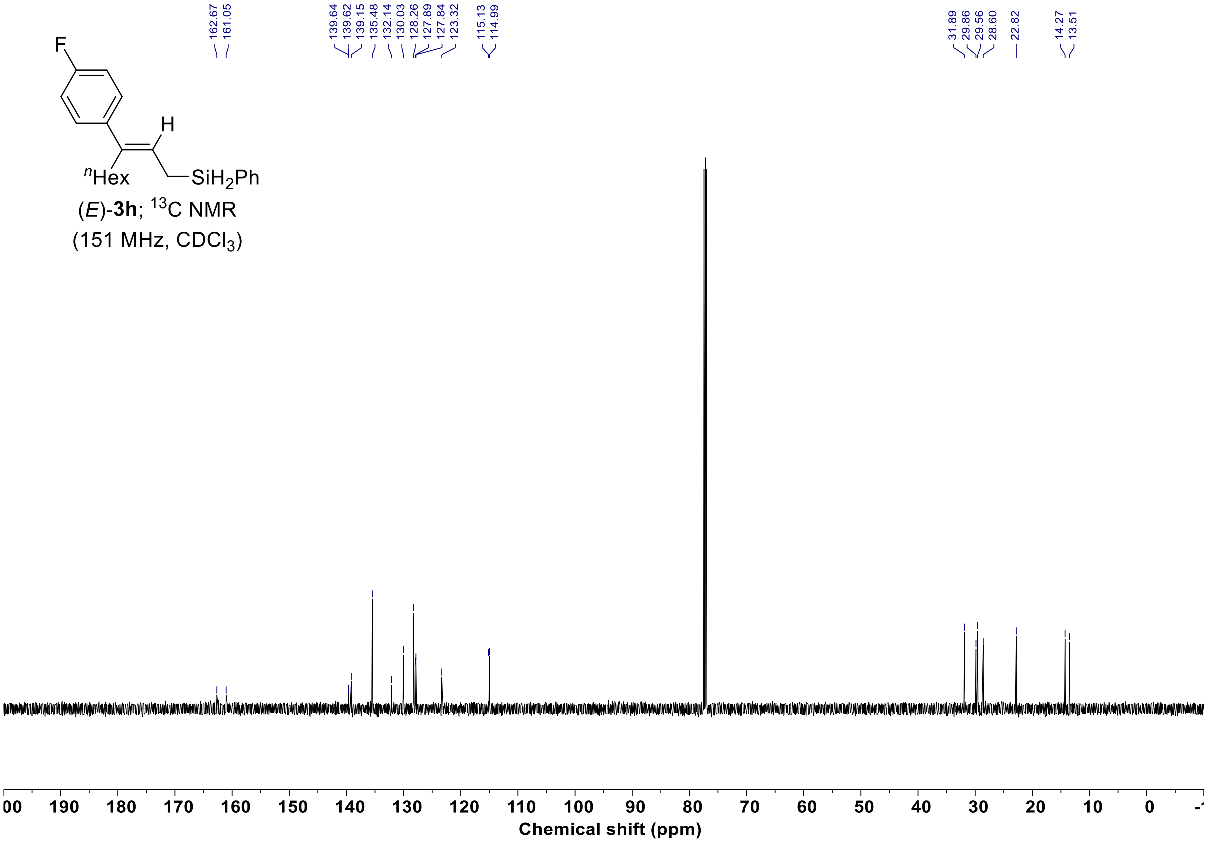

(*E*)-**3h**; ^{13}C NMR
(151 MHz, $CDCl_3$)

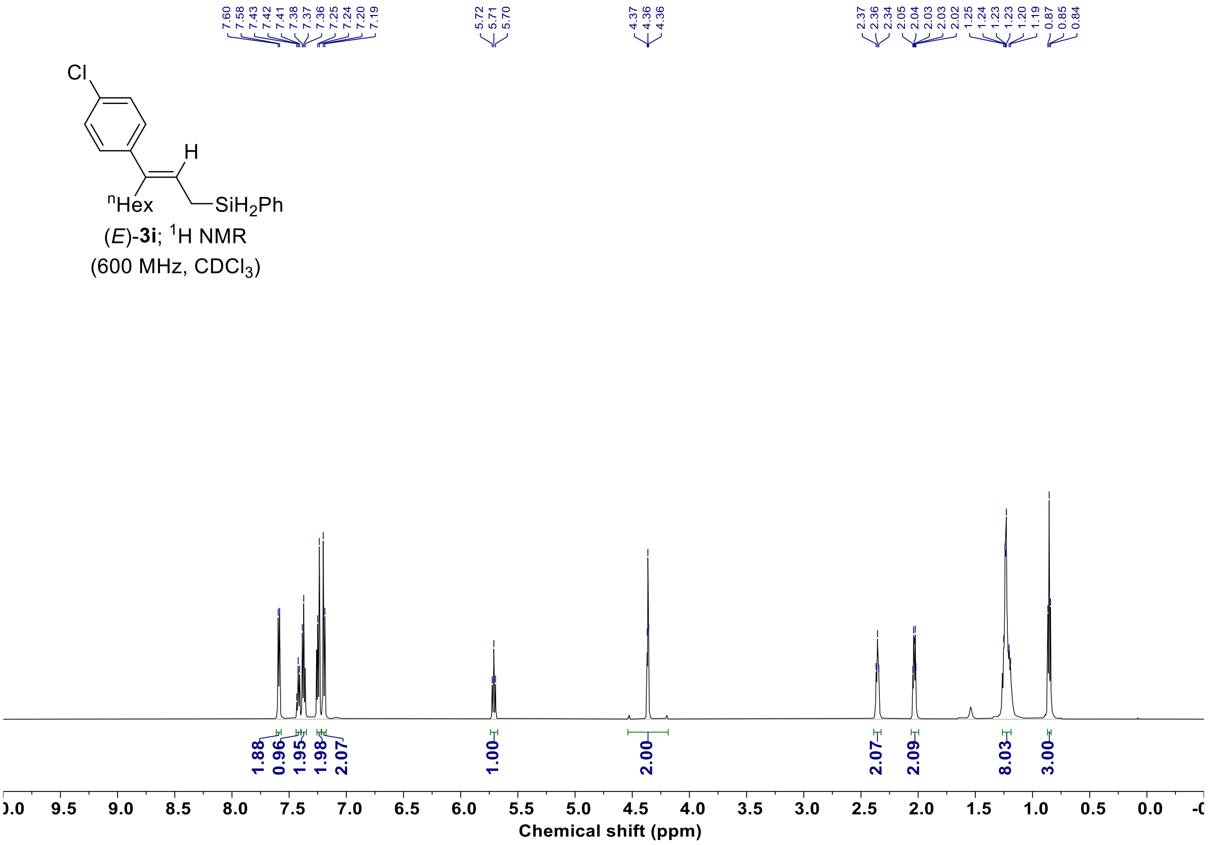

(E)-**3i**; ^{1}H NMR (600 MHz, $CDCl_3$)

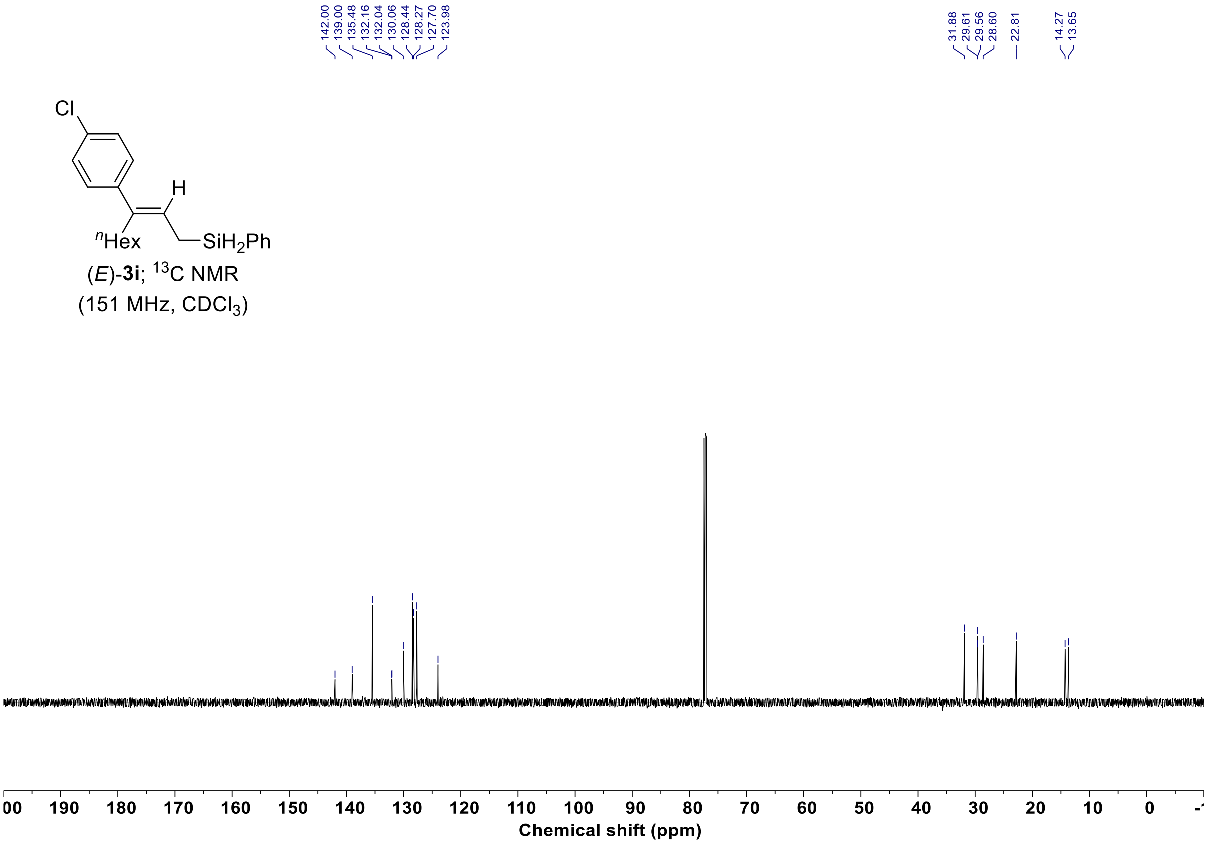

(E)-**3i**; ^{13}C NMR (151 MHz, $CDCl_3$)

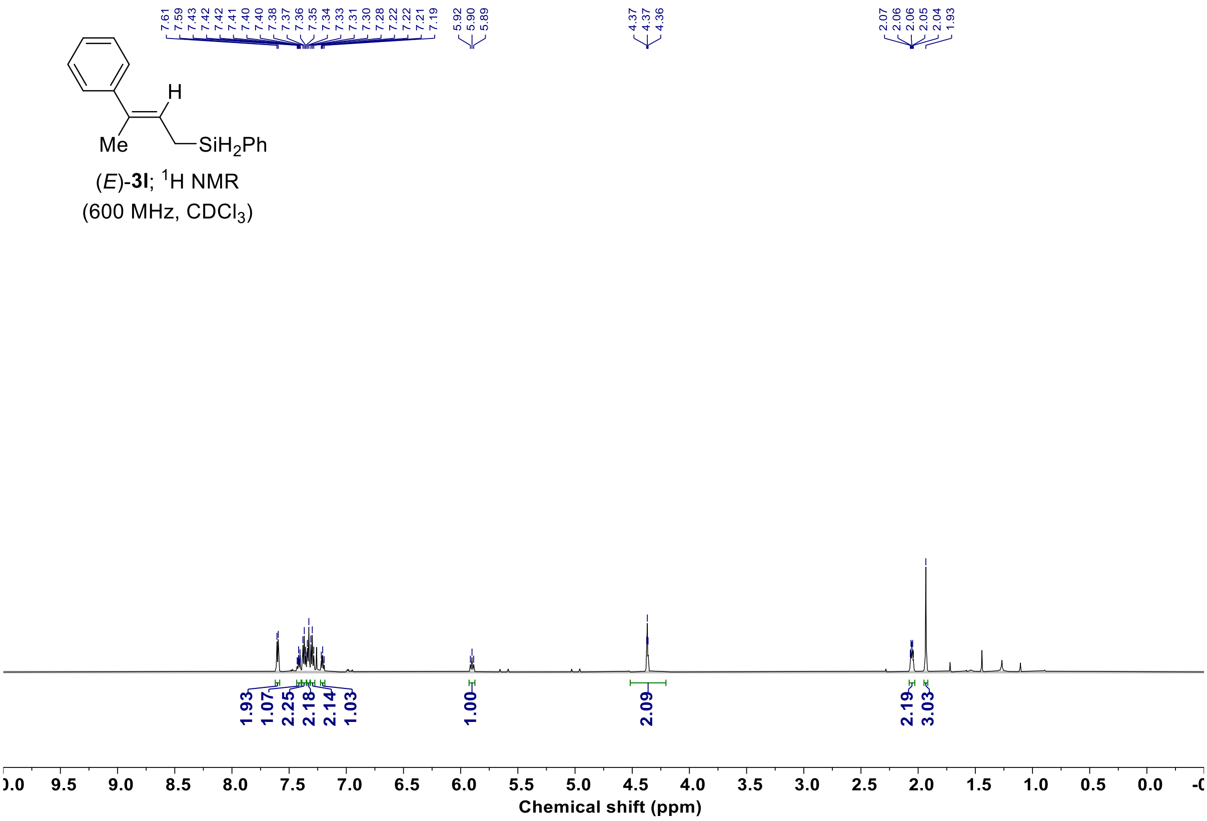

(*E*)-**3l**; ^{1}H NMR
(600 MHz, $CDCl_3$)

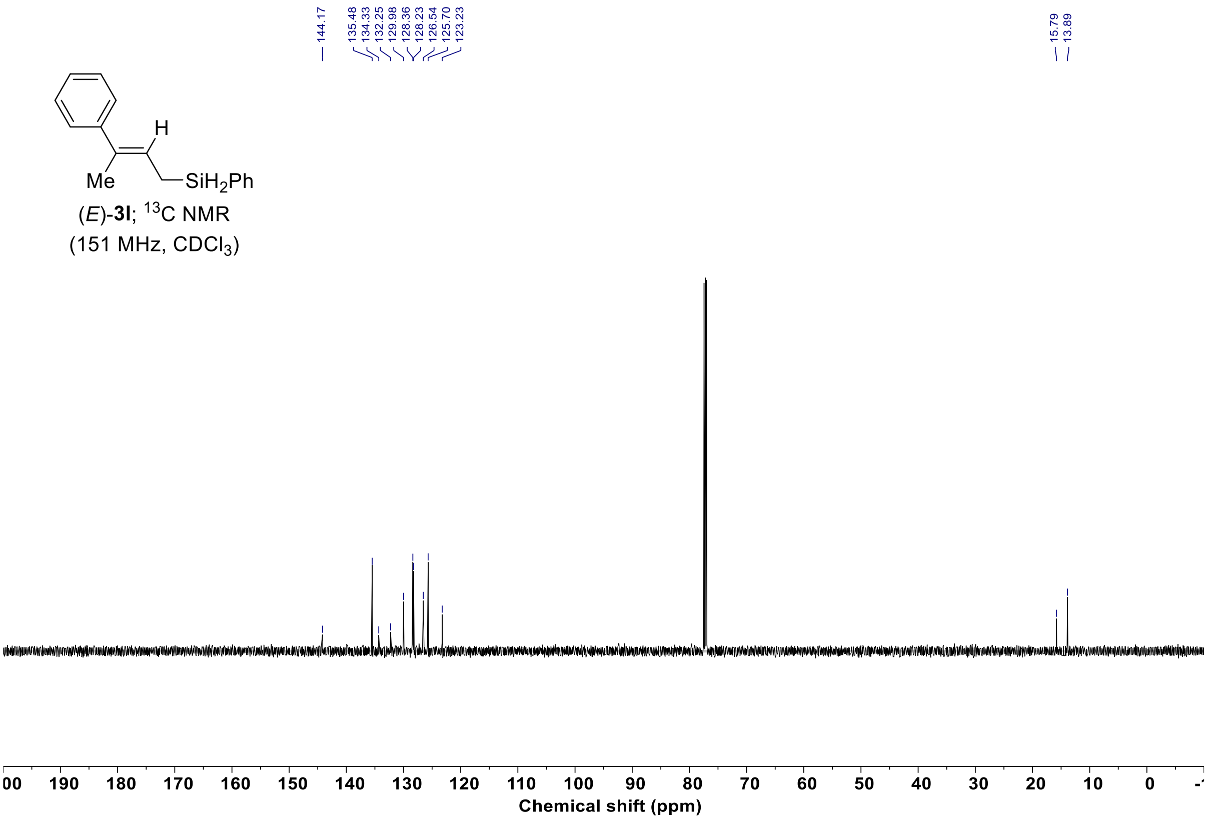

(*E*)-**3l**; ^{13}C NMR
(151 MHz, $CDCl_3$)

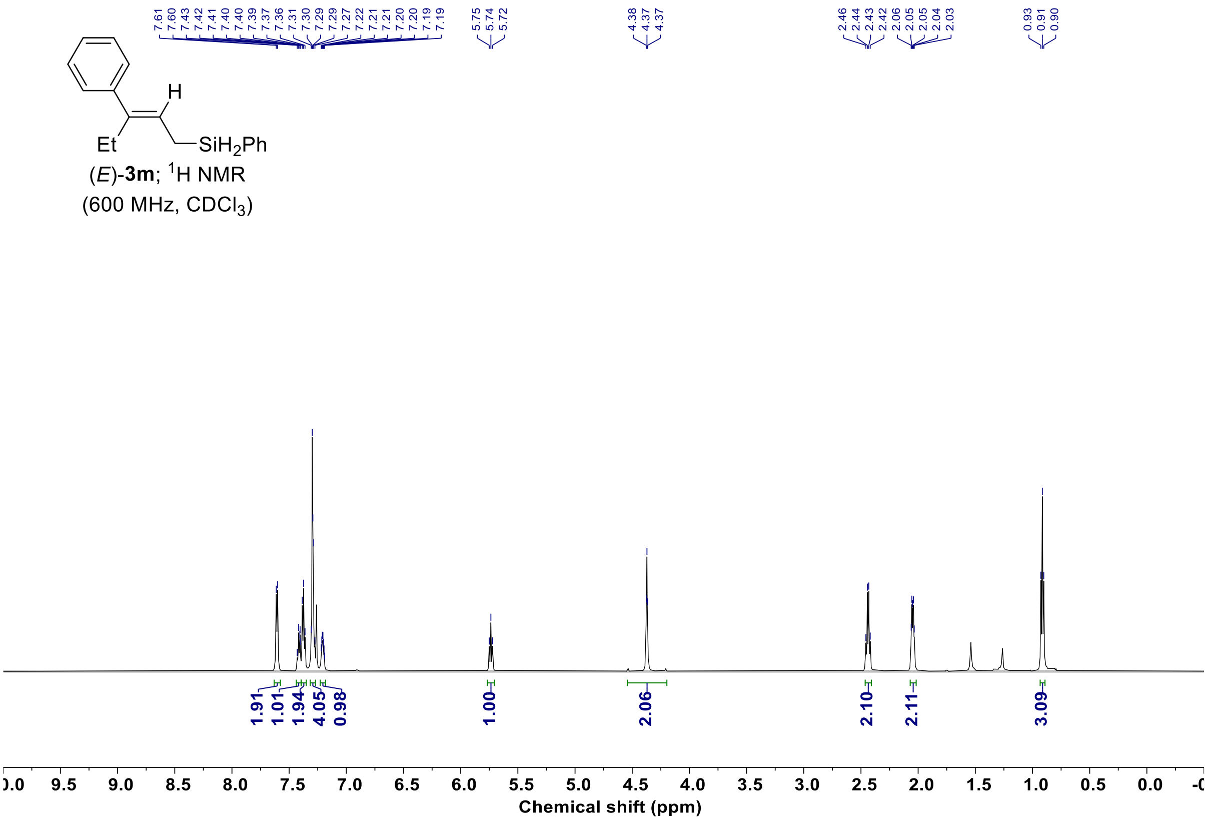

(*E*)-**3m**; ^{1}H NMR
(600 MHz, $CDCl_3$)

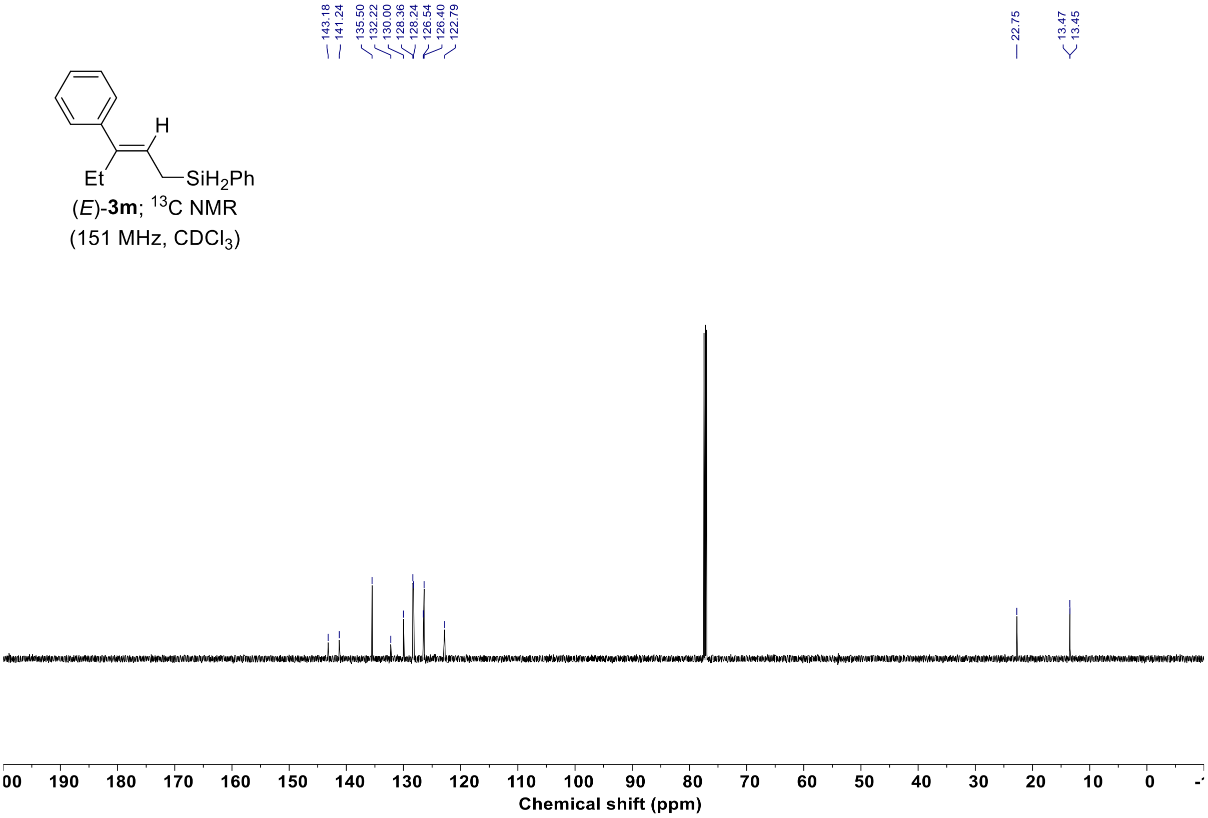

(*E*)-**3m**; ^{13}C NMR
(151 MHz, $CDCl_3$)

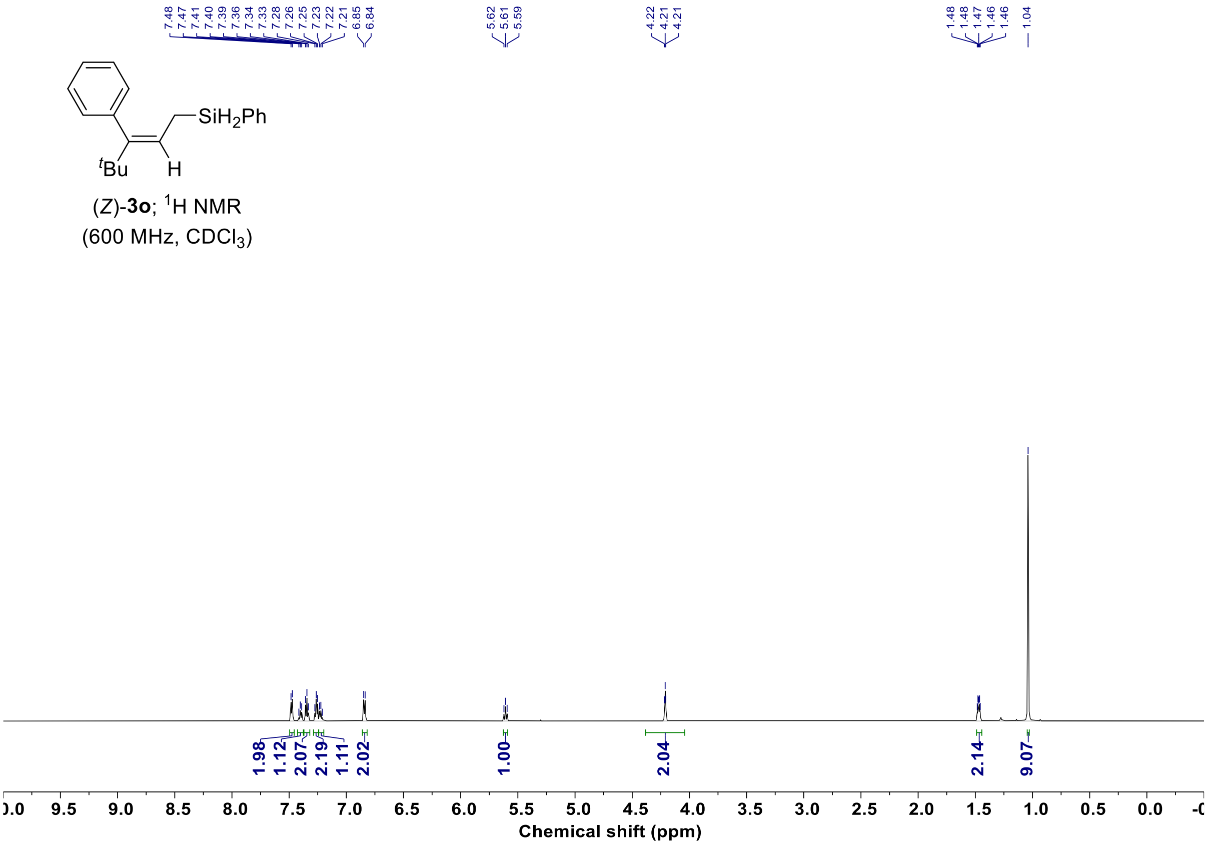
SiH2Ph
tBu H
(Z)-3o; 1H NMR
(600 MHz, CDCl3)
Chemical shift (ppm)

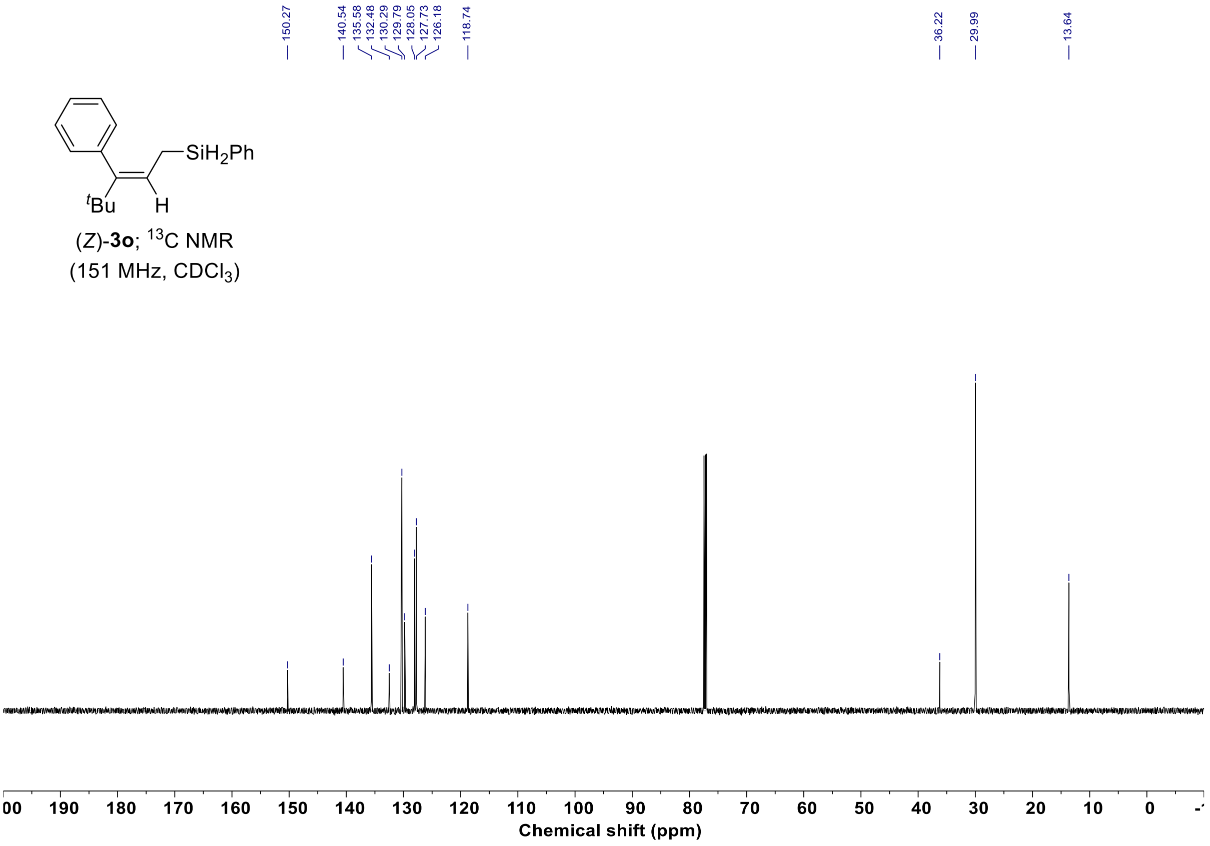
SiH2Ph
tBu H
(Z)-3o; 13C NMR
(151 MHz, CDCl3)
Chemical shift (ppm)

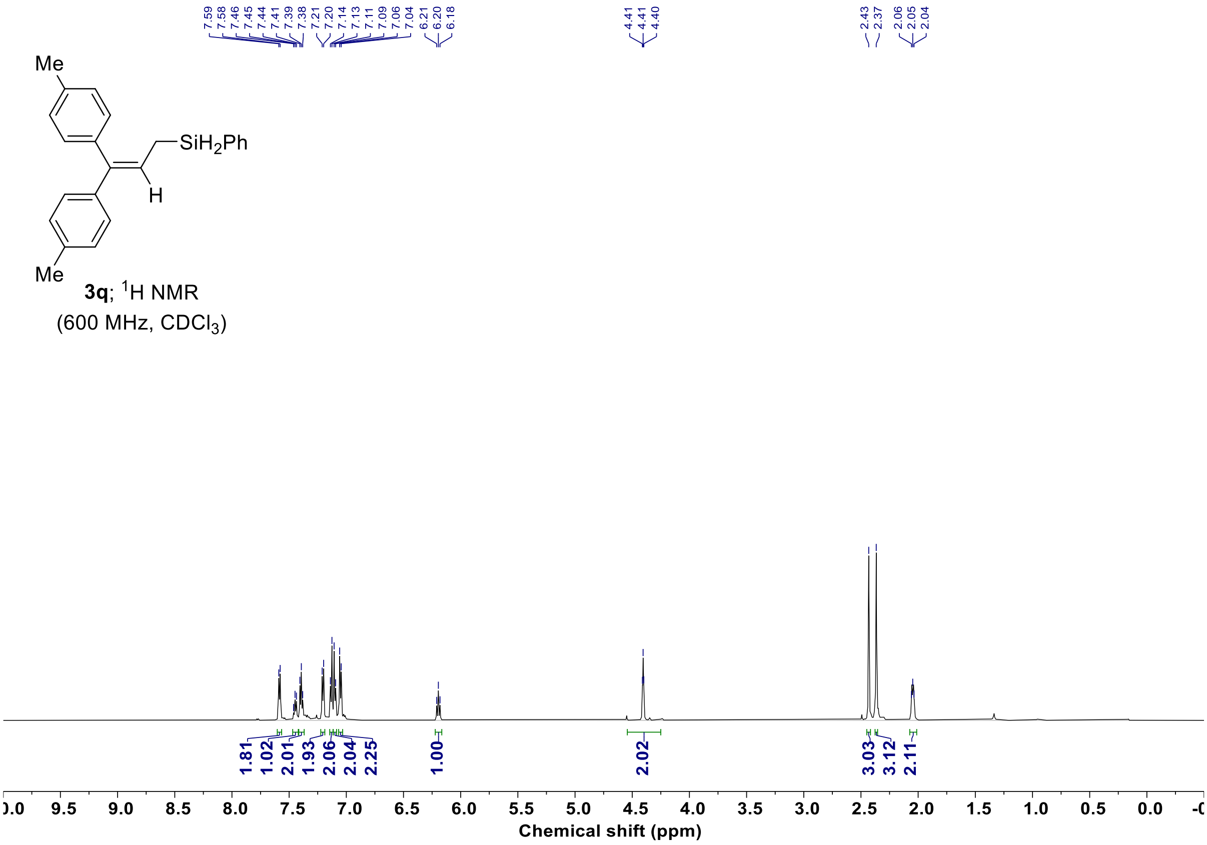
Me
SiH$_2$Ph
H
Me
3q; ^{1}H NMR
(600 MHz, CDCl$_3$)
Chemical shift (ppm)

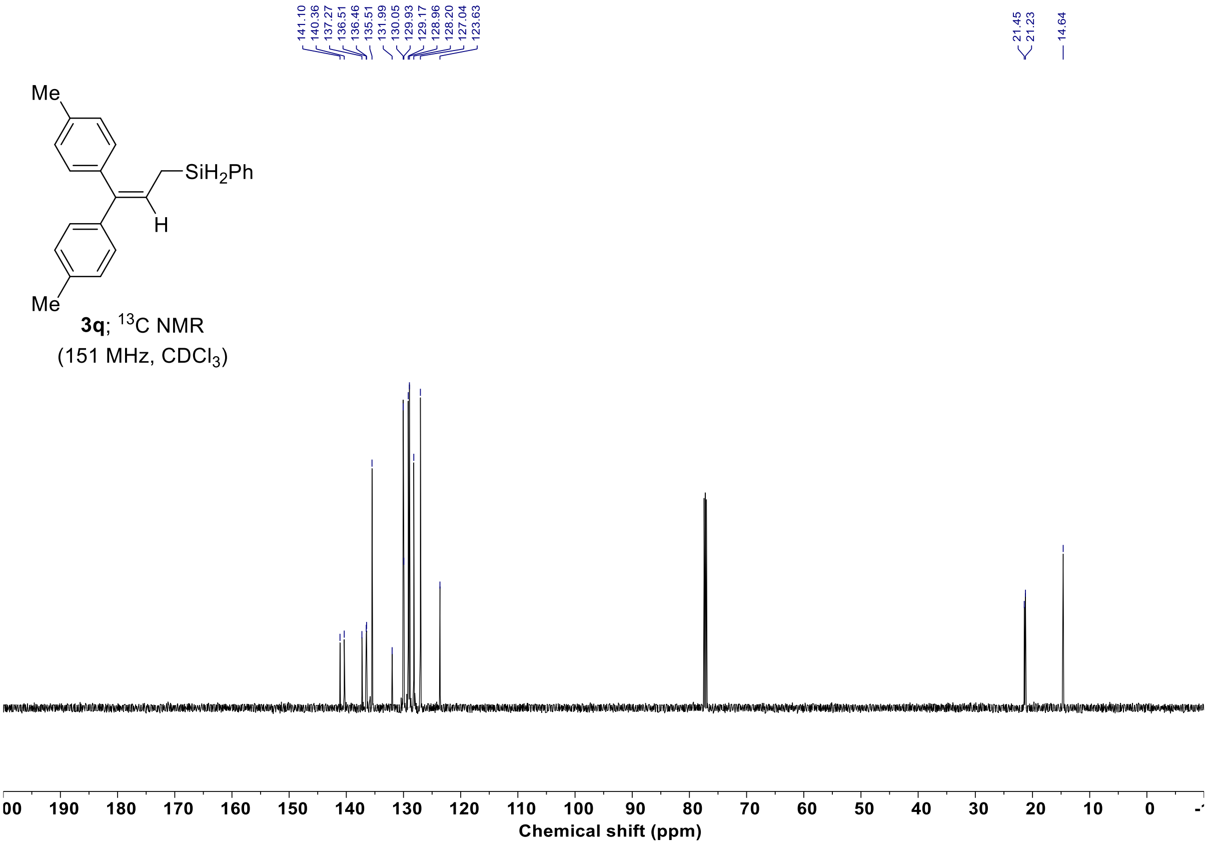
Me
SiH$_2$Ph
H
Me
3q; ^{13}C NMR
(151 MHz, CDCl$_3$)
Chemical shift (ppm)

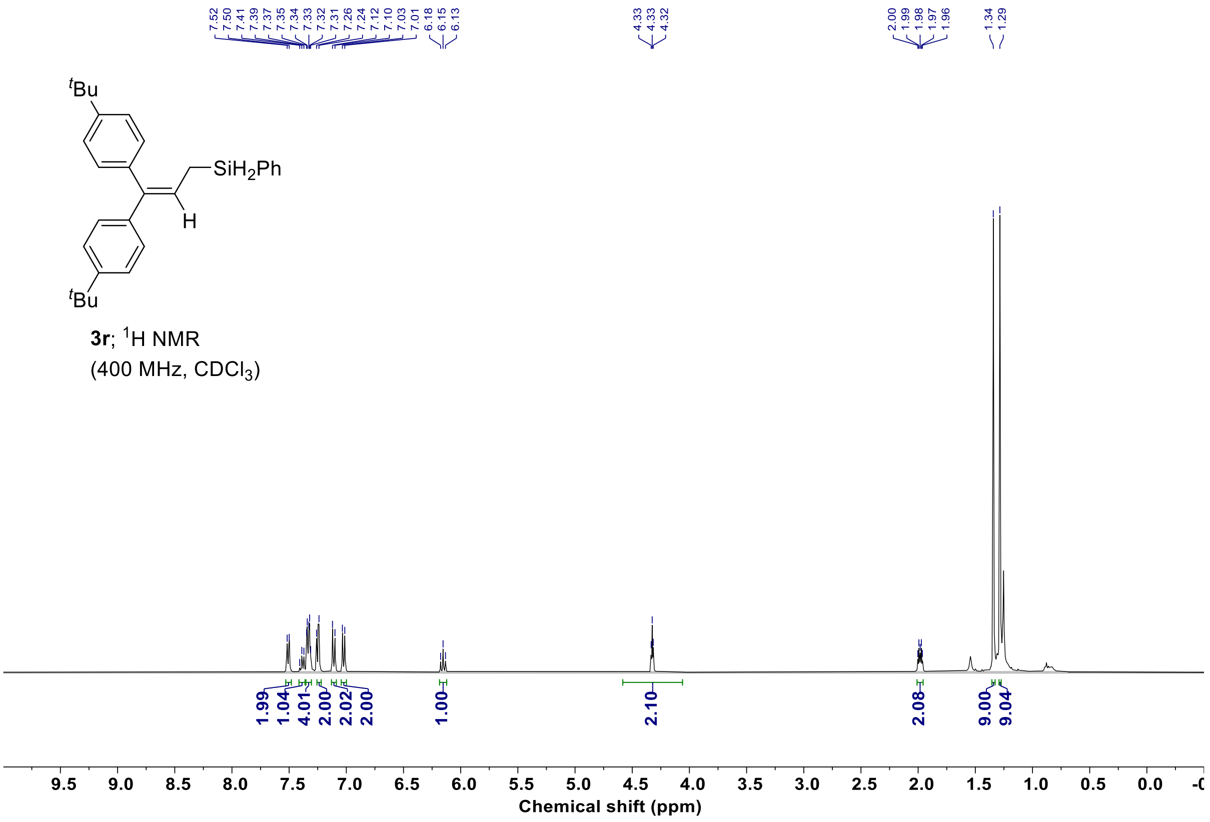

3r; ^{1}H NMR
(400 MHz, $CDCl_3$)
Chemical shift (ppm)

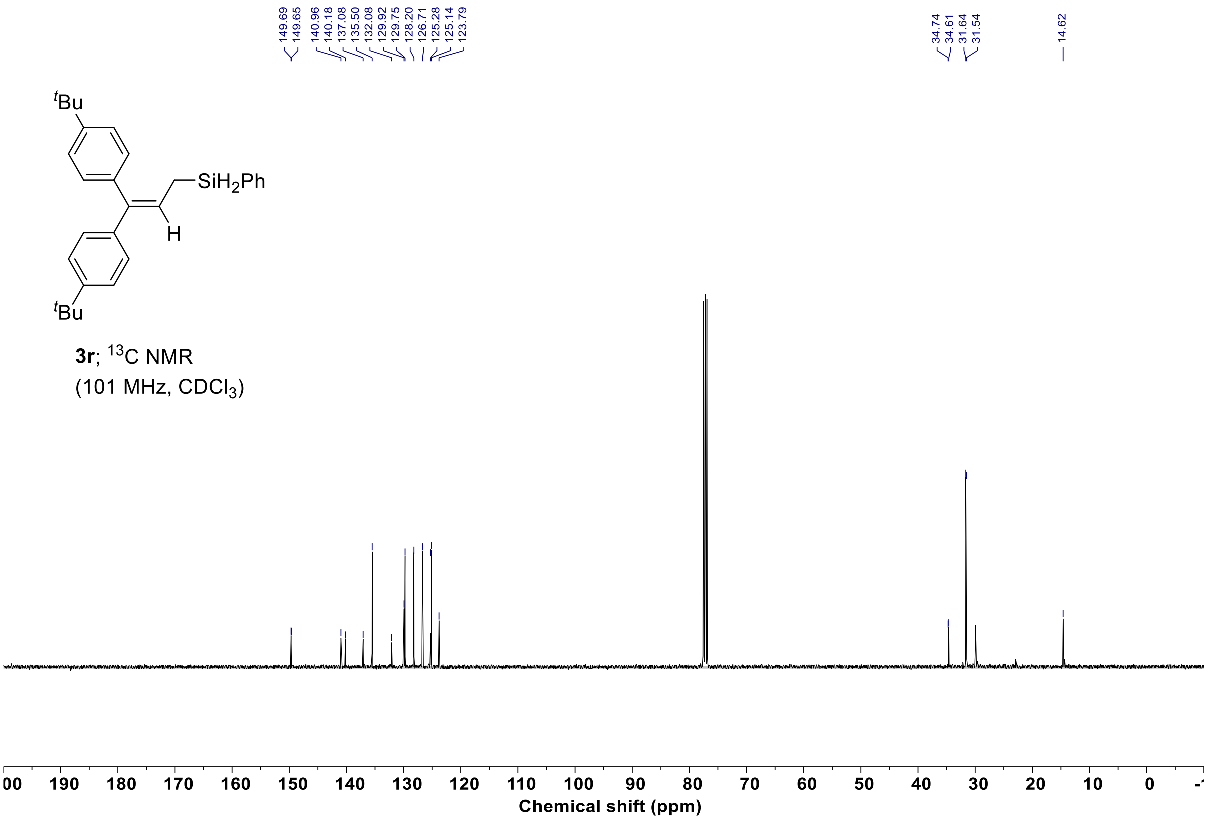

3r; ^{13}C NMR
(101 MHz, $CDCl_3$)
Chemical shift (ppm)

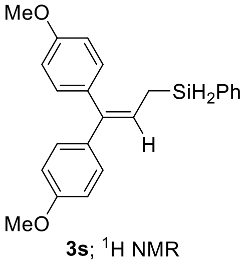

3s; ^{1}H NMR
(600 MHz, $CDCl_3$)

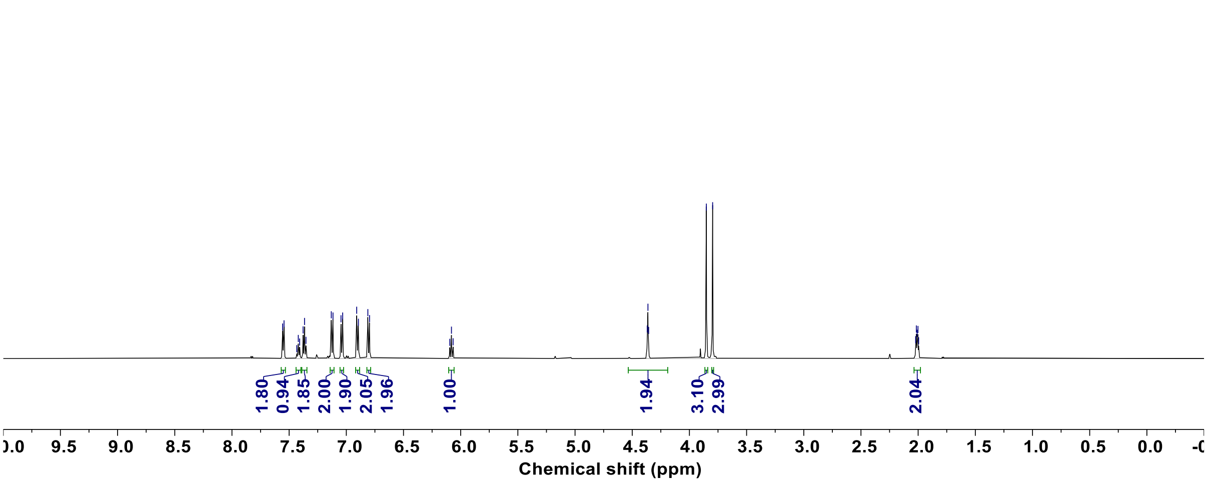

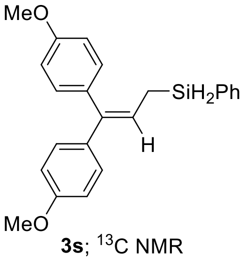

3s; ^{13}C NMR
(151 MHz, $CDCl_3$)

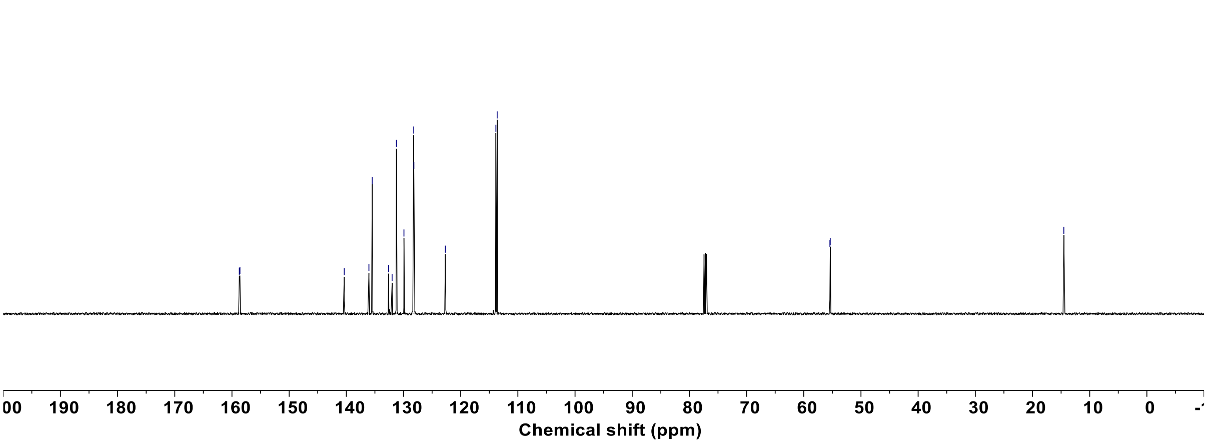

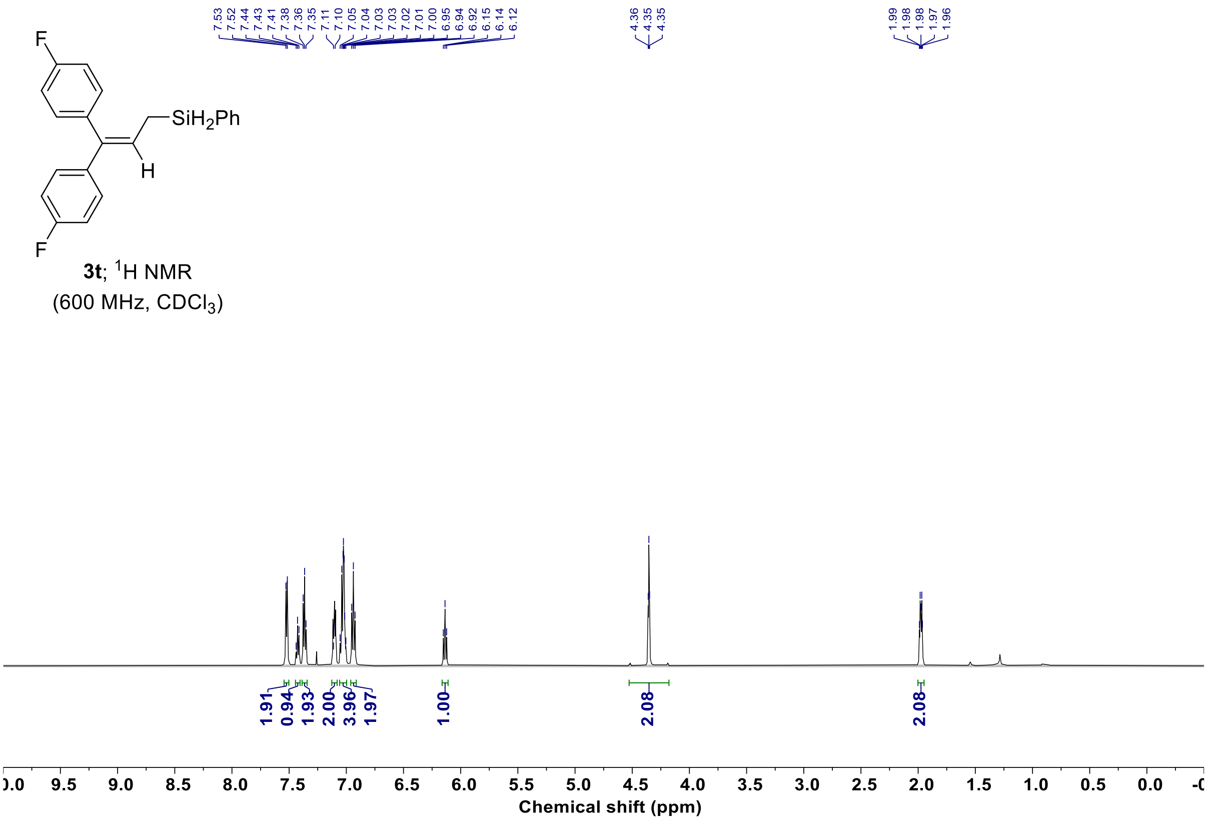

3t; ^{1}H NMR
(600 MHz, $CDCl_3$)

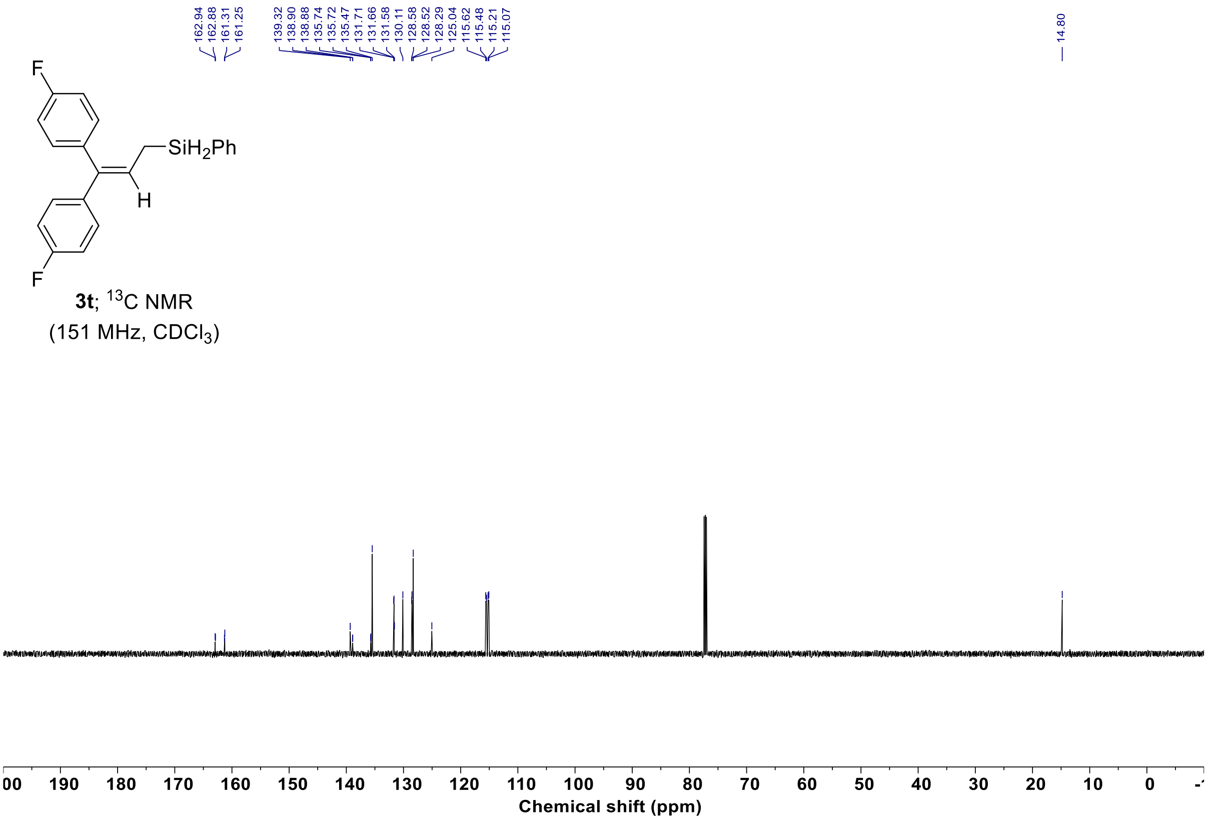

3t; ^{13}C NMR
(151 MHz, $CDCl_3$)

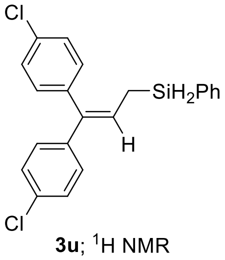

3u; ^{1}H NMR
(600 MHz, $CDCl_3$)

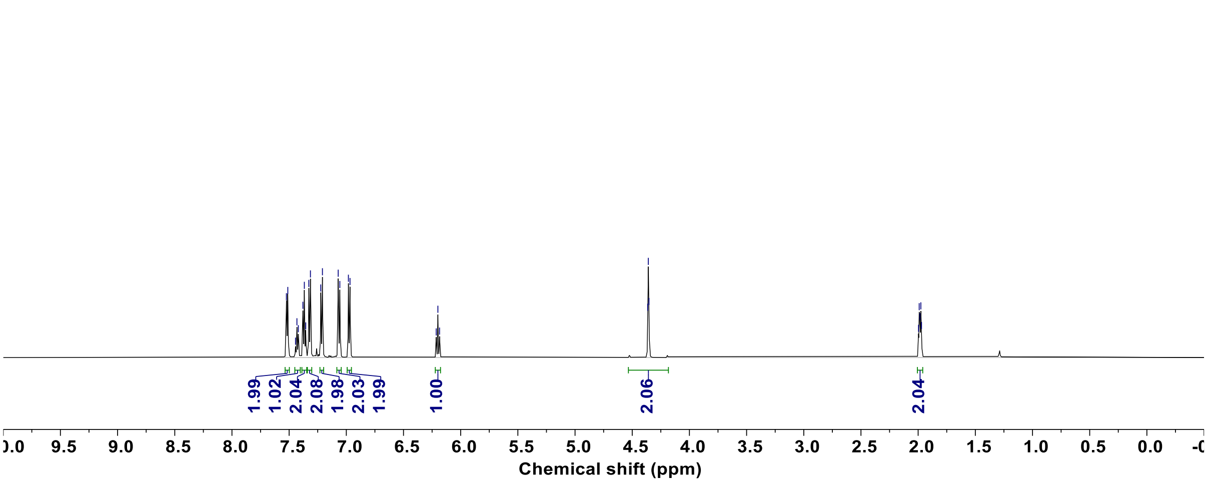

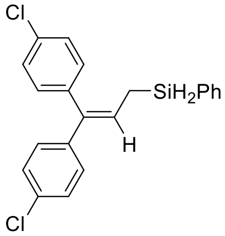

3u; ^{13}C NMR
(151 MHz, $CDCl_3$)

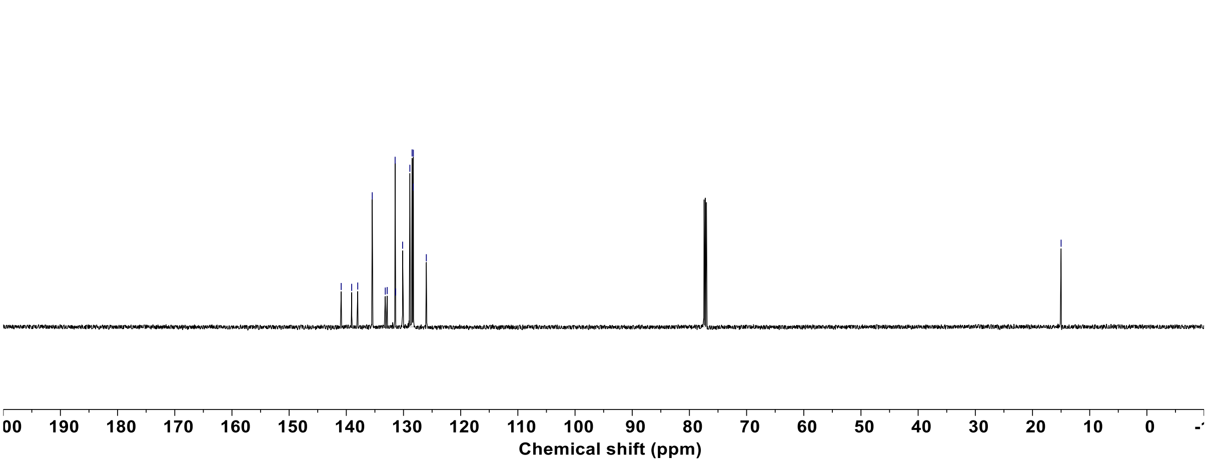

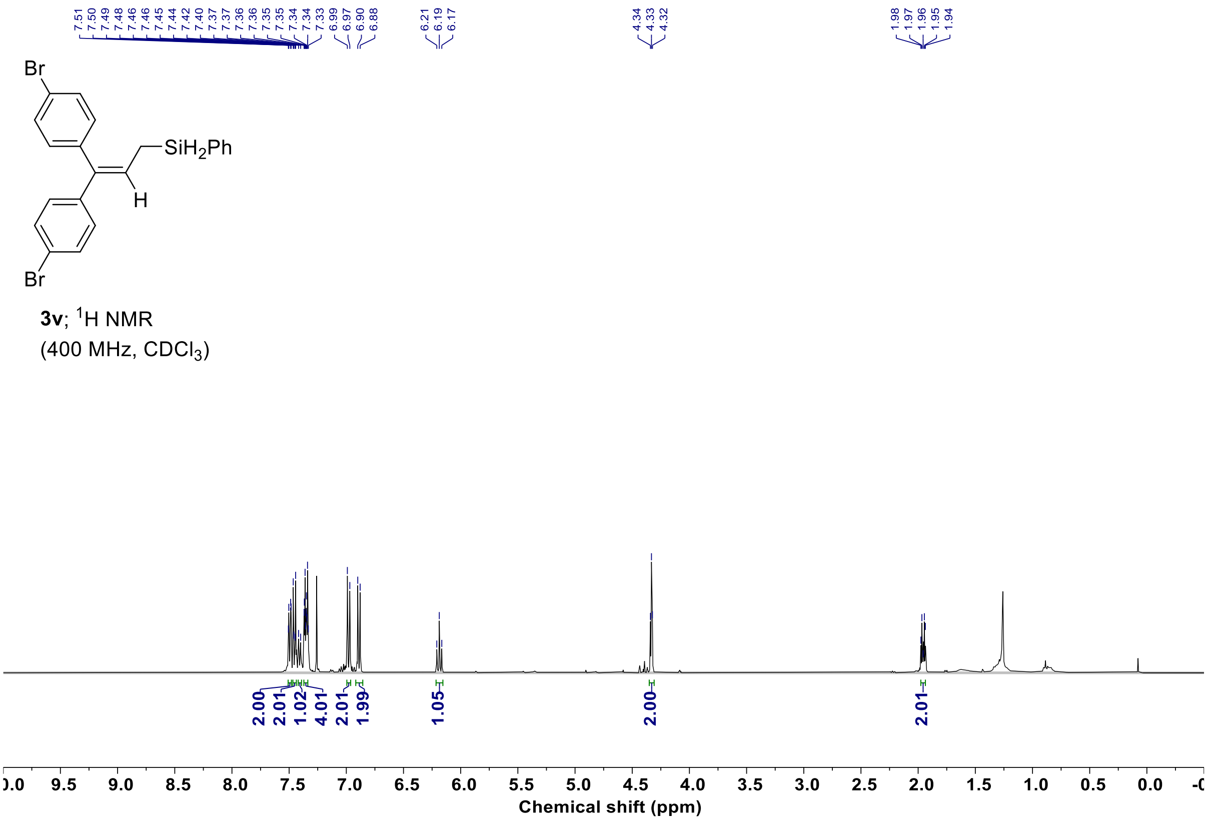

3v; ^{1}H NMR
(400 MHz, $CDCl_3$)

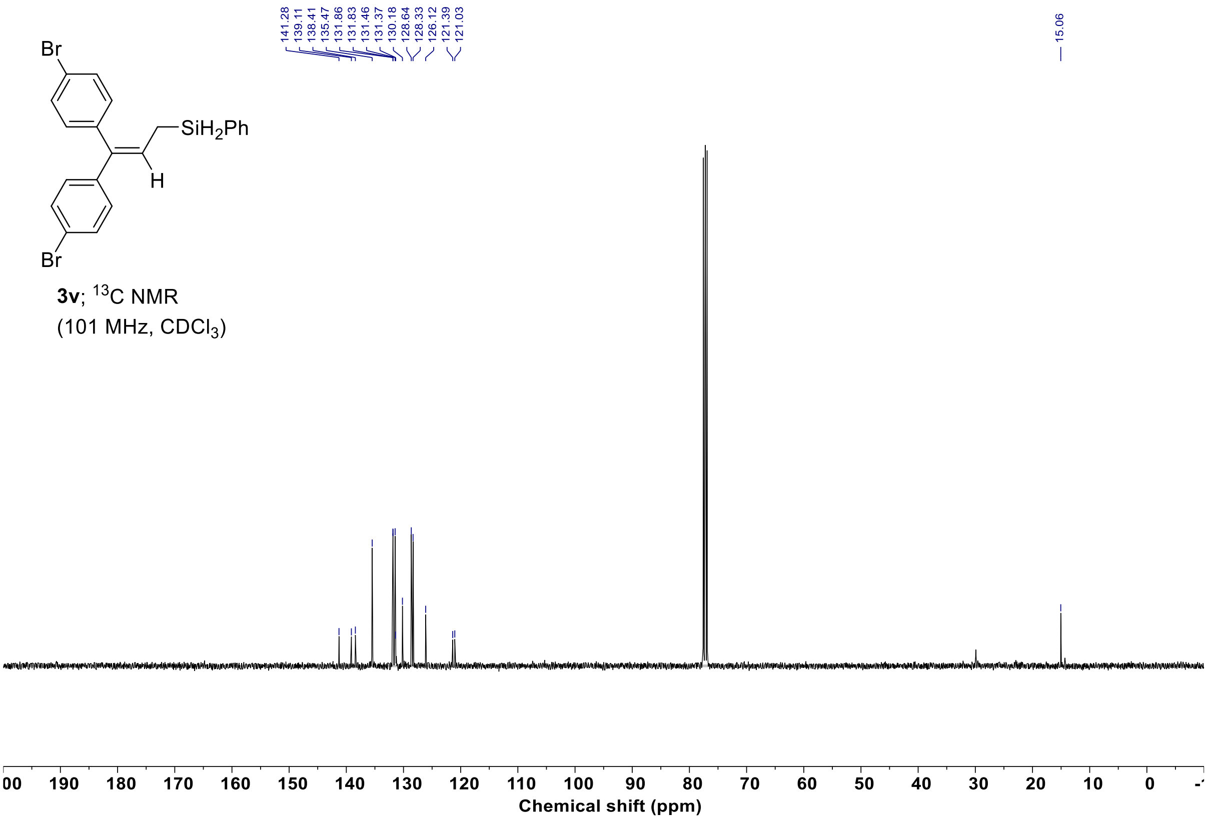

3v; ^{13}C NMR
(101 MHz, $CDCl_3$)

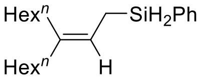

3w; ^{1}H NMR
(400 MHz, $CDCl_3$)

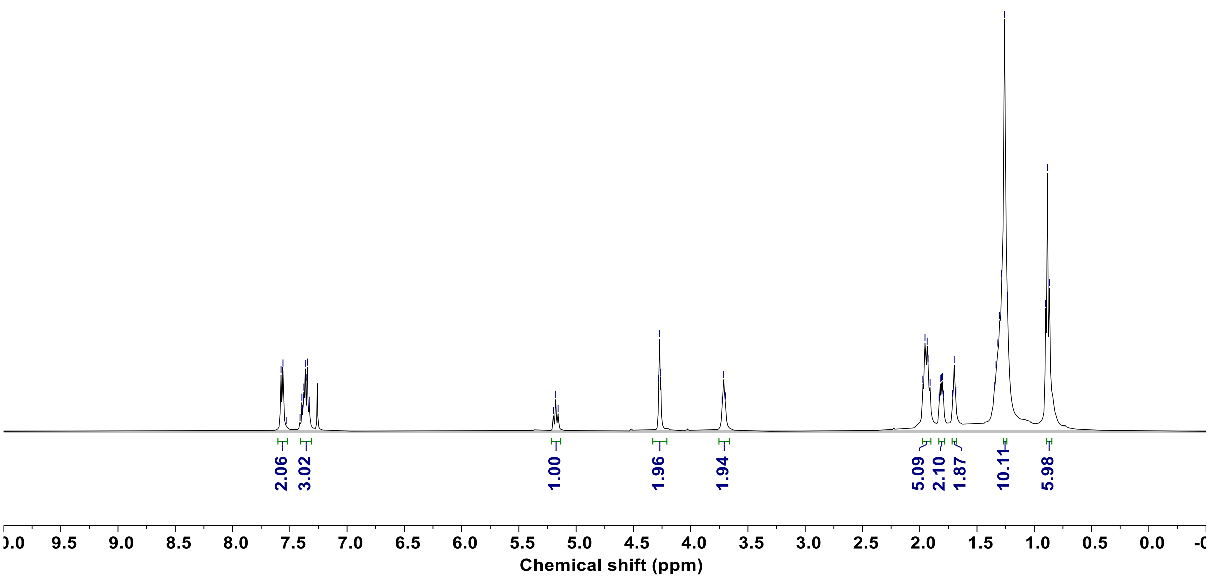

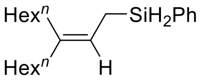

3w; ^{13}C NMR
(101 MHz, $CDCl_3$)

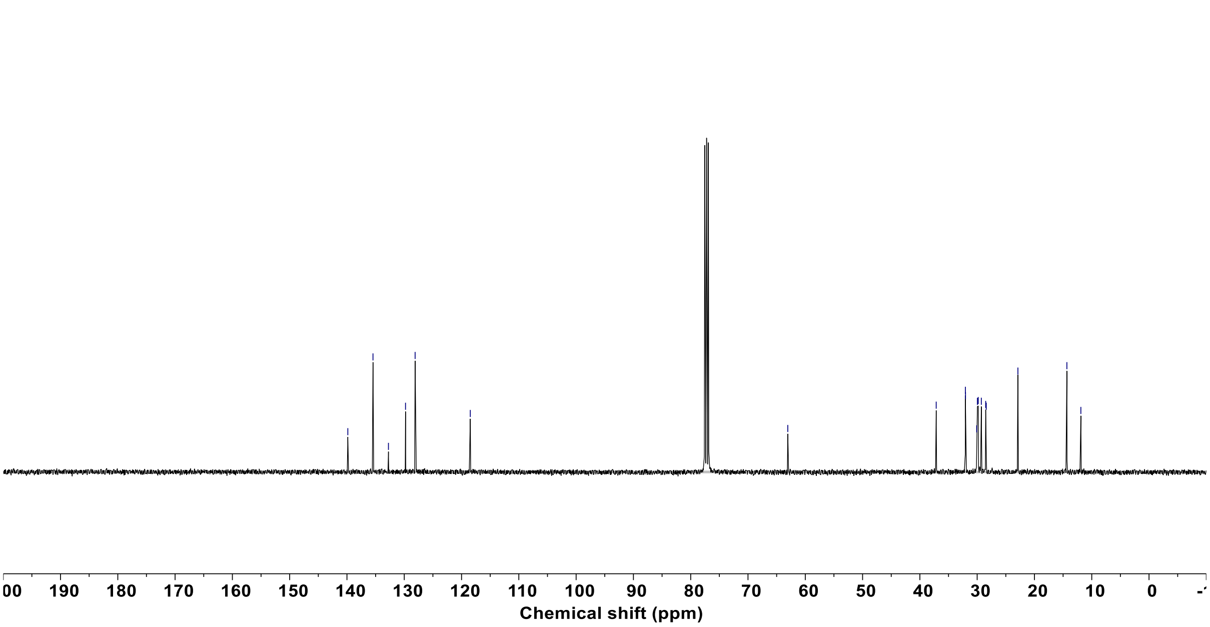

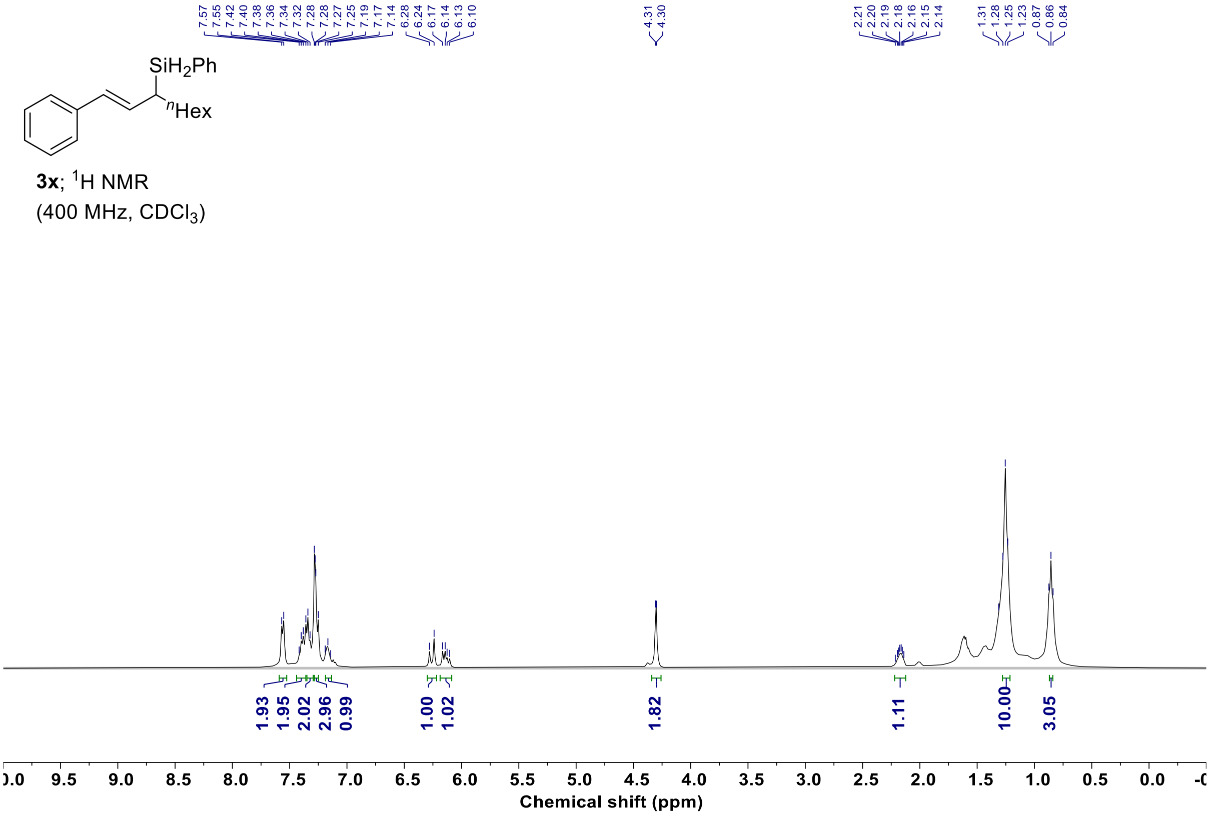

3x; ^{1}H NMR
(400 MHz, $CDCl_3$)

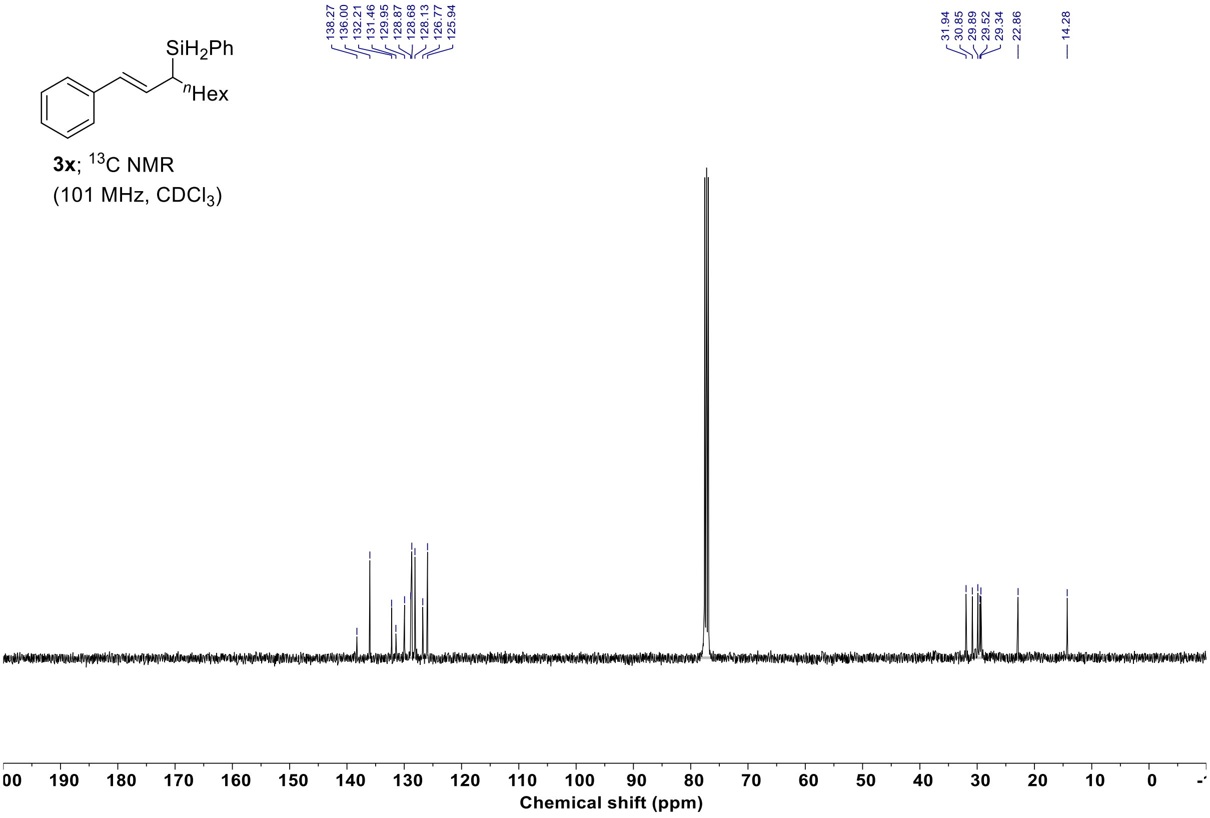

3x; ^{13}C NMR
(101 MHz, $CDCl_3$)

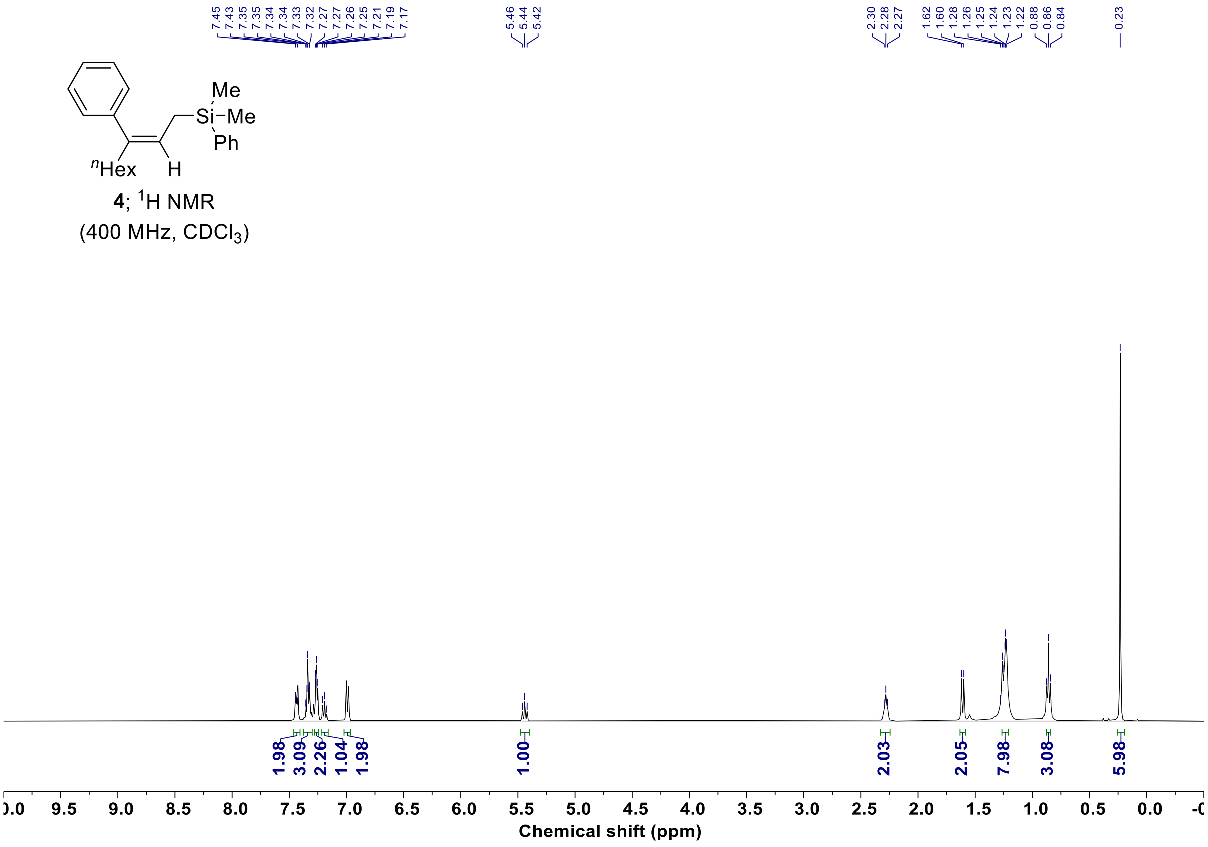
Me
Si–Me
Ph
ⁿHex
H
4; ¹H NMR
(400 MHz, CDCl₃)
Chemical shift (ppm)

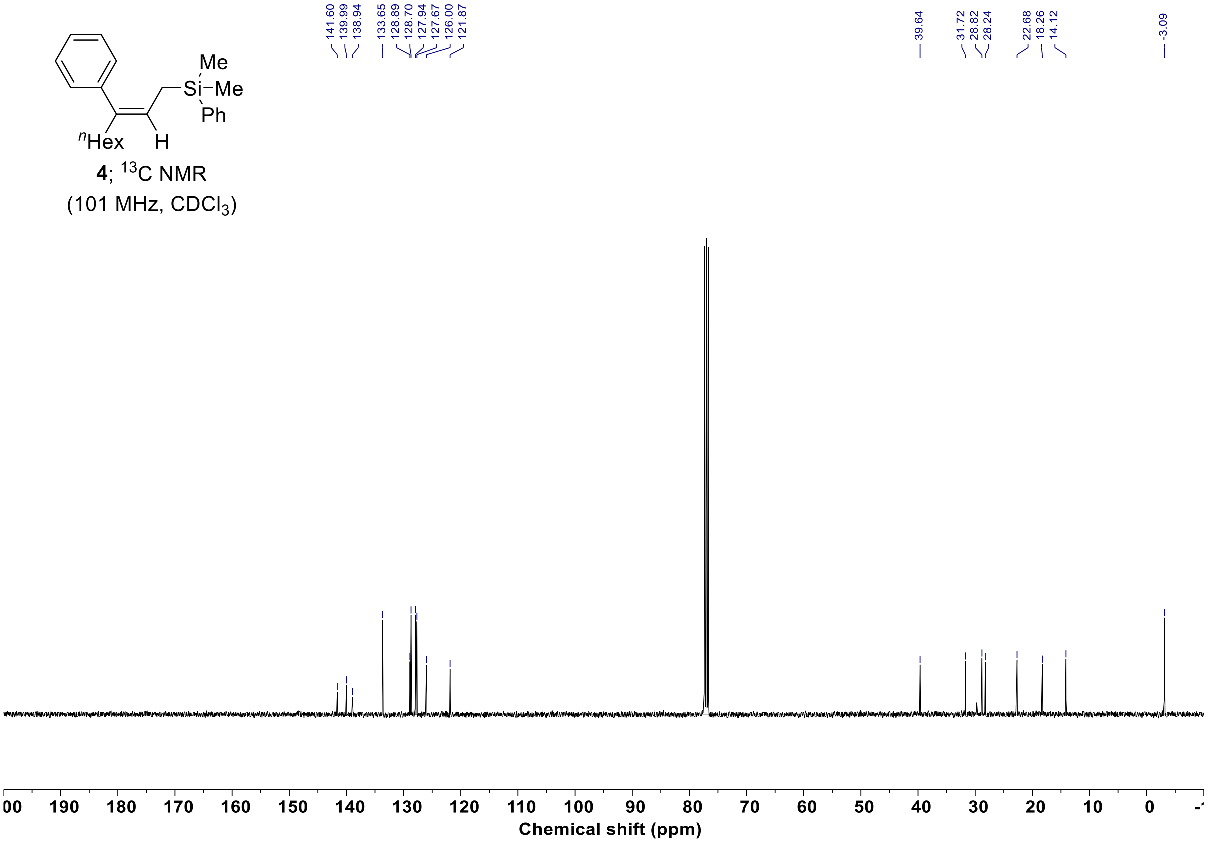
Me
Si–Me
Ph
ⁿHex
H
4; ¹³C NMR
(101 MHz, CDCl₃)
Chemical shift (ppm)

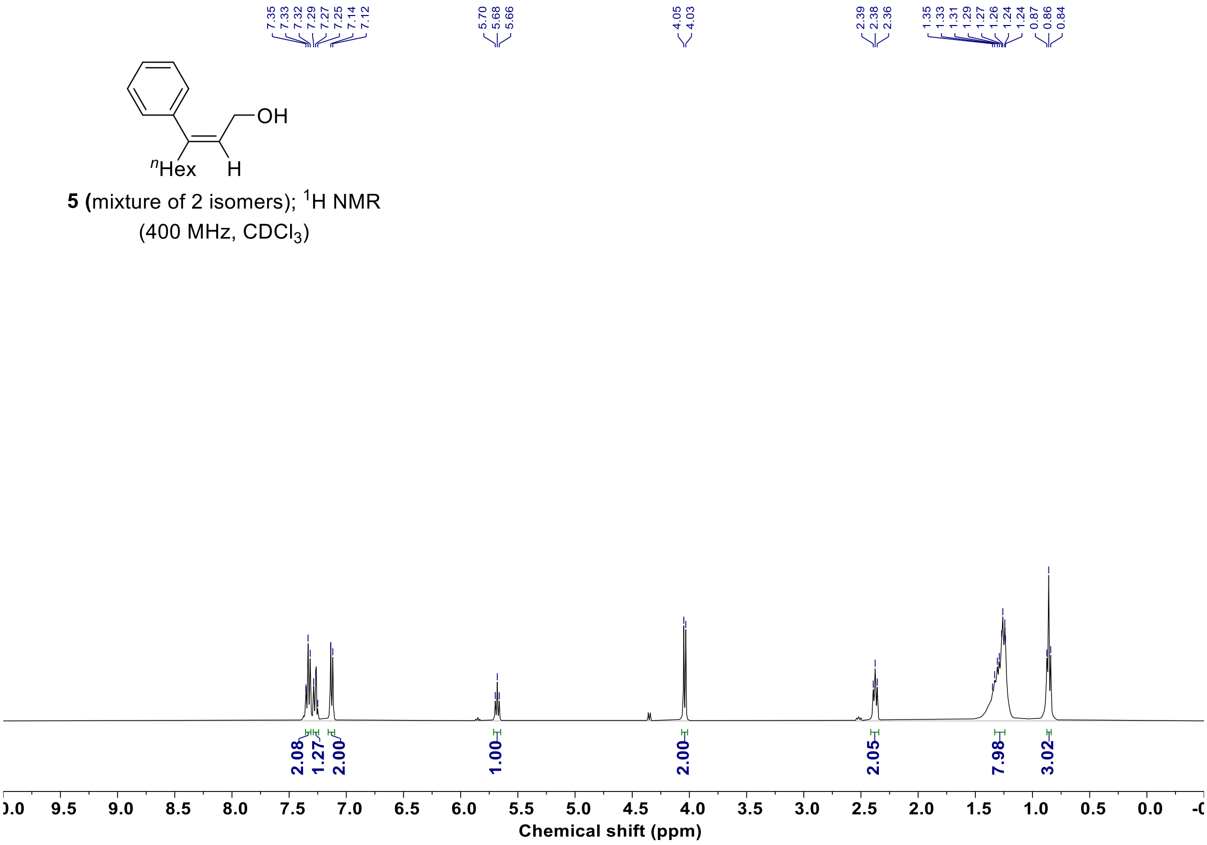
OH
ⁿHex H
5 (mixture of 2 isomers); 1H NMR
(400 MHz, CDCl3)
7.35 7.33 7.32 7.29 7.27 7.25 7.14 7.12
5.70 5.68 5.66
4.05 4.03
2.39 2.38 2.36
1.35 1.33 1.31 1.29 1.27 1.26 1.24 1.24 0.87 0.86 0.84
2.08 1.27 2.00 1.00 2.00 2.05 7.98 3.02
Chemical shift (ppm)

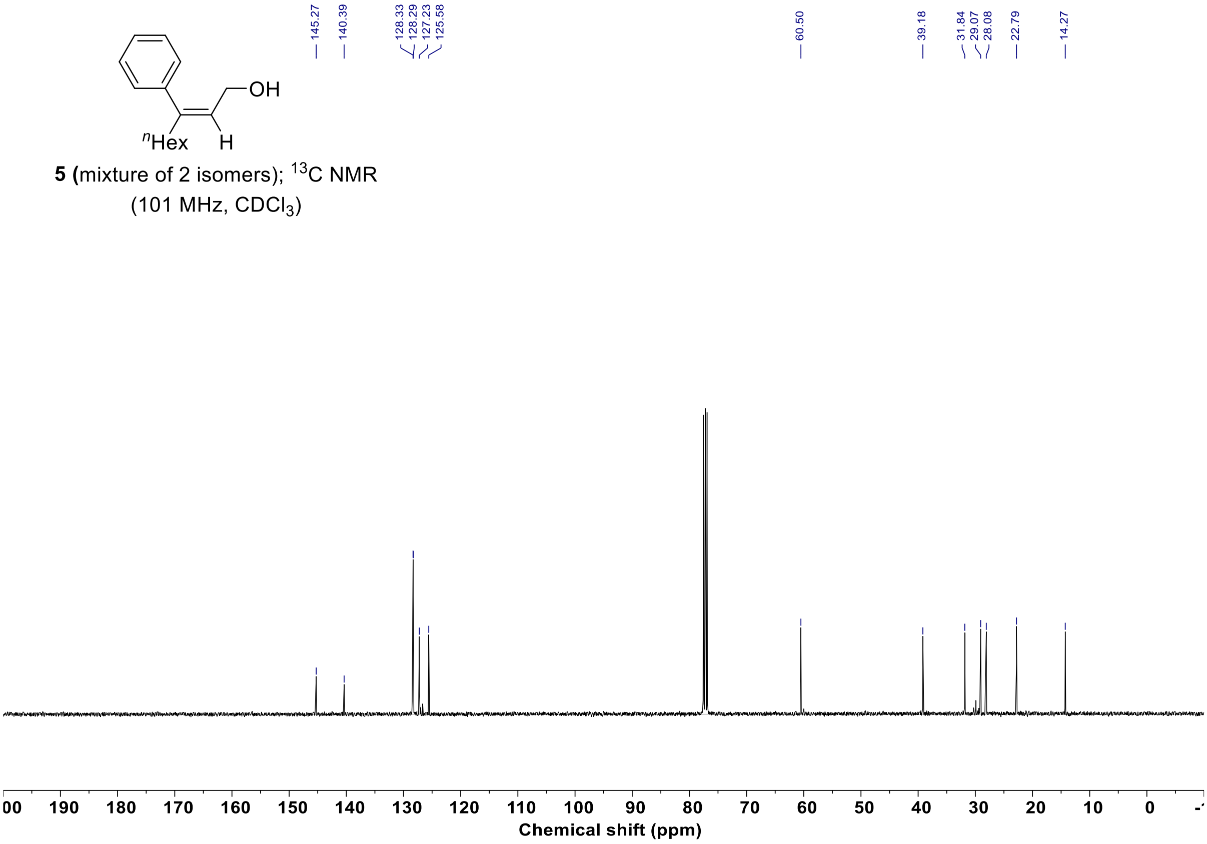
OH
ⁿHex H
5 (mixture of 2 isomers); 13C NMR
(101 MHz, CDCl3)
145.27 140.39 128.33 128.29 127.23 125.58 60.50 39.18 31.84 29.07 28.08 22.79 14.27
Chemical shift (ppm)

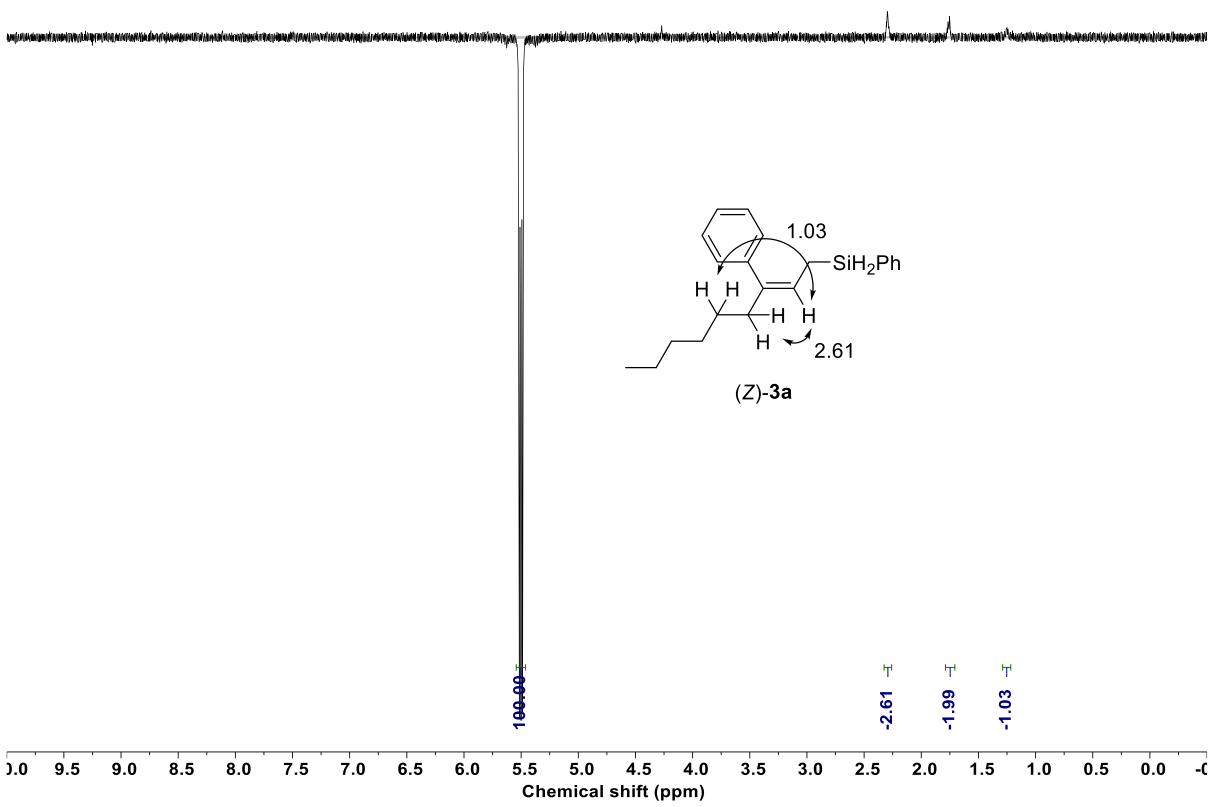
1.03
SiH2Ph
H
H
H
H
H
2.61
(Z)-3a
100.00
-2.61
-1.99
-1.03
Chemical shift (ppm)

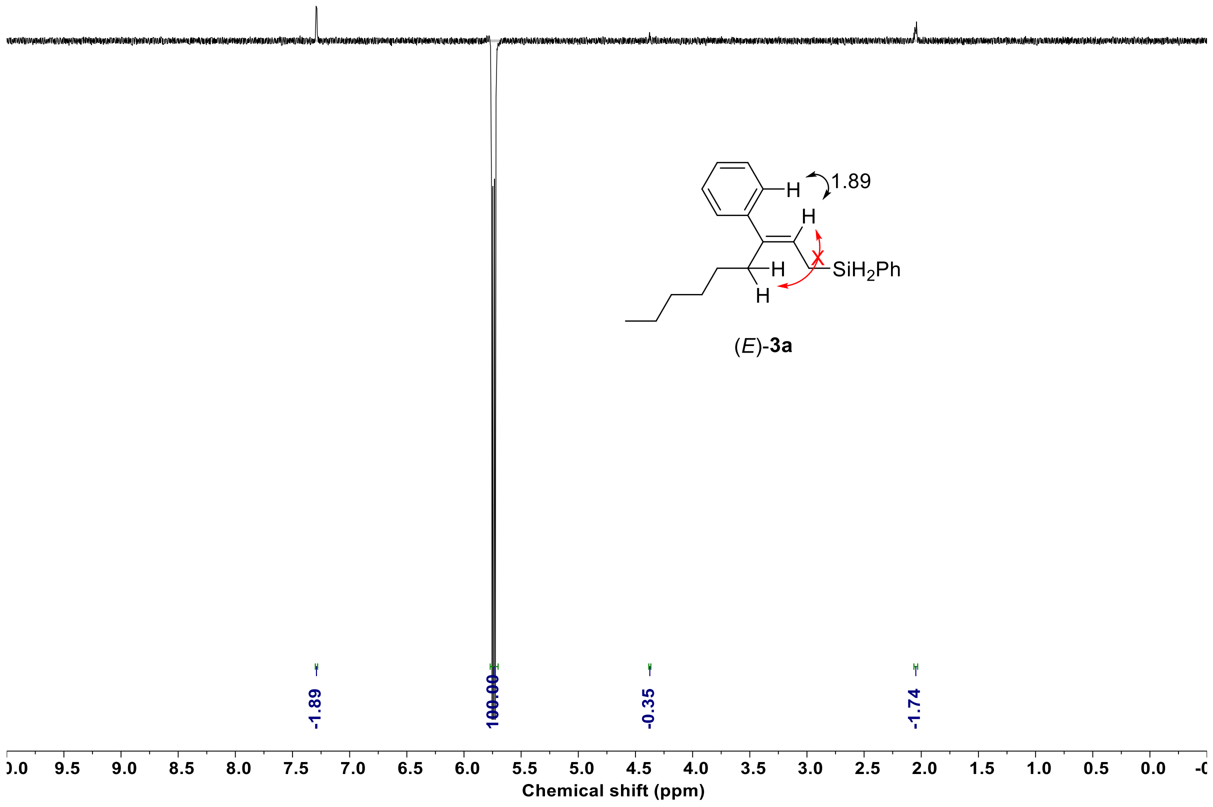
H
1.89
H
SiH2Ph
H
H
(E)-3a
-1.89
100.00
-0.35
-1.74
Chemical shift (ppm)

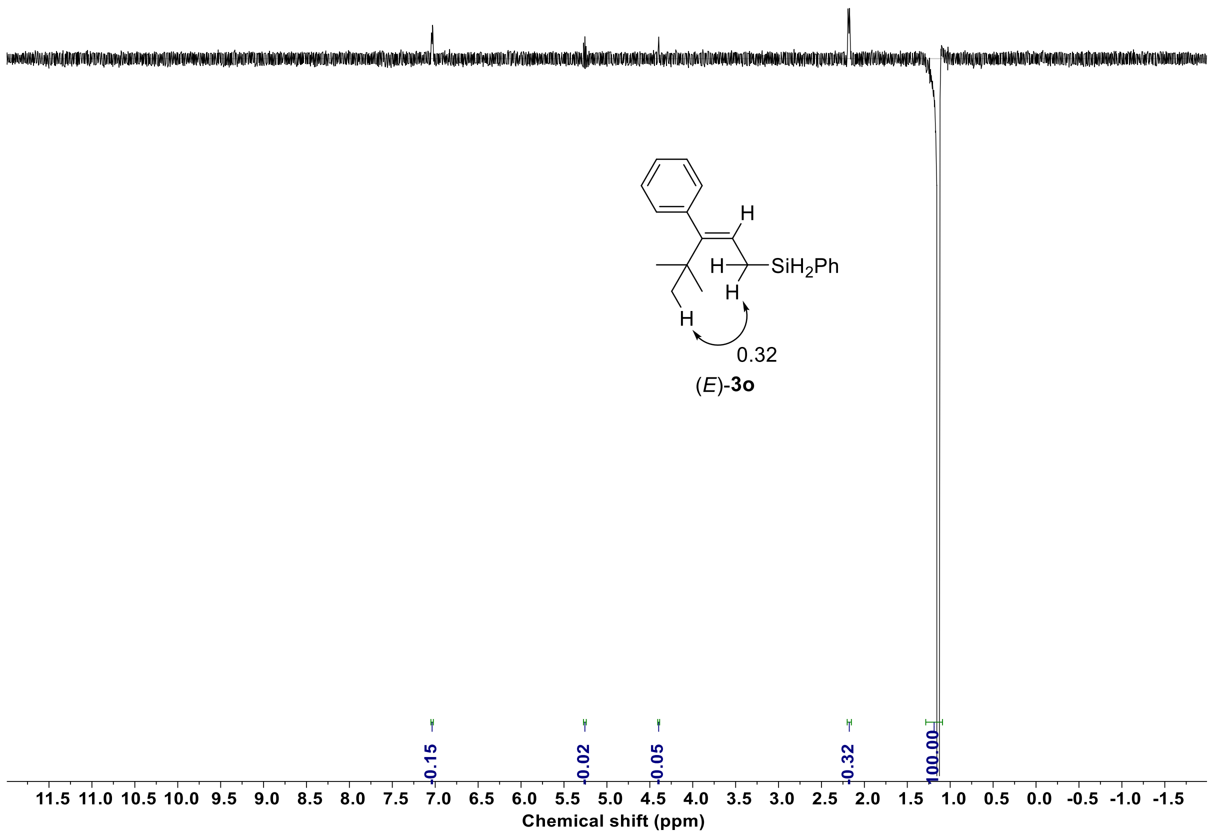
H
H
SiH2Ph
H
H
0.32
(E)-3o
-0.15
-0.02
-0.05
-0.32
100.00
11.5 11.0 10.5 10.0 9.5 9.0 8.5 8.0 7.5 7.0 6.5 6.0 5.5 5.0 4.5 4.0 3.5 3.0 2.5 2.0 1.5 1.0 0.5 0.0 -0.5 -1.0 -1.5
Chemical shift (ppm)

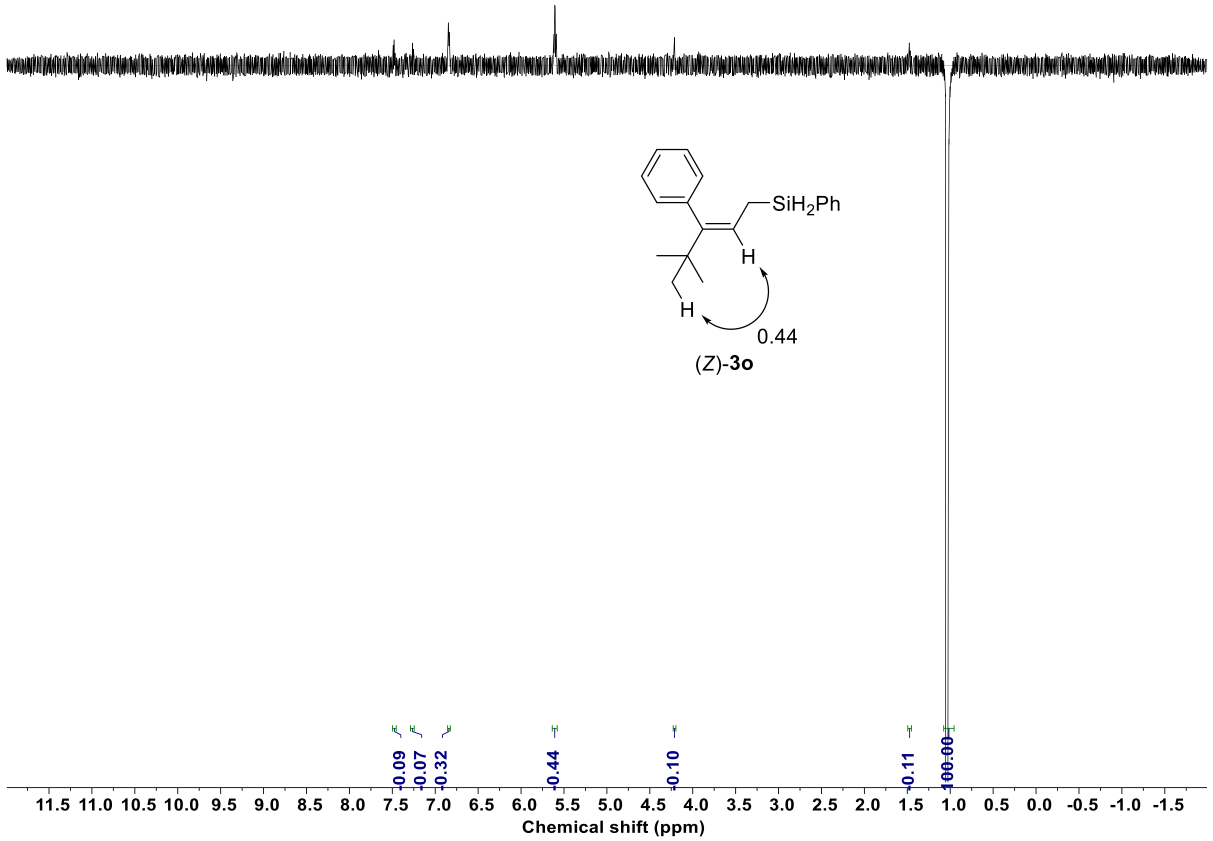
SiH2Ph
H
H
0.44
(Z)-3o
-0.09
-0.07
-0.32
-0.44
-0.10
-0.11
100.00
11.5 11.0 10.5 10.0 9.5 9.0 8.5 8.0 7.5 7.0 6.5 6.0 5.5 5.0 4.5 4.0 3.5 3.0 2.5 2.0 1.5 1.0 0.5 0.0 -0.5 -1.0 -1.5
Chemical shift (ppm)

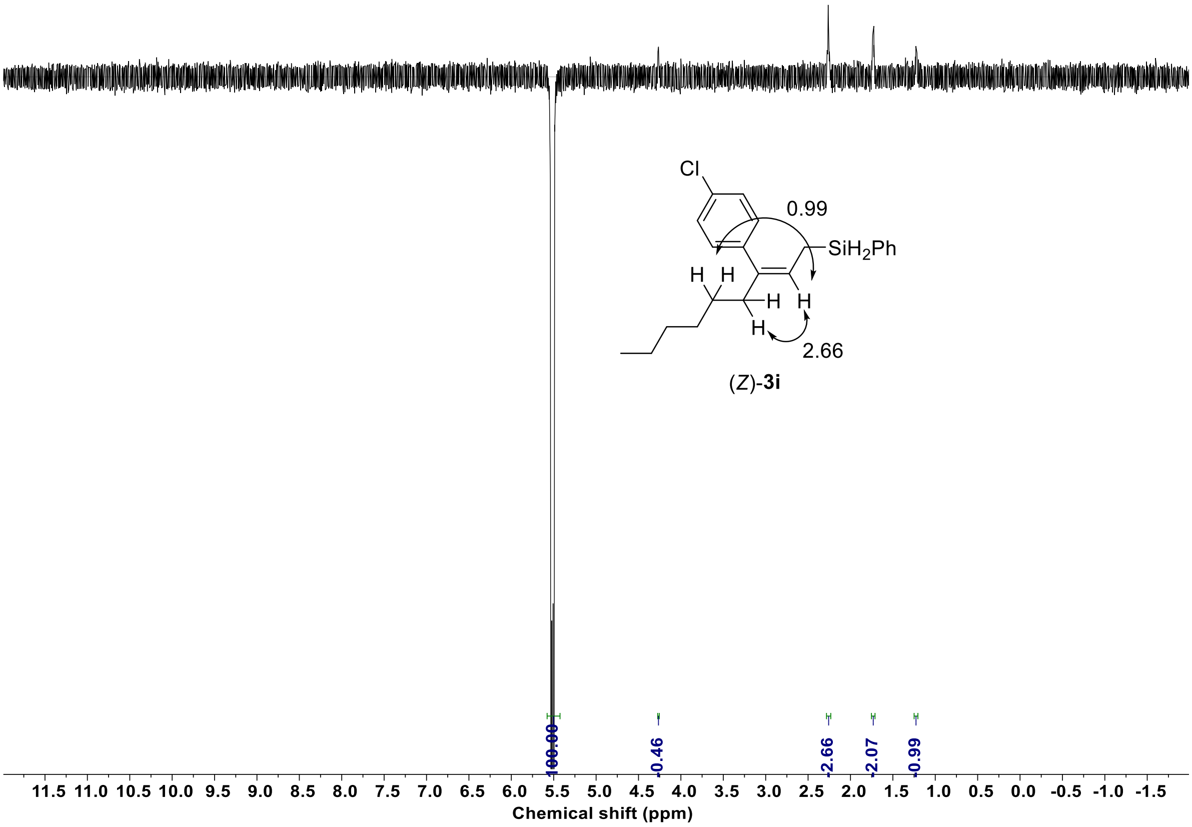
Cl
0.99
SiH2Ph
H H
H H
H
2.66
100.00
0.46
2.66
2.07
0.99
11.5 11.0 10.5 10.0 9.5 9.0 8.5 8.0 7.5 7.0 6.5 6.0 5.5 5.0 4.5 4.0 3.5 3.0 2.5 2.0 1.5 1.0 0.5 0.0 -0.5 -1.0 -1.5
Chemical shift (ppm)

(*Z*)-**3i**

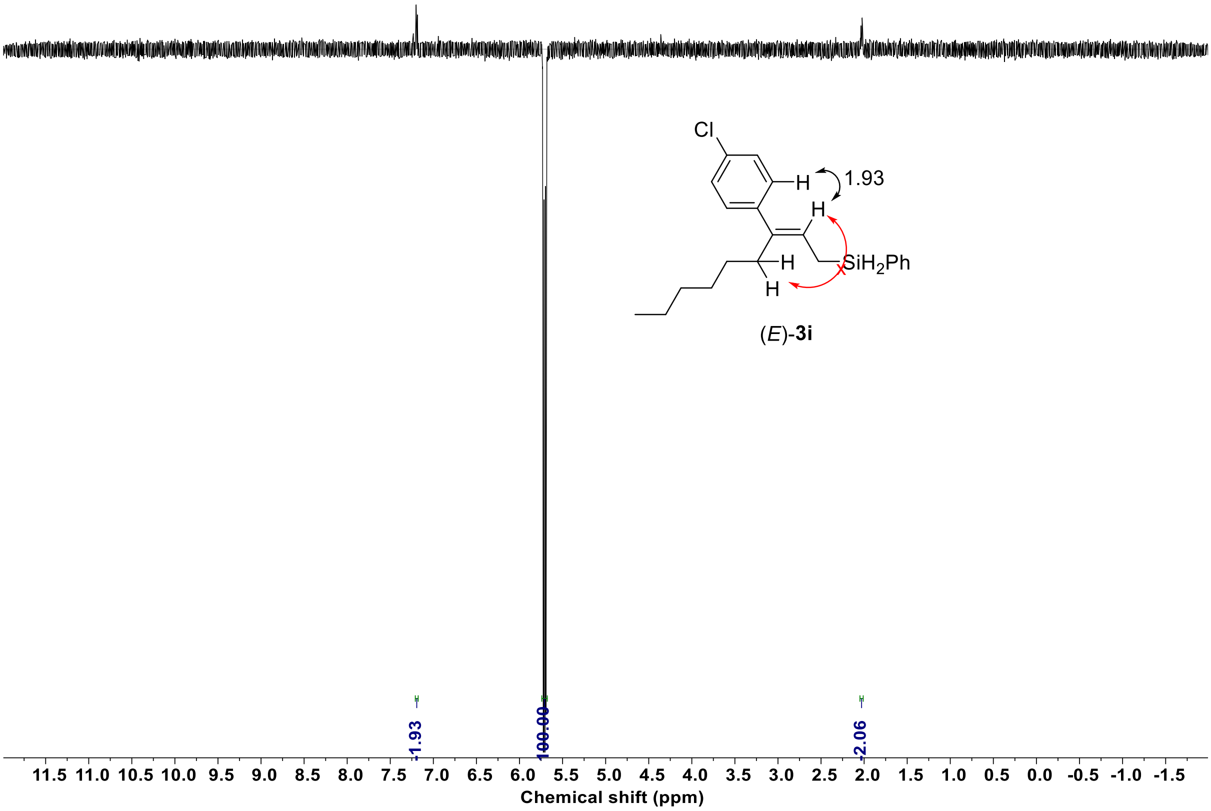
Cl
H
1.93
H
H
SiH2Ph
H
1.93
100.00
2.06
11.5 11.0 10.5 10.0 9.5 9.0 8.5 8.0 7.5 7.0 6.5 6.0 5.5 5.0 4.5 4.0 3.5 3.0 2.5 2.0 1.5 1.0 0.5 0.0 -0.5 -1.0 -1.5
Chemical shift (ppm)

(*E*)-**3i**
